# Supplementary material for: Plasma proteome variation and its genetic determinants in children and adolescents
Source: Nat Genet. 2025 Feb 19;57(3):635–46. doi: 10.1038/s41588-025-02089-2 (PMC11906355; doi:10.1038/s41588-025-02089-2)
Supplement: Supplementary file 1 — Supplementary Notes 1–8 and Supplementary Figs. 1–3. [file 41588_2025_2089_MOESM1_ESM.pdf]

nature genetics

Supplementary information

https://doi.org/10.1038/s41588-025-02089-2

Plasma proteome variation and its genetic determinants in children and adolescents

In the format provided by the authors and unedited

Table of Contents

Supplementary Note 1: Baseline participant characteristics stratified by sub-group and sex..........2
Supplementary Note 2: Plasma proteomics sample preparation 4
Supplementary Note 3: Association of ANGPTL3 with BMI-SDS in pediatric population 5
Supplementary Note 4: Definition and refinement of significant loci...6
Supplementary Note 5: Quantile–quantile plot for all proteins with pQTLs 7
Supplementary Note 6: Comparison of measurements across instruments and time points.........44
Supplementary Note 7: GWAS summary statistics datasets used in this study..............................45
Supplementary Note 8: Replication of pQTLs in children and adults 49
References for supplementary notes...49
Supplementary Figure 1 51
Supplementary Figure 2 52
Supplementary Figure 3 53

Supplementary Note 1: Baseline participant characteristics stratified by sub-group and sex

	General population			Obesity clinic		
	Male (n=421)	Female (n=530)	*Adjusted p-value	Male (n=538)	Female (641)	*Adjusted p-value
Age, mean, y	12 (2.9)	12 (3.3)	0.924	12 (2.7)	12 (3.2)	0.862
Tanner stage 1/2-5, No. (%)	88/181 (33/67)	145/293 (33/67)	-	162/216 (43/57)	165/353 (32/68)	-
BMI, kg/m2	17.6 (2.1)	17.8 (2.4)	0.57	27.2 (5.4)	27.1 (5.7)	0.837
BMI SDS	0 (0.8)	-0.1 (0.8)	0.569	3 (0.8)	2.6 (0.6)	<0.001
ALT, U/L	20.9 (5.8)	19 (6.2)	<0.001	30.9 (19.7)	26.7 (16.5)	<0.001
AST, U/L	28.5 (7.2)	25.5 (7.6)	<0.001	27.5 (11.4)	24.8 (9.7)	<0.001
GGT, U/L	16.4 (4.6)	15.2 (4.6)	<0.001	20.1 (11.2)	18.1 (9.3)	0.002
Glucose, mmol/L	5 (0.4)	4.9 (0.4)	0.015	5.1 (0.4)	5 (0.5)	0.032
Insulin, pmol/L	49.7 (24.5)	59.4 (27)	<0.001	90.1 (59.1)	99.1 (68.7)	0.032
HbA1c, mmol/mol	33.7 (2.8)	33.5 (3)	0.372	33.6 (2.8)	33.9 (3.4)	0.335
Triglycerides, mmol/L	0.6 (0.3)	0.7 (0.3)	0.075	1 (0.6)	1 (0.5)	0.813
Total cholesterol, mmol/L	3.9 (0.7)	4 (0.7)	0.079	4.1 (0.8)	4.1 (0.8)	0.443
LDL cholesterol, mmol/L	2.1 (0.5)	2.1 (0.6)	0.108	2.4 (0.7)	2.4 (0.7)	0.443
HDL cholesterol, mmol/L	1.6 (0.4)	1.6 (0.3)	0.924	1.3 (0.3)	1.3 (0.3)	0.951

Abbreviations: BMI=body mass index; SDS=standard deviation score; ALT=alanine aminotransferase; AST=aspartate aminotransferase; GGT=gamma glutamyl transferase; HbA1C=hemoglobin A1c; LDL=low-density lipoprotein; HDL=high-density

lipoprotein. *Note:* Values are in the form of mean (standard deviation), unless where otherwise is noted. P-values are calculated using two-sided Welch's t-test adjusted for multiple hypothesis testing by the Benjamini-Hochberg procedure.

Supplementary Note 2: Plasma proteomics sample preparation

Plasma samples were prepared on an automated liquid handling system (Agilent Bravo) in a 96-well plate format. Specifically, 45 µl of lysis buffer (10 mM tris(2-carboxyethyl)phosphine, 40 mM chloroacetamide, 100 mM Tris, pH 8.5) was added to 5 µl of blood plasma sample to achieve a tenfold dilution (plasma plate). The diluted solution was thoroughly mixed by pipetting 50 times up and down, for a volume of 40 µl. The plate was then centrifuged up to 300*g*, and 5 µl of the tenfold-diluted plasma was pipetted into a new 96-well plate filled with 15 µl of lysis buffer in each well (digestion plate). The digestion plate was heated to 90 °C for 10 min, followed by cooling to room temperature for 5 min. The denatured protein mixture was digested at 37 °C for 2 h after the addition of 20 µl of a freshly prepared 0.05 $\mu g\ \mu l^{-1}$ trypsin/LysC mixture into each well, to a final volume of 40 µl (1 µg of enzyme to 40 µg protein, with an estimated 80 $\mu g\ \mu l^{-1}$ protein concentration in plasma). Enzymatic digestion was quenched by the addition of 64 µl of 0.2% trifluoroacetic acid (TFA) then thoroughly mixed by pipetting 20 times up and down. A total of 250 ng of digestion mixture was loaded onto a disposable Evotip C18 trap column (Evosep Biosystems) according to the manufacturer's instructions. Briefly, Evotips were wetted with 2-propanol, activated with 20µl 0.1% formic acid in acetonitrile, equilibrated with 20µl 0.1% formic acid and then loaded using centrifugal force at 1,000*g* for 2 min. Evotips were then washed with 20µl 0.1% formic acid followed by the addition of 100 µl of 0.1% formic acid to prevent drying.

Supplementary Note 3: Association of ANGPTL3 with BMI-SDS in pediatric population

We found that levels of ANGPTL3, a potent regulator of blood triglyceride levels, are inversely correlated with BMI-SDS (**Supplementary Table 2**). The relationship between ANGPTL3 and obesity or BMI has yielded mixed results[1]. Some studies report elevated circulating levels of ANGPTL3 in patients with obesity or a positive correlation with BMI[2,3], while others show this is not the case in individuals with metabolically healthy obesity[1] or even report the opposite trend[4]. The reasons for these discrepancies remain unclear, though differences in underlying metabolic conditions have been suggested as potential explanations. Our data add new observations in children and adolescents, an underrepresented population, contributing to the ongoing exploration of the complex relationship between ANGPTL3 and BMI.

Supplementary Note 4: Definition and refinement of significant loci

We reported the total number of identified significant associations using both a conservative multiple comparison-corrected threshold of $p<4.1x10^{-11}$ ($5x10^{-8}$ Bonferroni-adjusted for 1,216 proteins tested) and the conventional genome-wide threshold of $5x10^{-8}$, the latter of which was used for all downstream analysis. We first identified linkage disequilibrium (LD)-independent signal clusters for pQTLs by LD-based clumping implemented in PLINK (v1.90b6.24)[5] with the following settings: --clump-r2 0.2 --clump-kb 1000 --clump-p1 5e-8, and treating the human leukocyte antigen (HLA) region (chr6: 29691116–33054976 for hg19) as one locus, for which we report one representative SNP-protein association with the lowest P-value. We then performed approximate conditional analysis using GCTA-COJO (v1.93.3) to identify conditionally independent loci, to which we refer as conditionally independent pQTLs[6]. We defined a cis-pQTL variant as a SNP residing within 1Mb upstream or downstream (+/-1Mb) of the transcription start site (TSS) of the corresponding protein-coding gene. We extracted the TSS information for all proteins from BioMart (accessed on November 23, 2023) by mapping UniProt IDs (automatic) or gene names to UniParc IDs.

Previous research has utilized various methods to report the pQTLs. These methods can be classified into two main groups: LD- and distance-based clumping or grouping[7–10], as well as conditional analysis aimed at identifying independent pQTLs[11–17.] In this study, we reported the number of pQTLs using both approaches, but the main results are based on LD- and distance-based clumping. To account for long-range LD that may have been overlooked by this approach, we calculated the pairwise LD between all primary pQTLs for the same protein. The results showed that only a small percentage (2%) had an r^2 greater than 0.2, indicating a low level of correlation between the primary pQTLs identified in this study. We note that the lack of unified reporting standards in pQTL studies hinders direct cross-study comparisons. Therefore, we contextualize our findings with those of previous studies by comparing the number of proteins with identified pQTLs, rather than the number of pQTLs themselves.

Supplementary Note 5: Quantile–quantile plot for all proteins with pQTLs

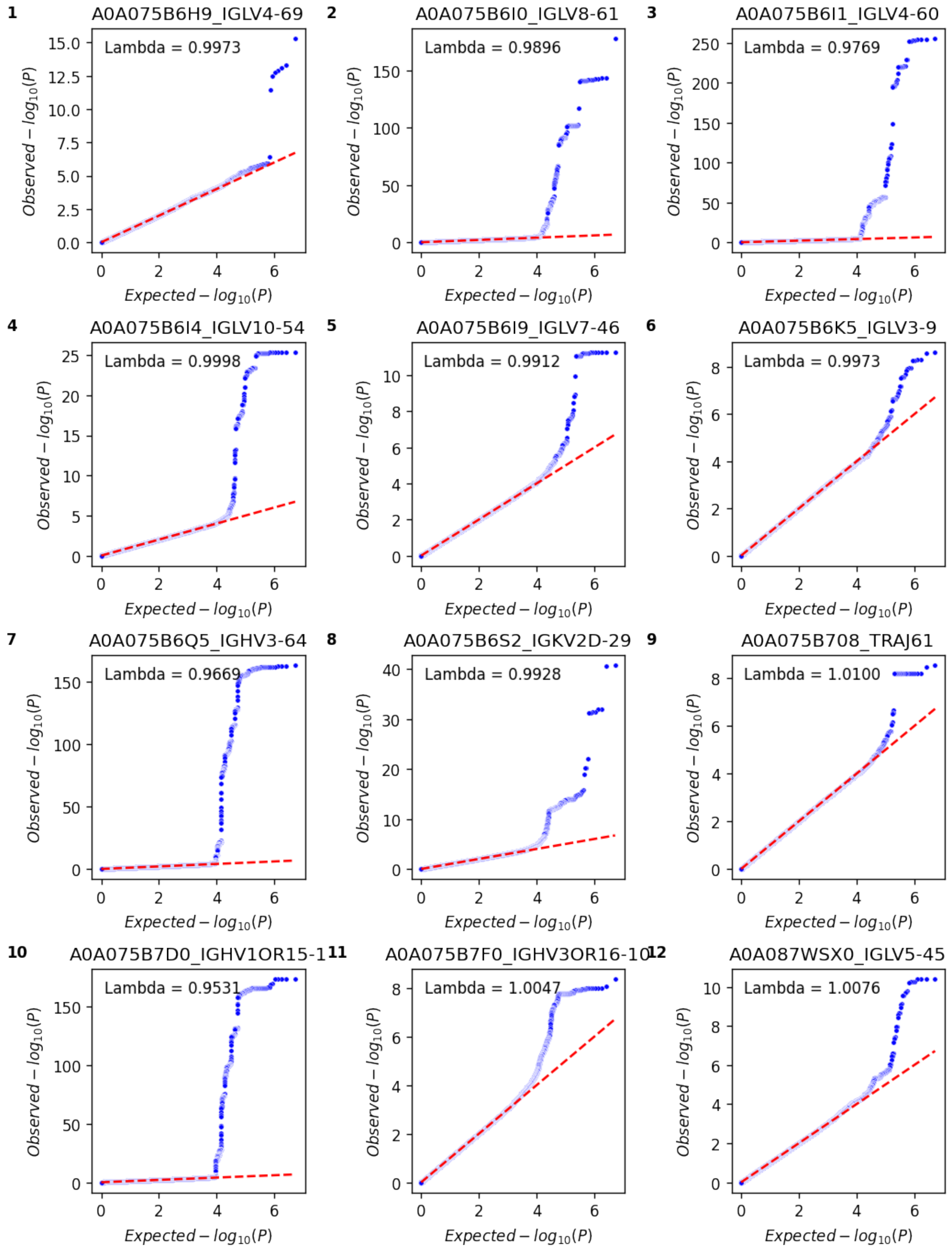

13

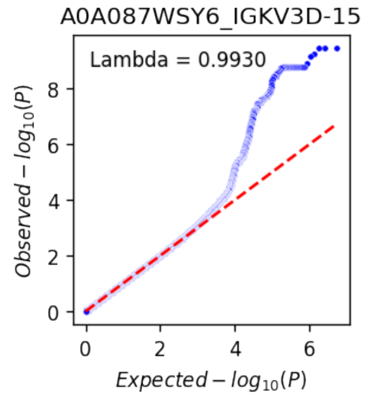

14

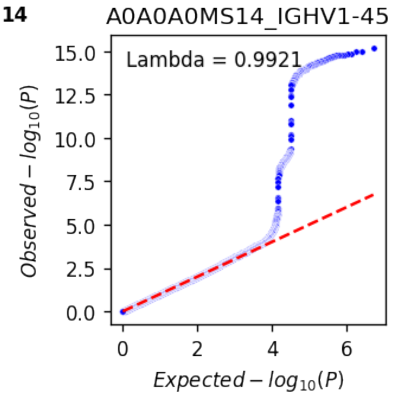

15

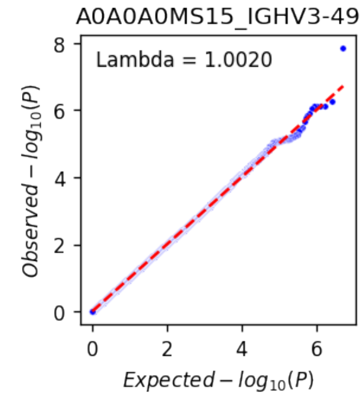

16

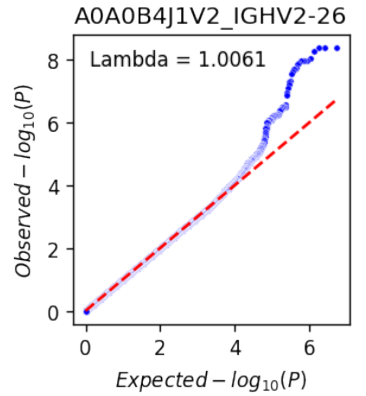

17

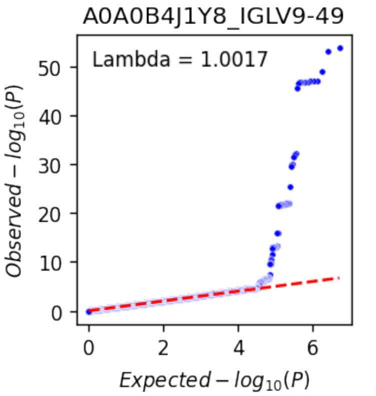

18

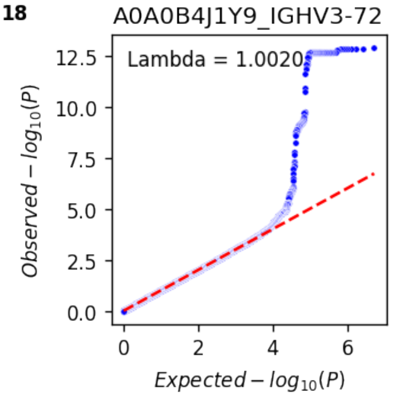

19

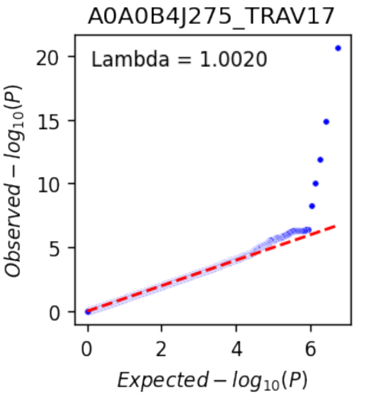

20

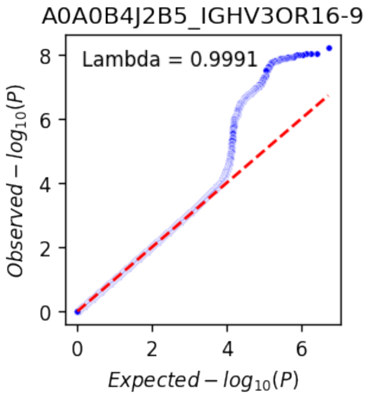

21

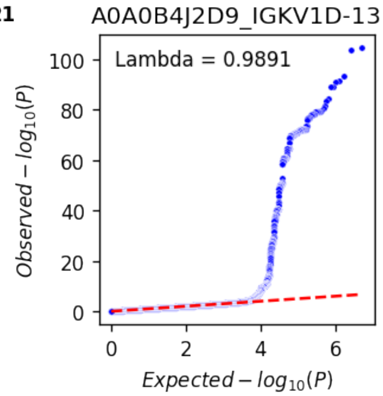

22

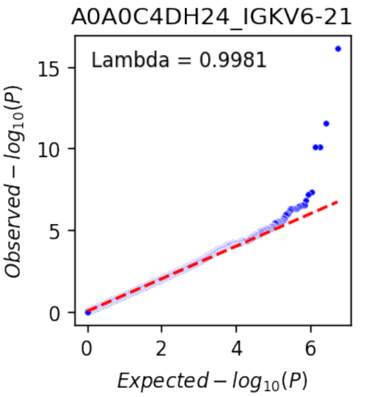

23

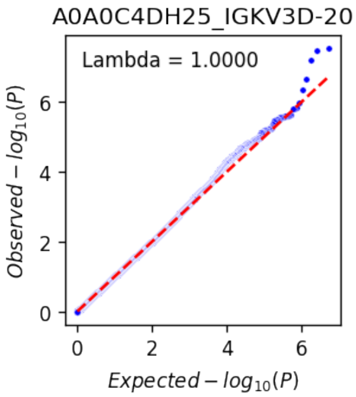

24

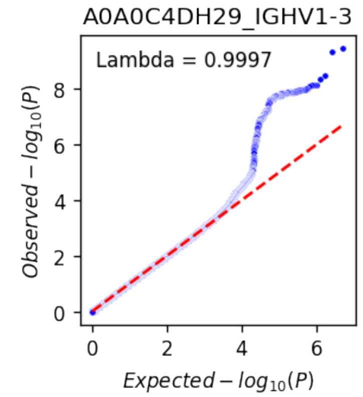

25

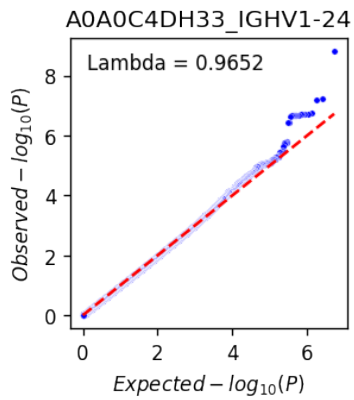

26

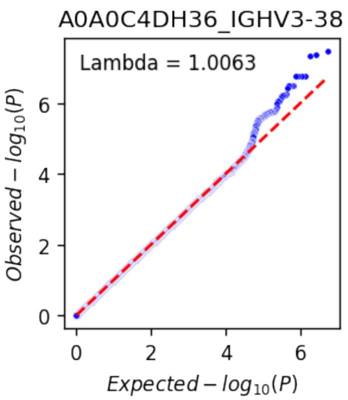

27

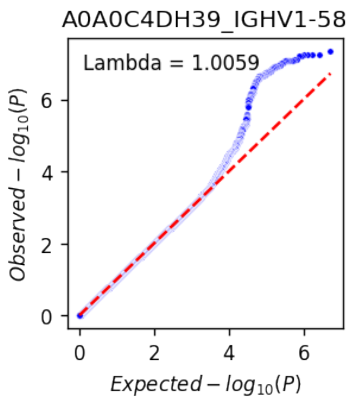

28

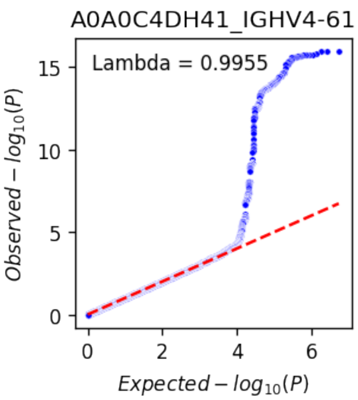

29

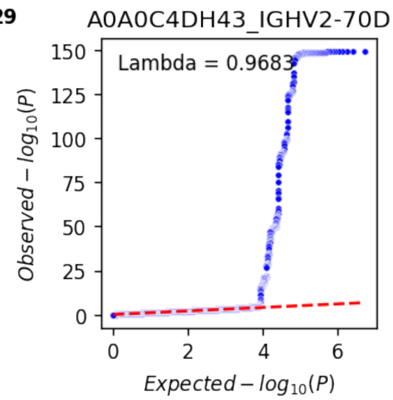

30

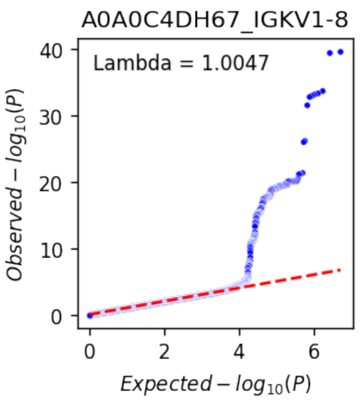

31

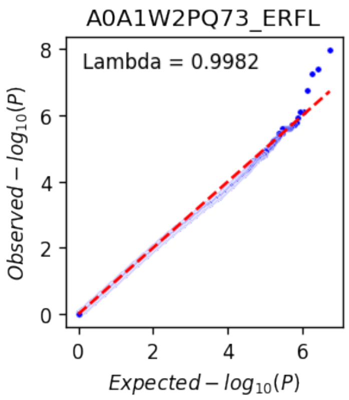

32

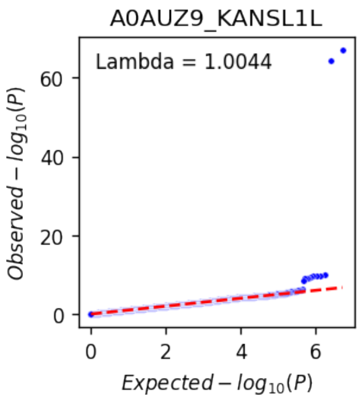

33

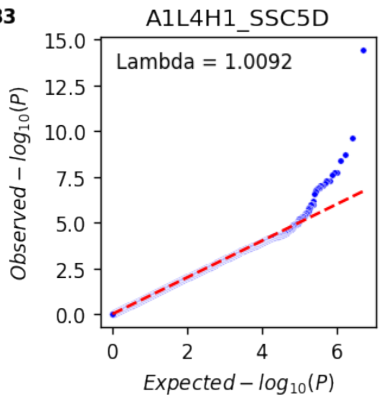

34

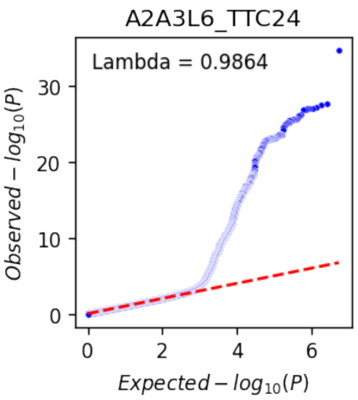

35

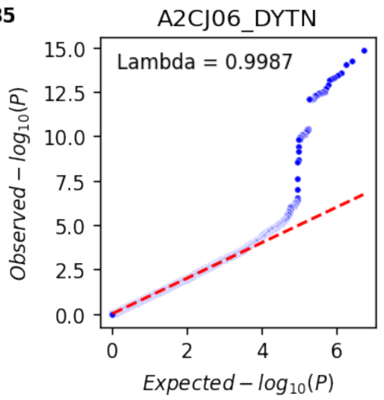

36

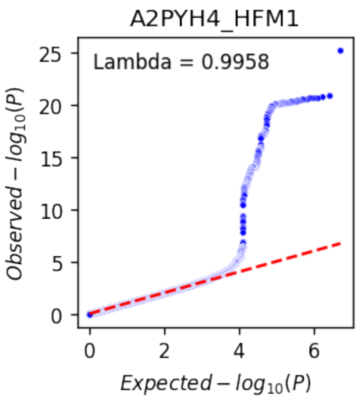

37

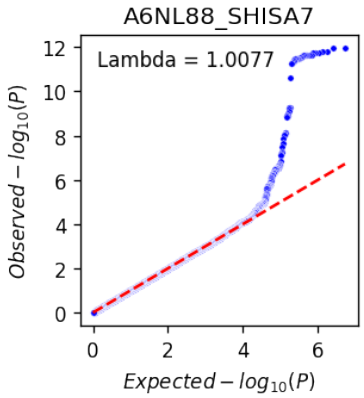

38

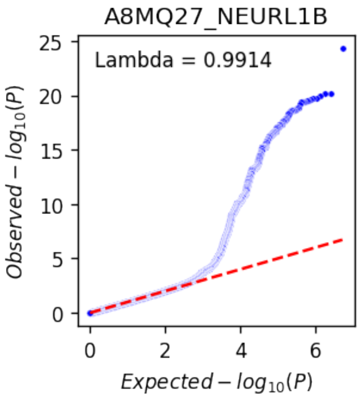

39

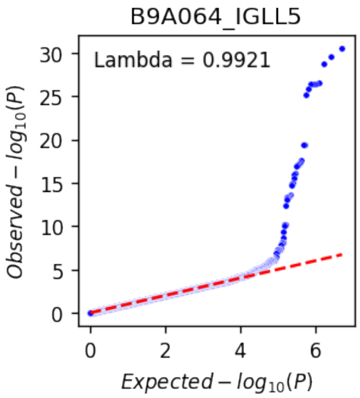

40

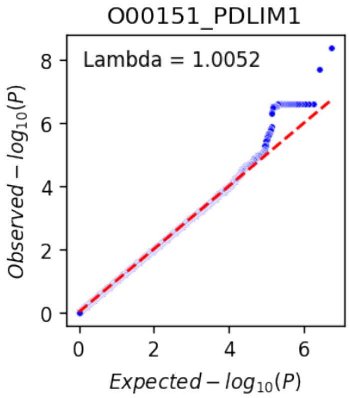

41

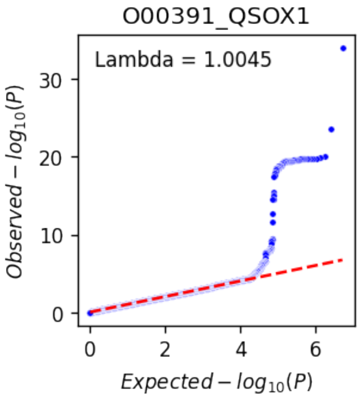

42

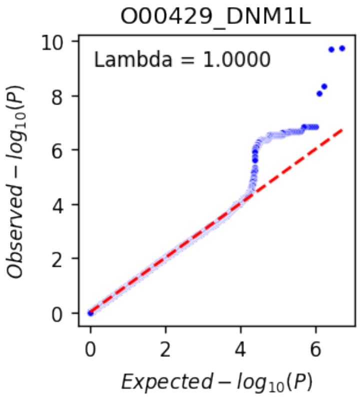

43

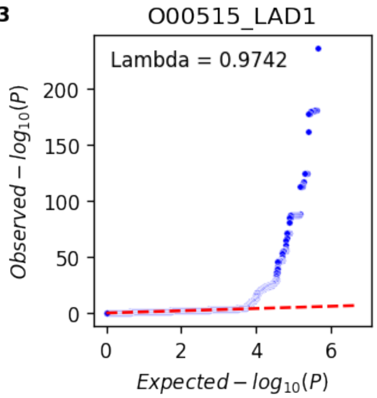

44

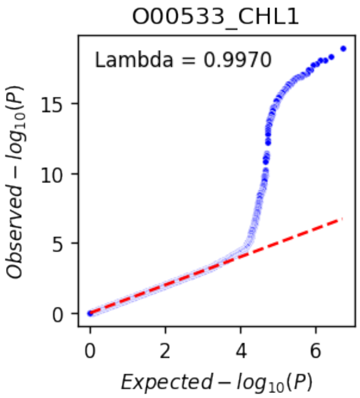

45

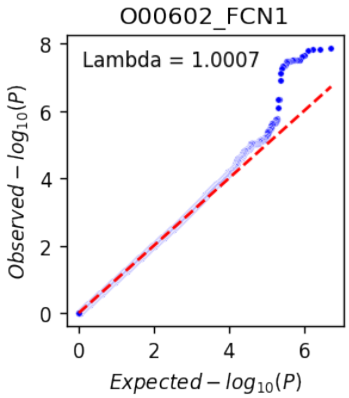

46

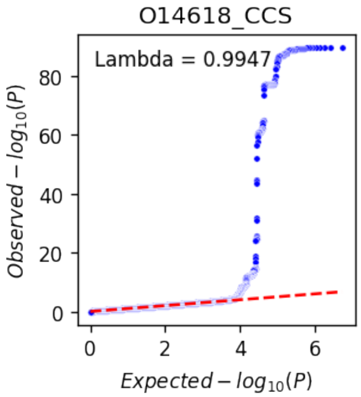

47

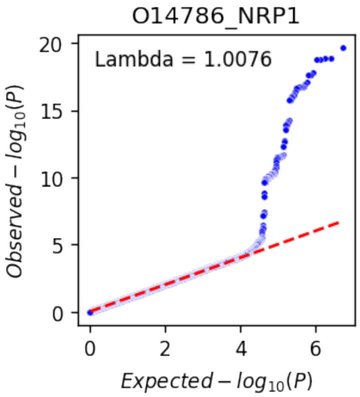

48

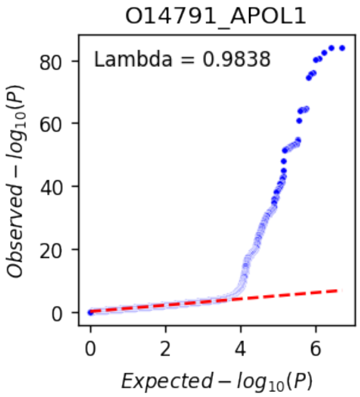

49

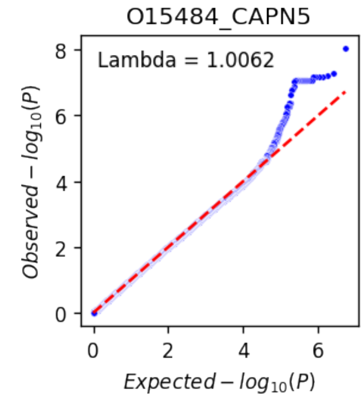

50

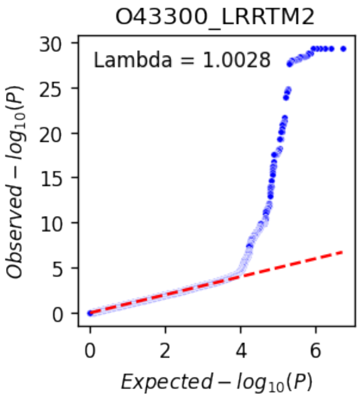

51

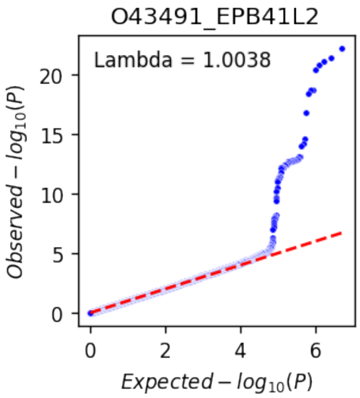

52

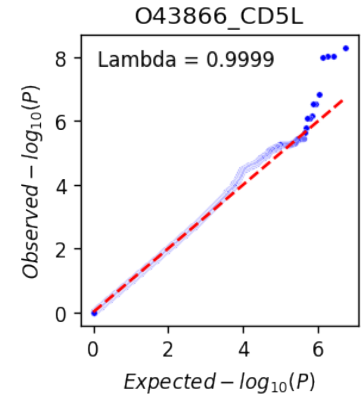

53

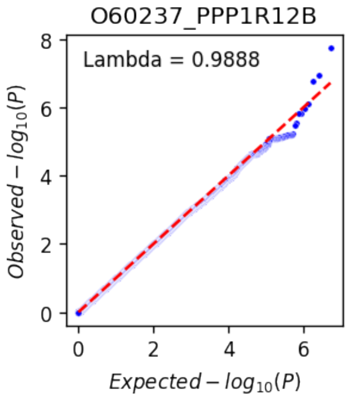

54

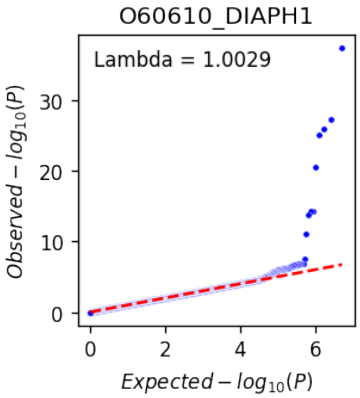

55

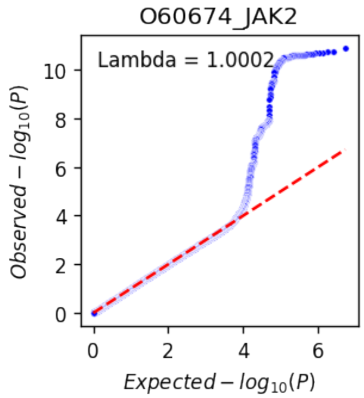

56

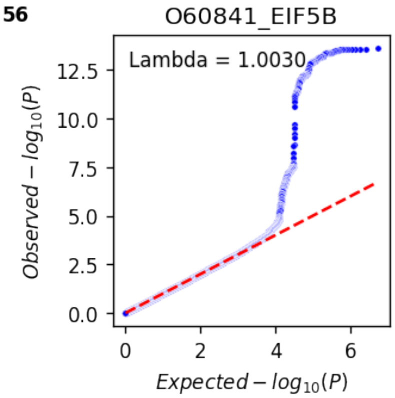

57

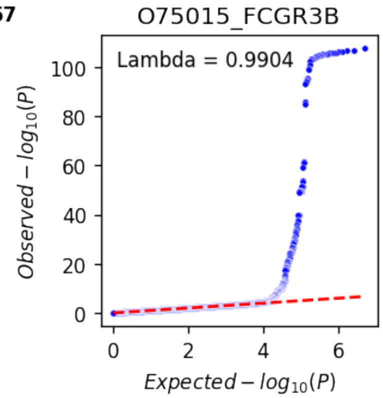

58

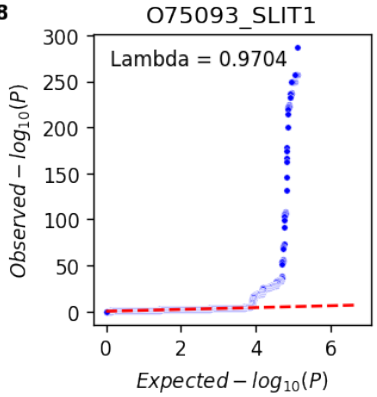

59

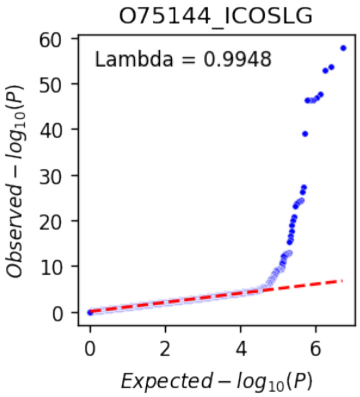

60

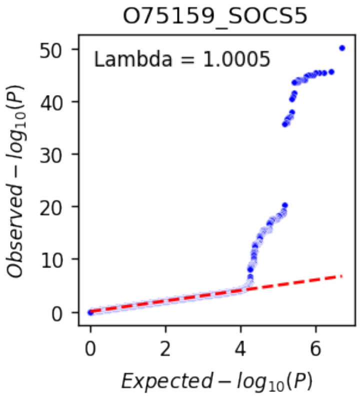

61

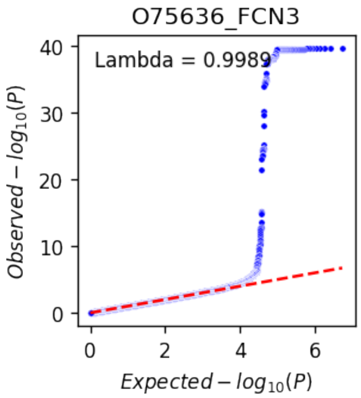

62

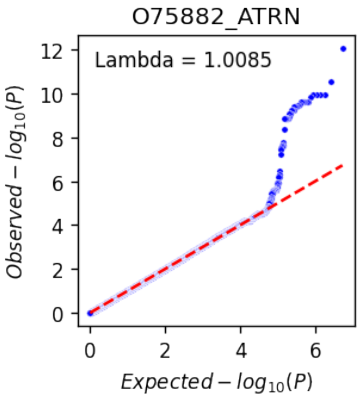

63

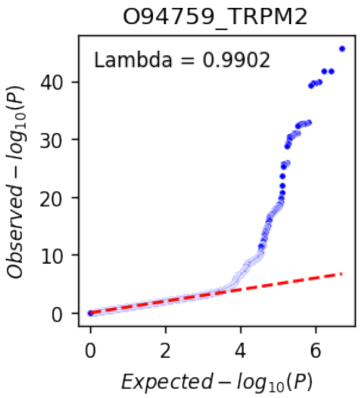

64

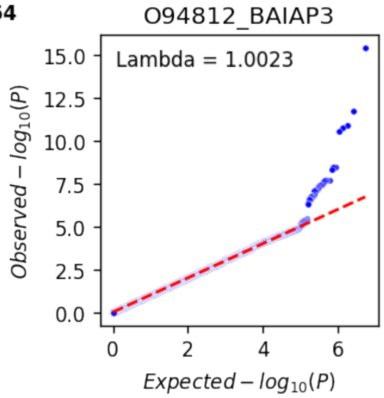

65

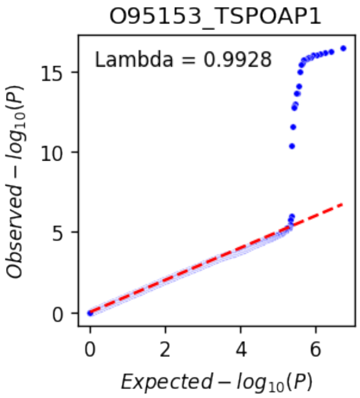

66

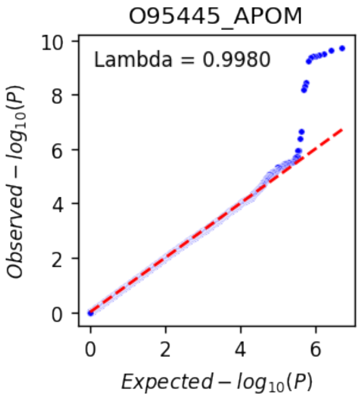

67

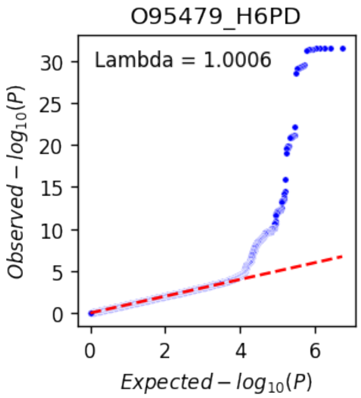

68

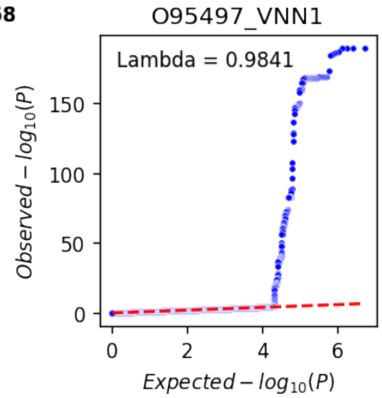

69

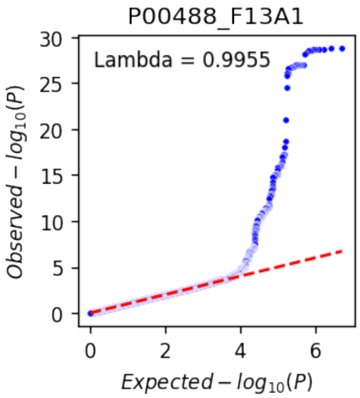

70

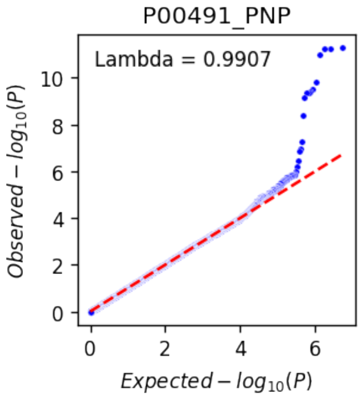

71

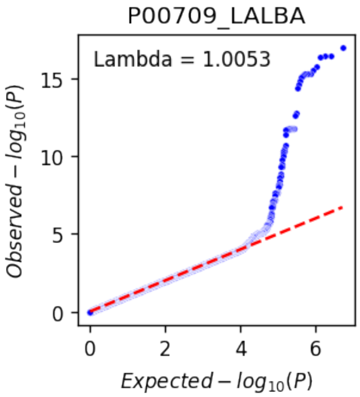

72

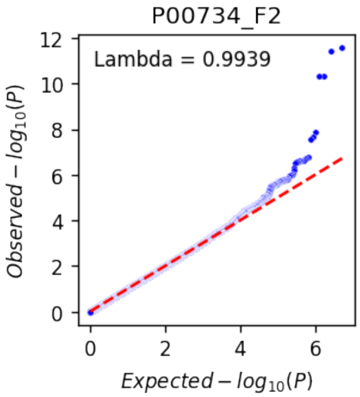

73
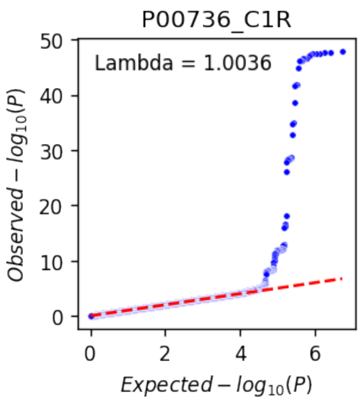

74
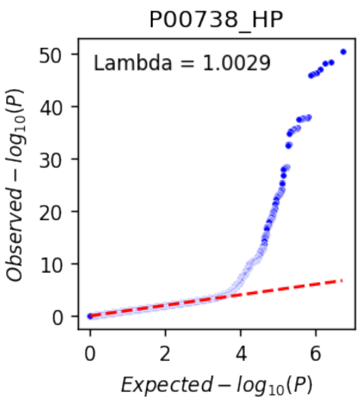

75
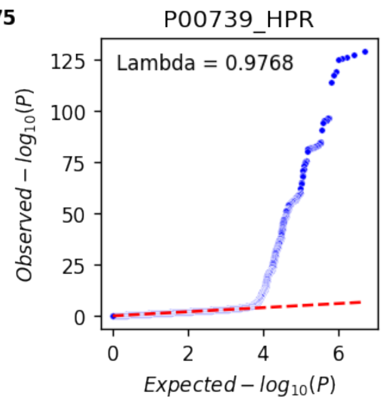

76
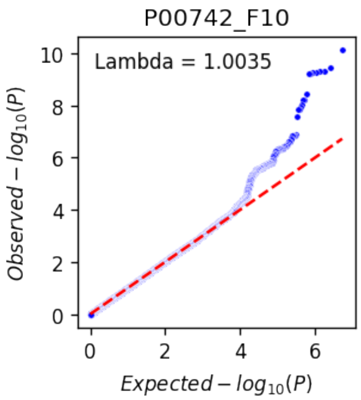

77
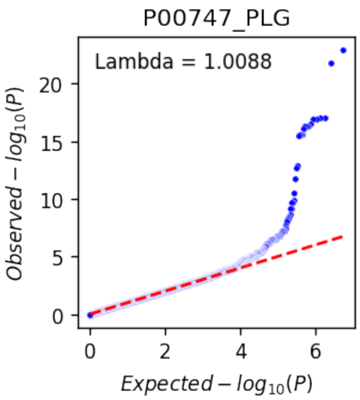

78
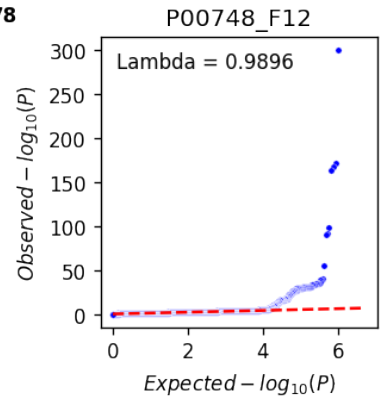

79
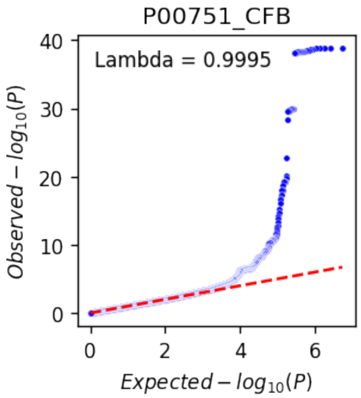

80
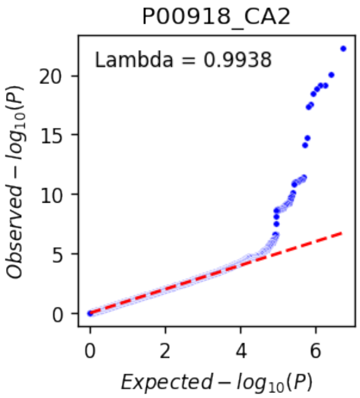

81
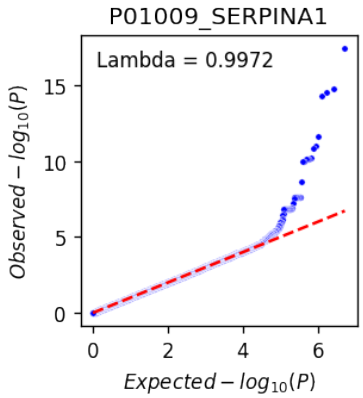

82
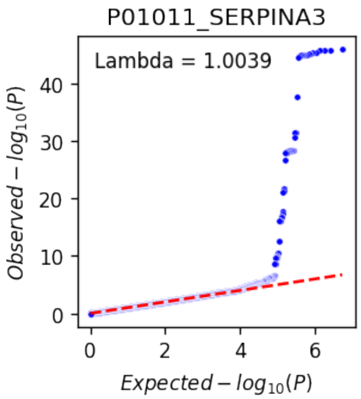

83
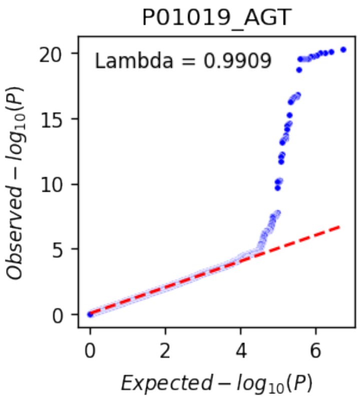

84
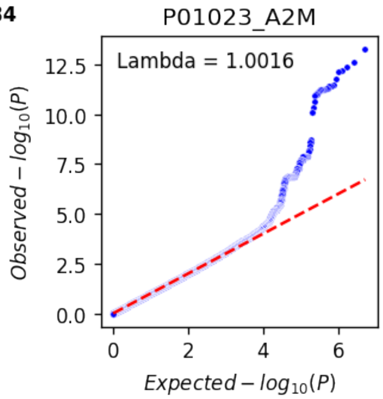

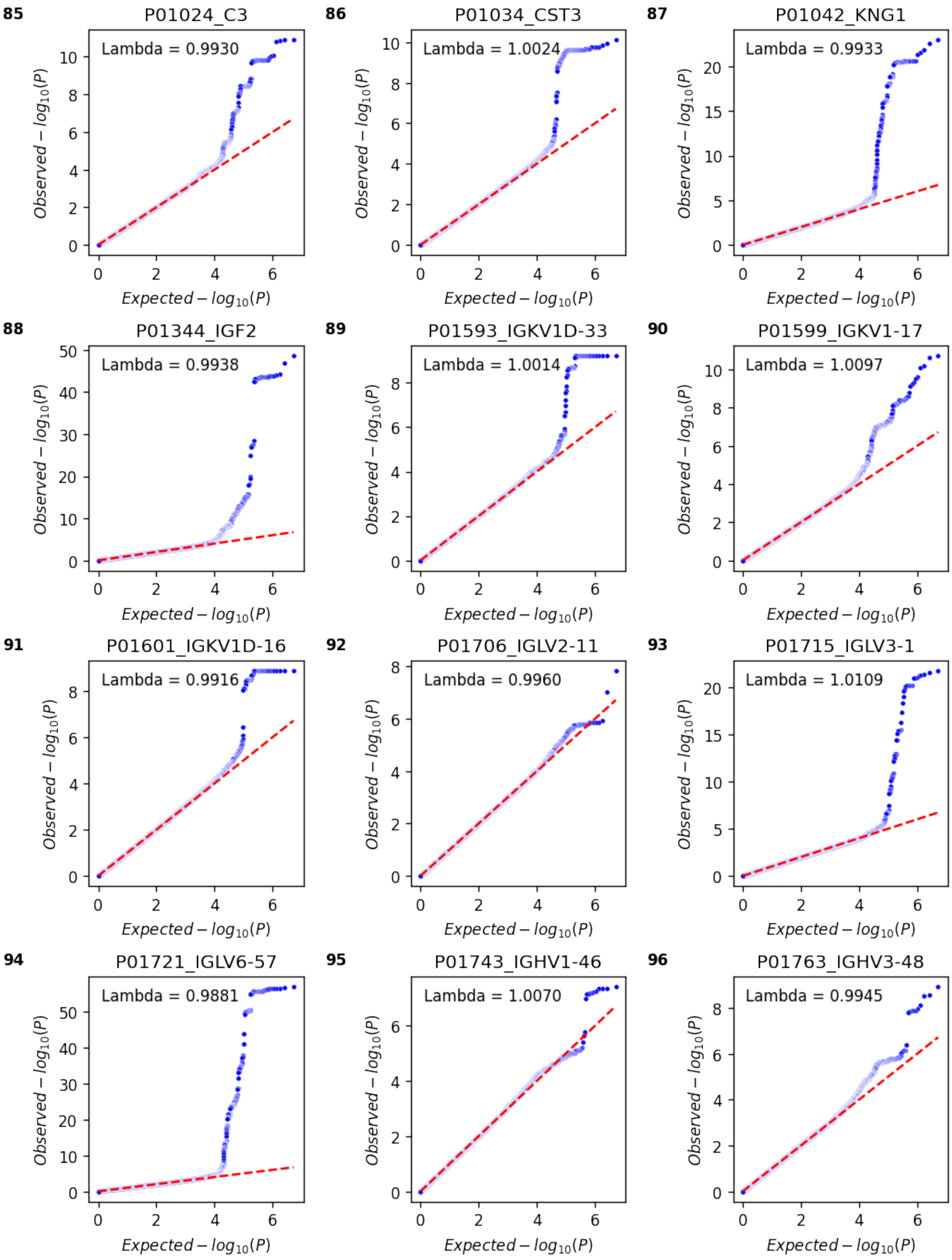
85
P01024_C3
Lambda = 0.9930
86
P01034_CST3
Lambda = 1.0024
87
P01042_KNG1
Lambda = 0.9933
88
P01344_IGF2
Lambda = 0.9938
89
P01593_IGKV1D-33
Lambda = 1.0014
90
P01599_IGKV1-17
Lambda = 1.0097
91
P01601_IGKV1D-16
Lambda = 0.9916
92
P01706_IGLV2-11
Lambda = 0.9960
93
P01715_IGLV3-1
Lambda = 1.0109
94
P01721_IGLV6-57
Lambda = 0.9881
95
P01743_IGHV1-46
Lambda = 1.0070
96
P01763_IGHV3-48
Lambda = 0.9945
$Observed - log_{10}(P)$
$Expected - log_{10}(P)$

97

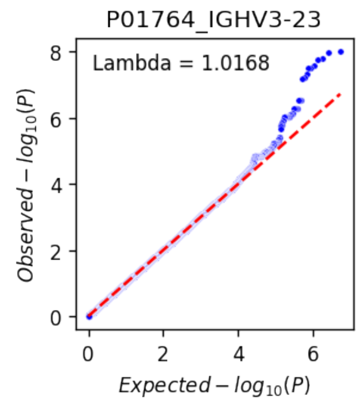

98

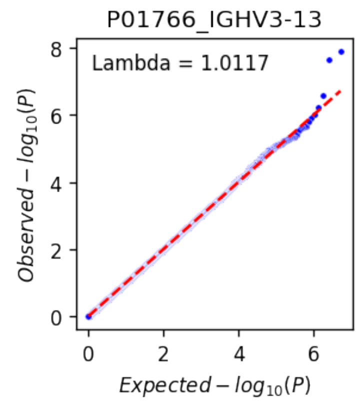

99

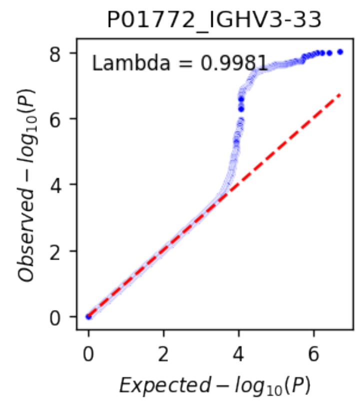

100

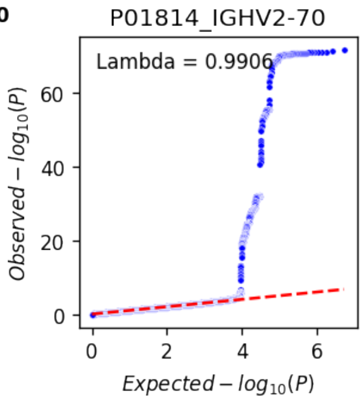

101

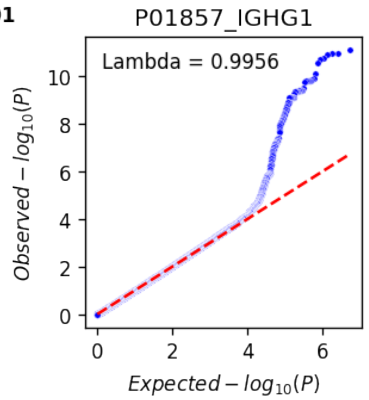

102

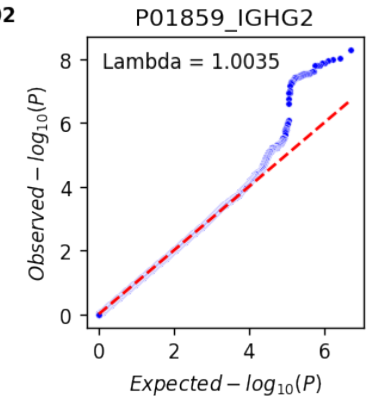

103

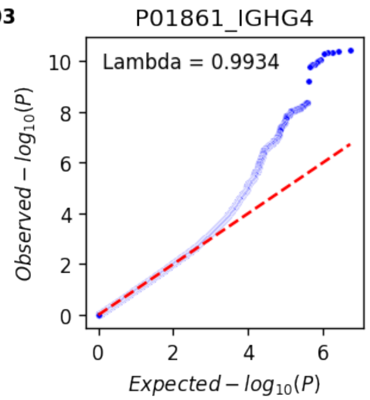

104

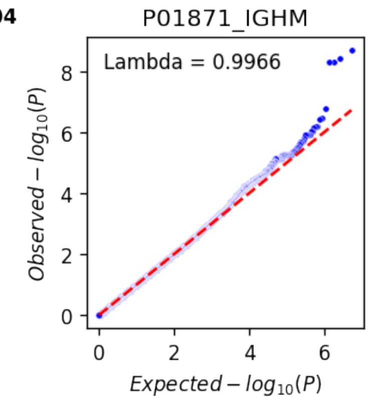

105

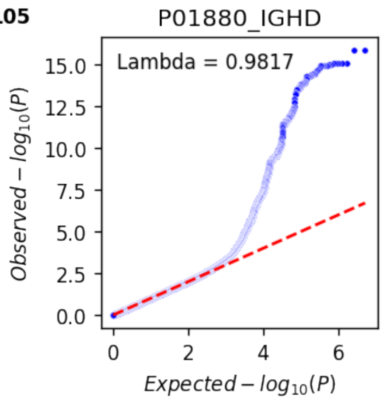

106

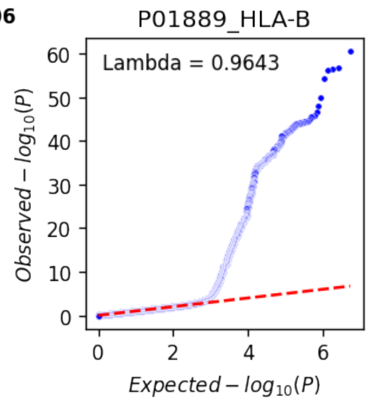

107

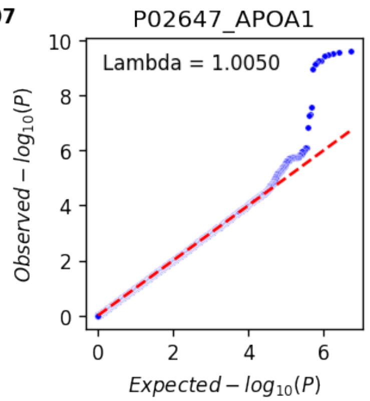

108

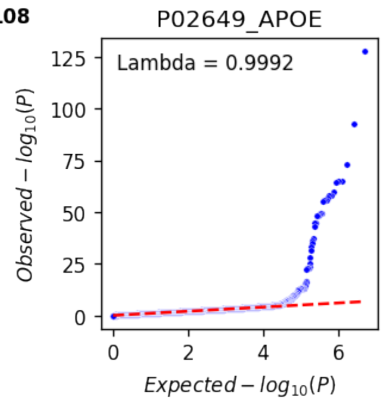

109
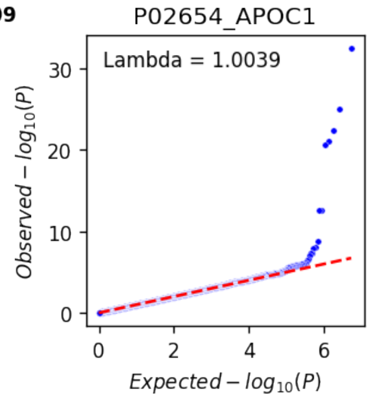

110
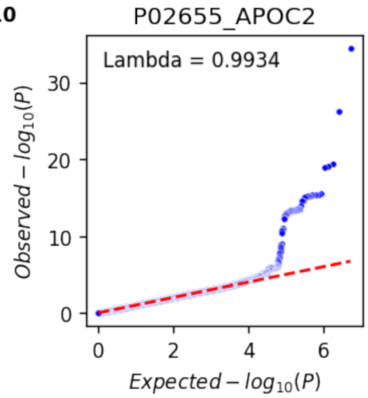

111
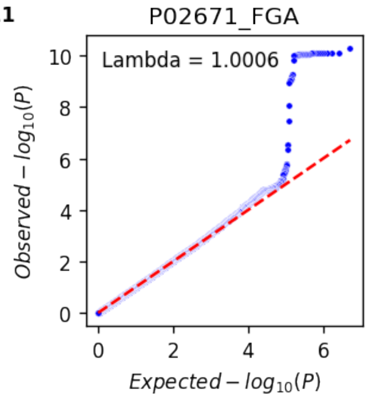

112
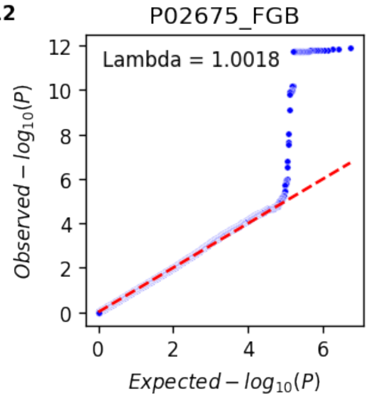

113
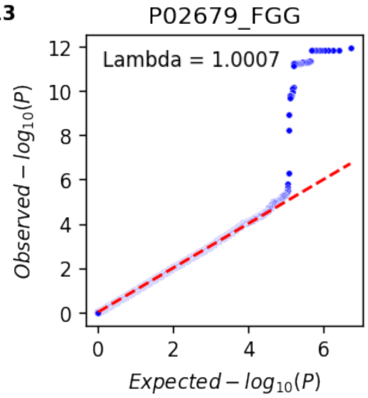

114
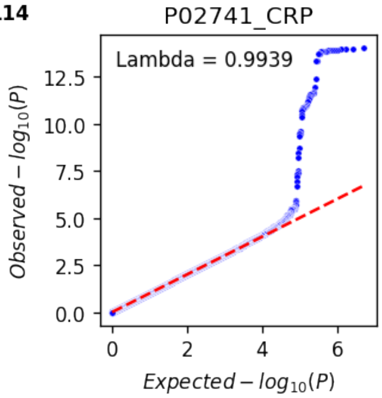

115
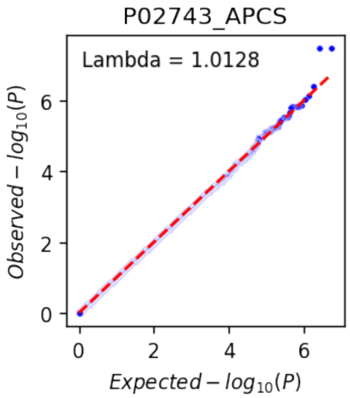

116
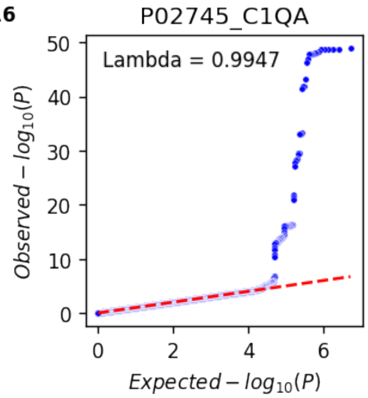

117
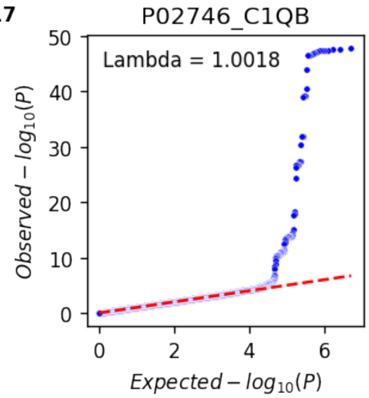

118
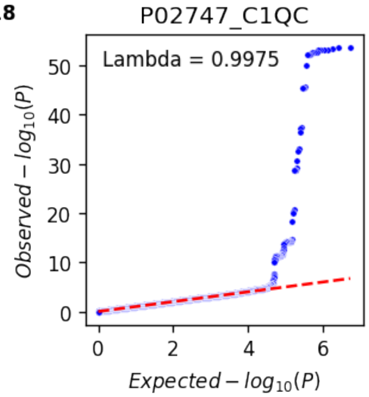

119
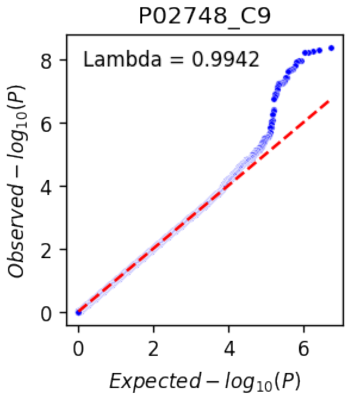

120
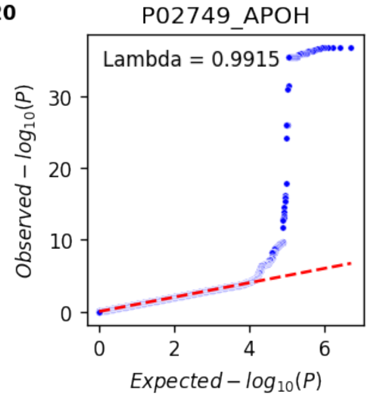

121

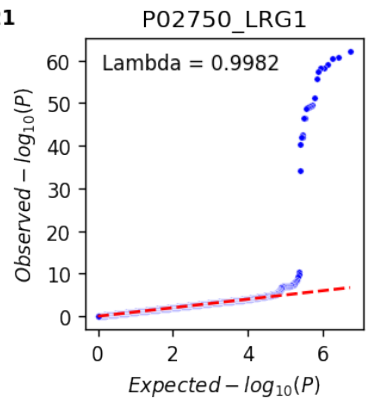

122

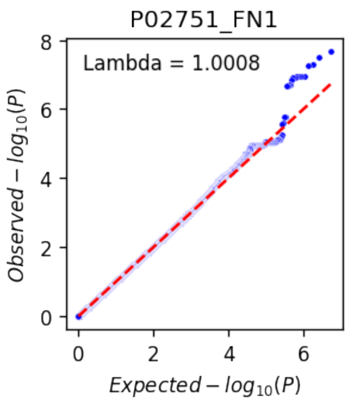

123

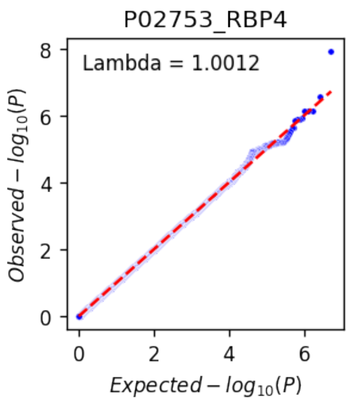

124

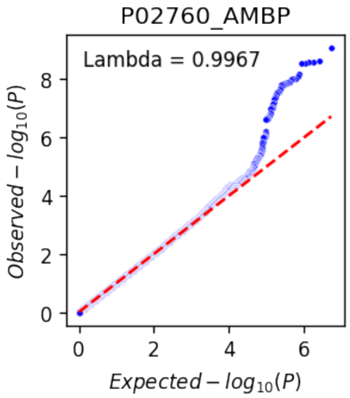

125

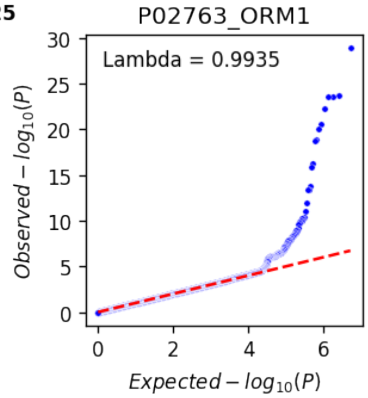

126

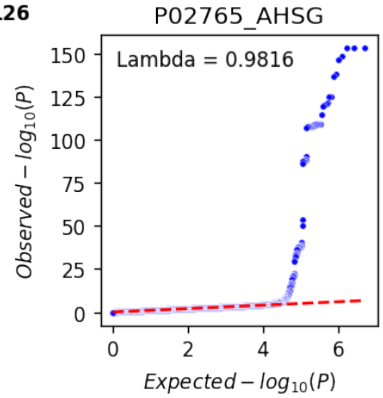

127

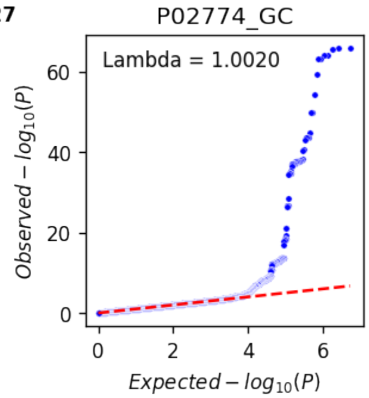

128

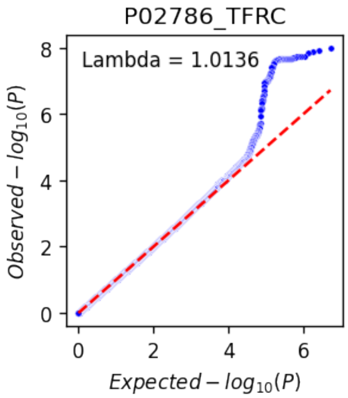

129

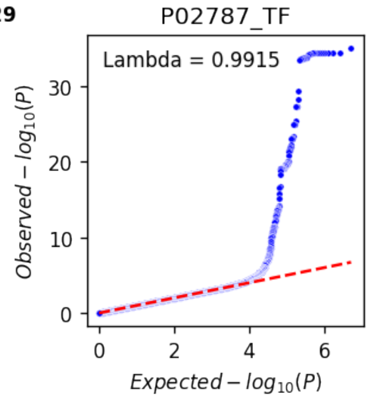

130

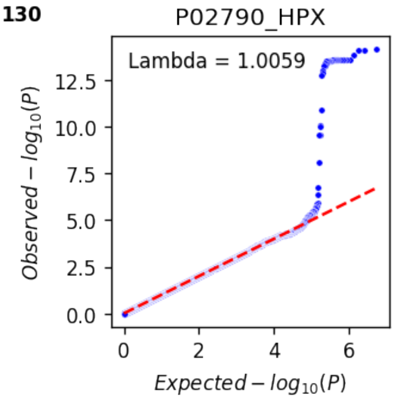

131

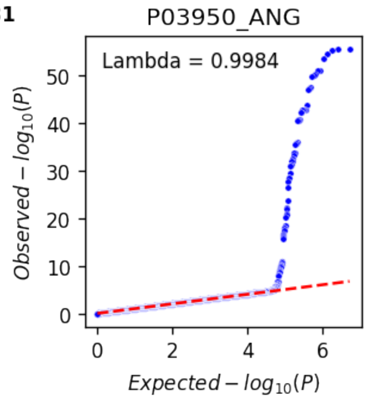

132

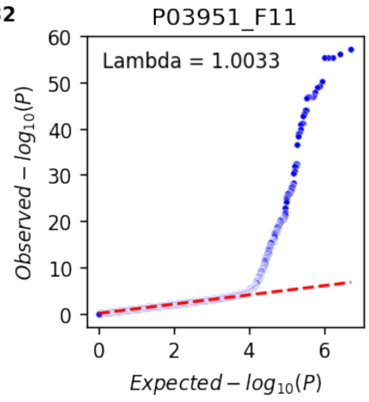

133
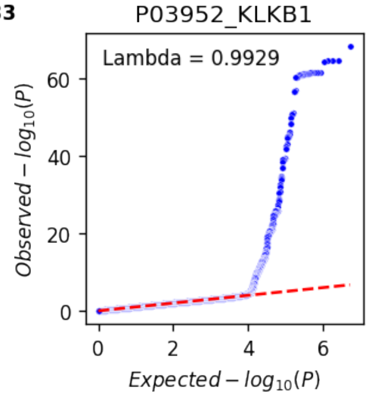

134
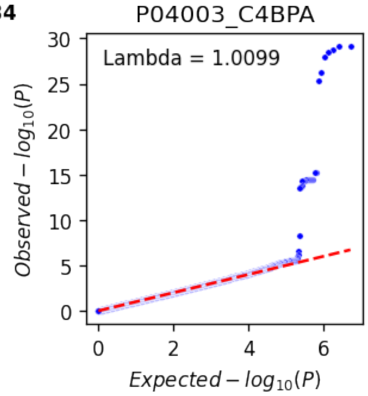

135
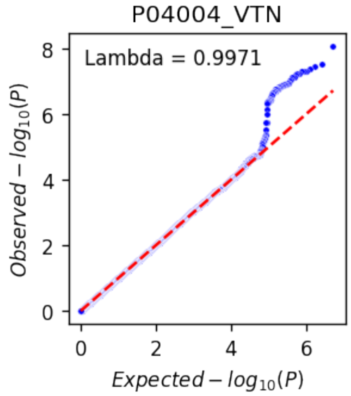

136
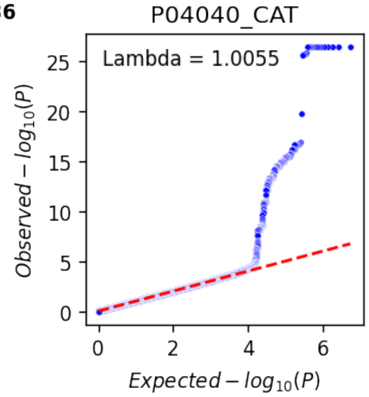

137
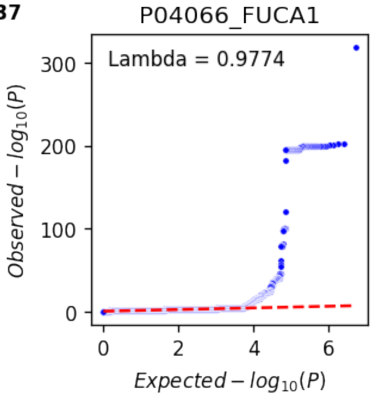

138
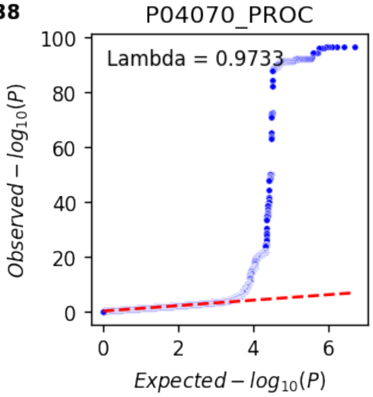

139
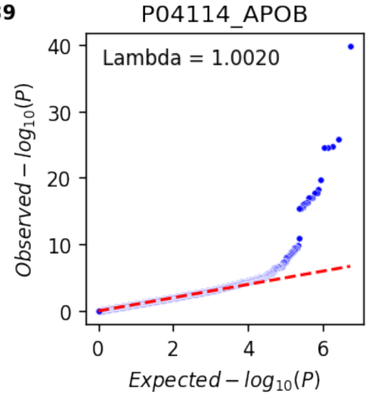

140
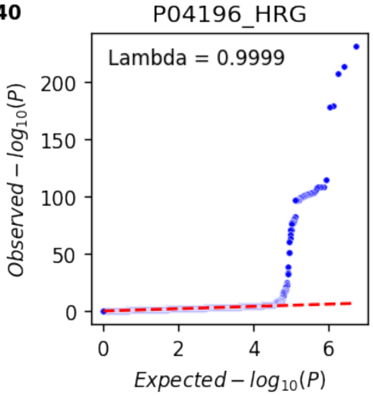

141
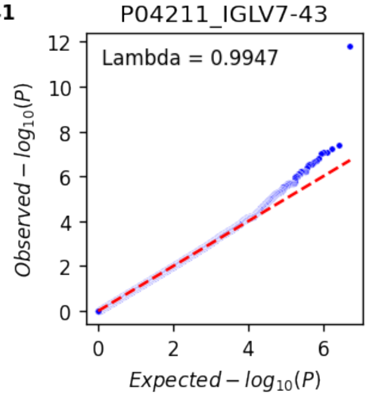

142
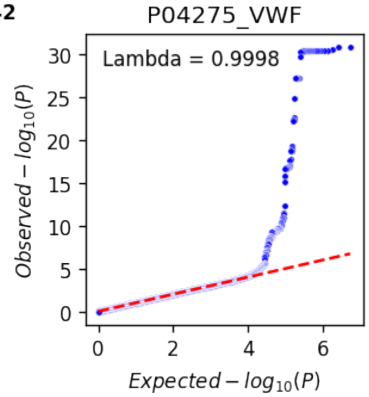

143
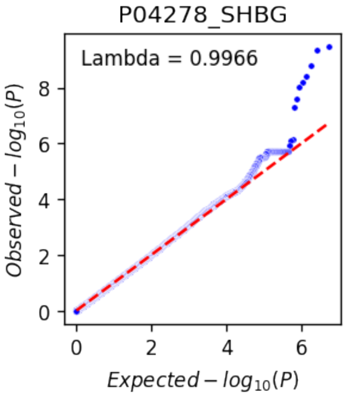

144
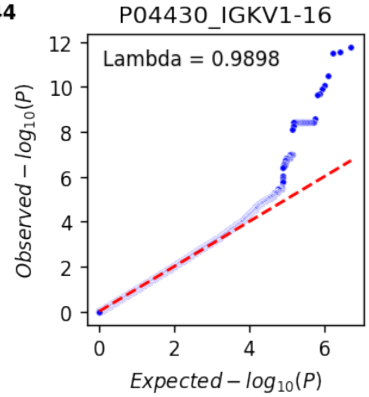

145
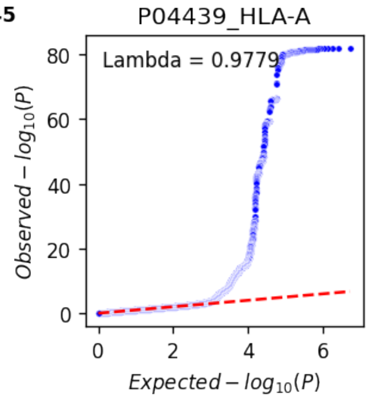

146
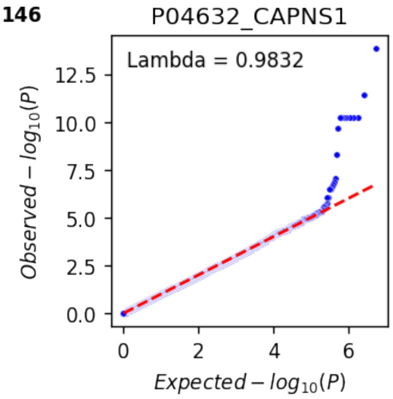

147
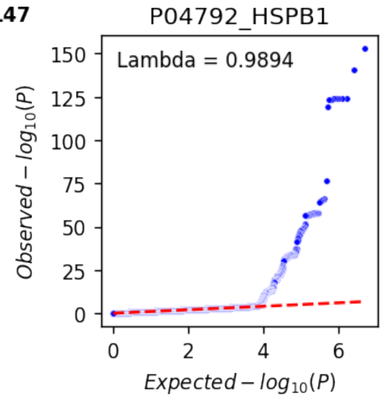

148
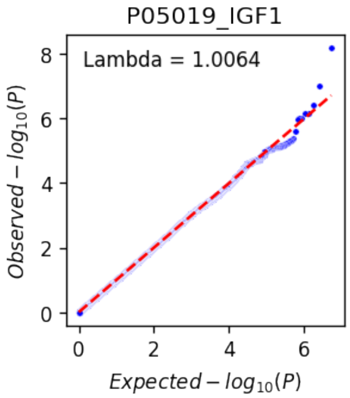

149
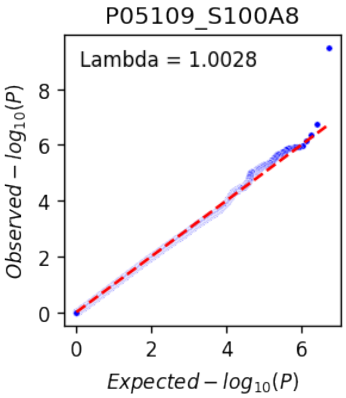

150
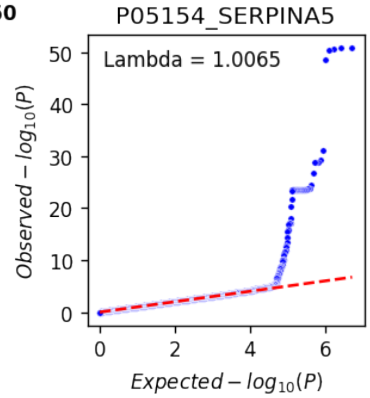

151
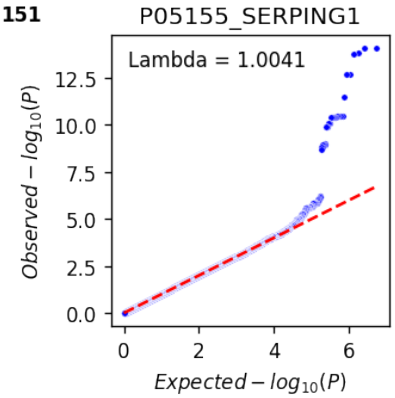

152
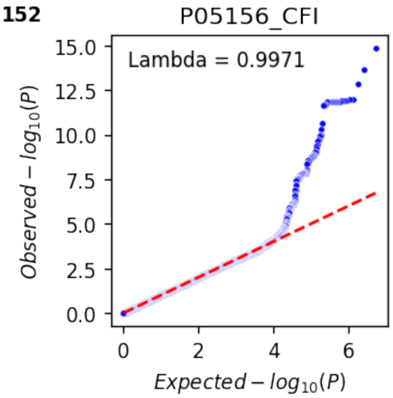

153
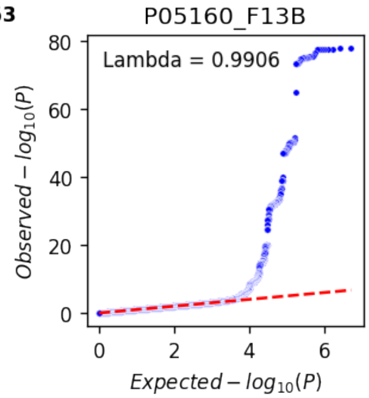

154
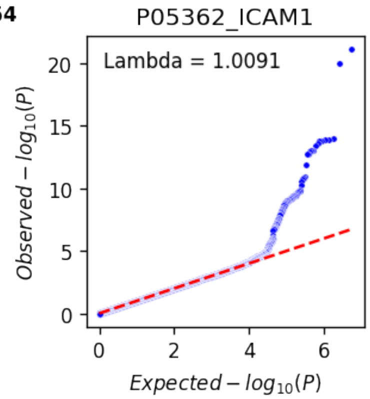

155
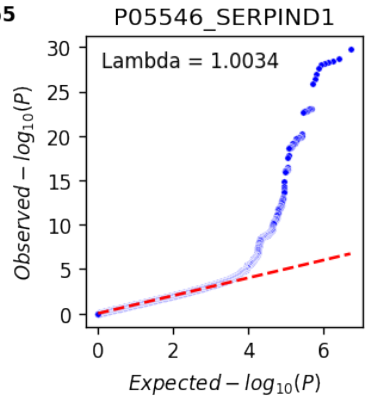

156
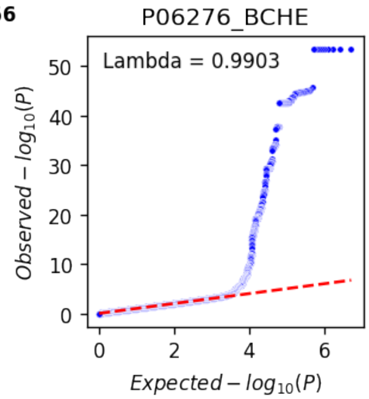

157

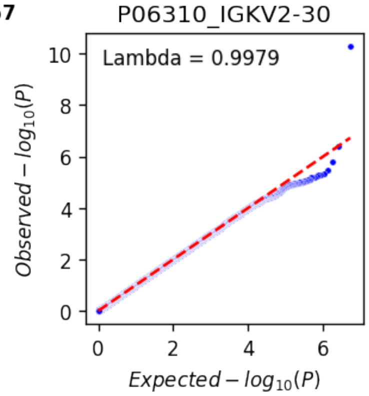

158

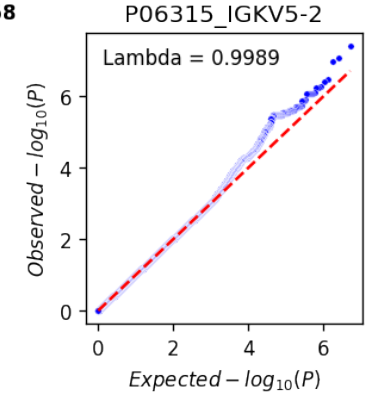

159

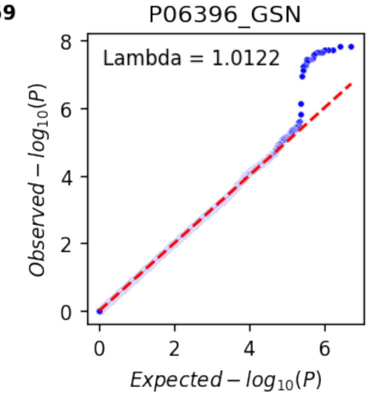

160

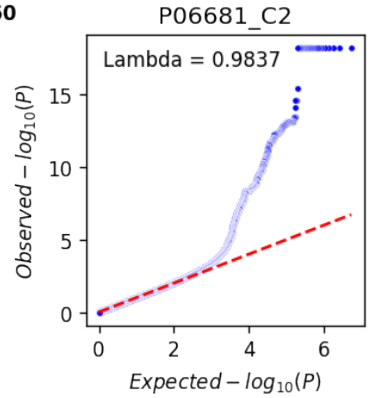

161

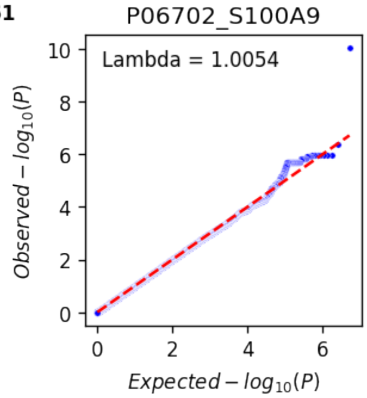

162

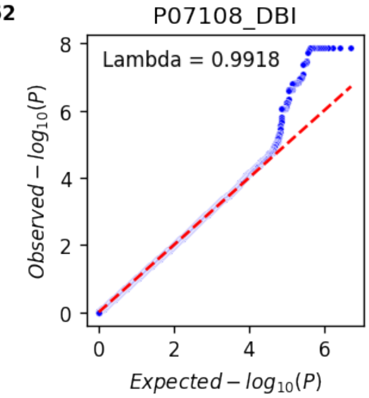

163

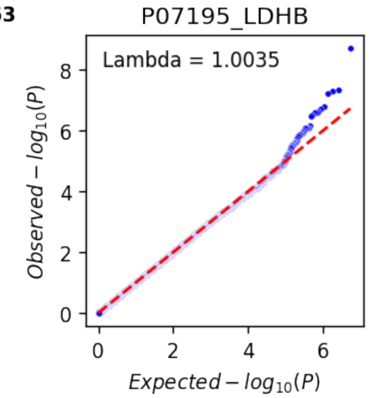

164

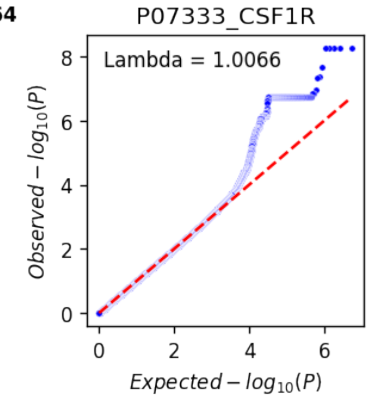

165

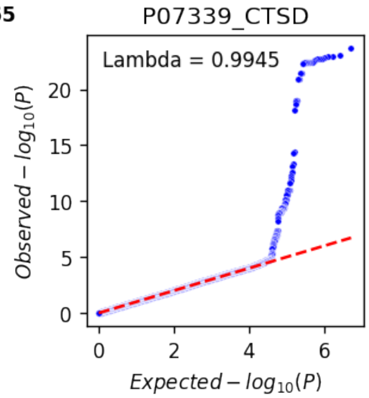

166

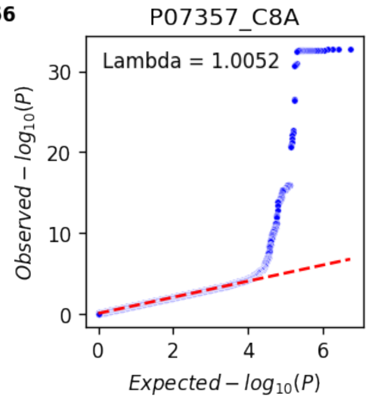

167

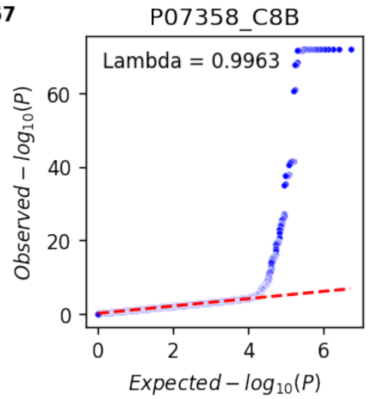

168

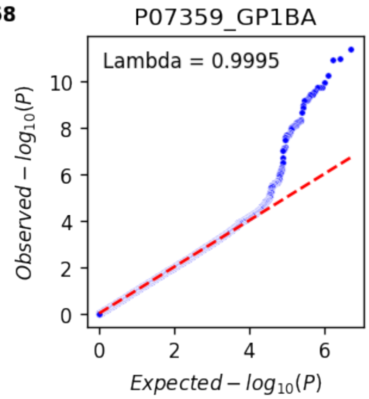

169
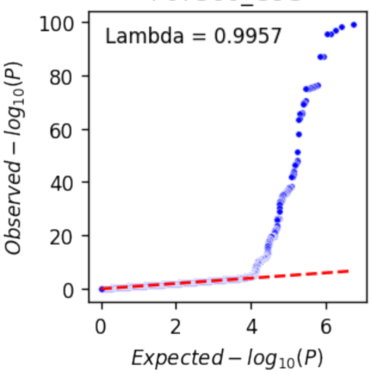

170
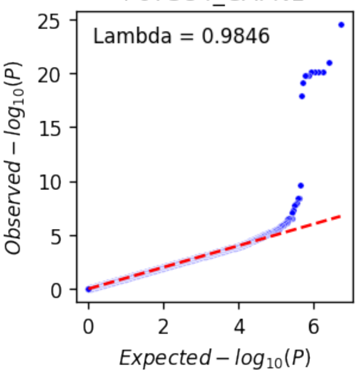

171
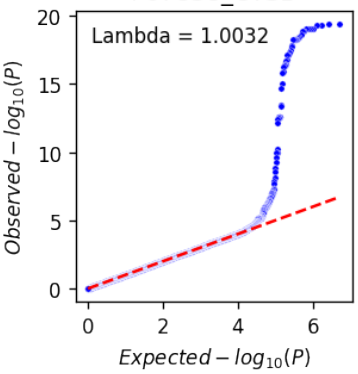

172
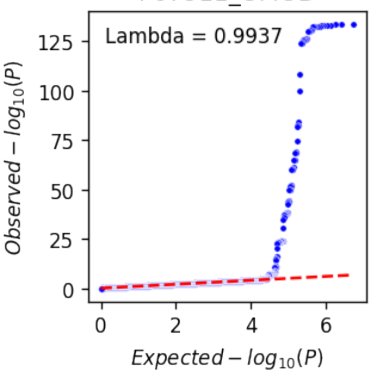

173
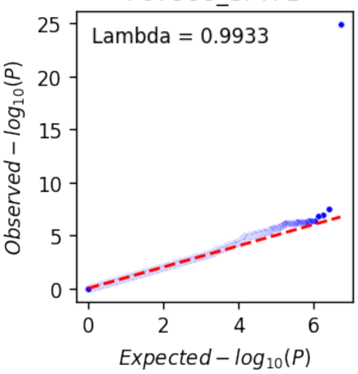

174
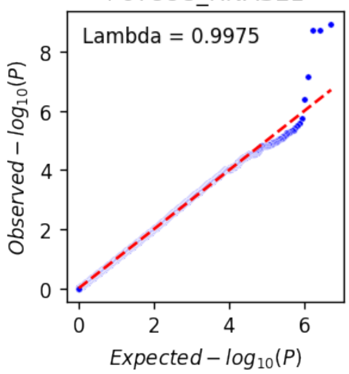

175
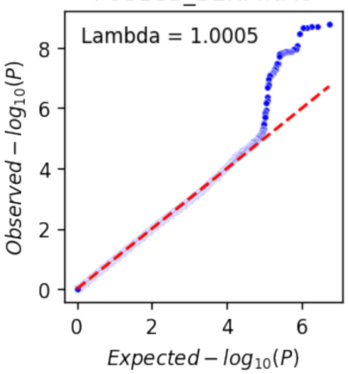

176
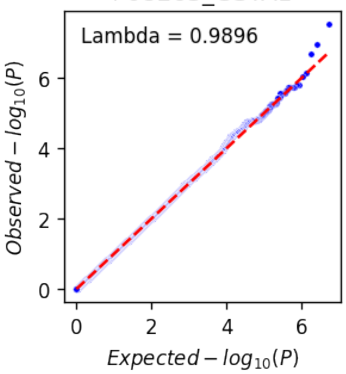

177
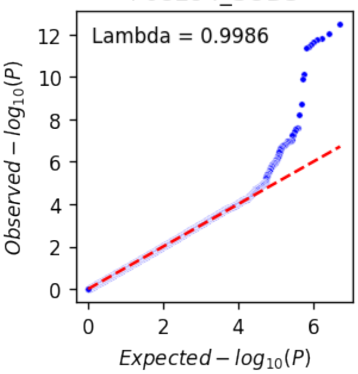

178
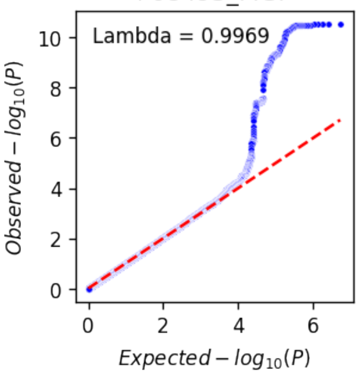

179
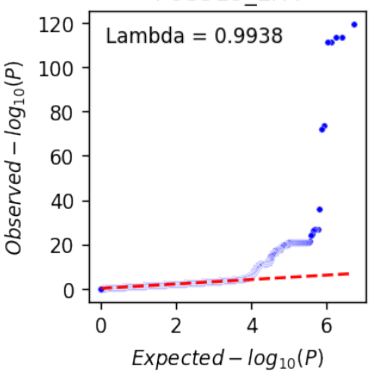

180
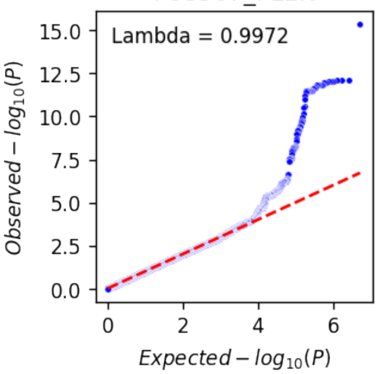

181

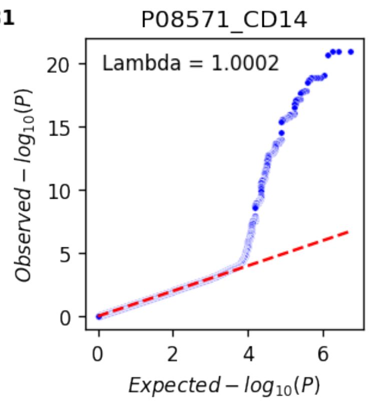

182

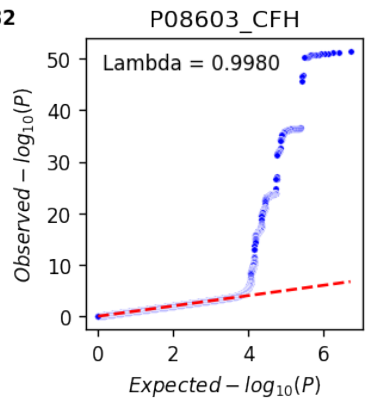

183

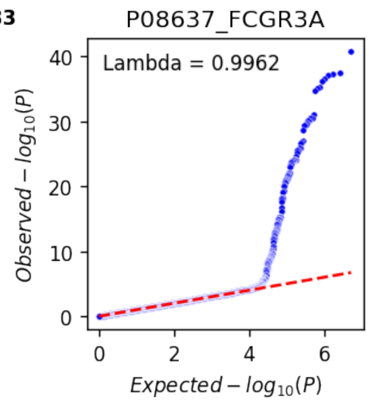

184

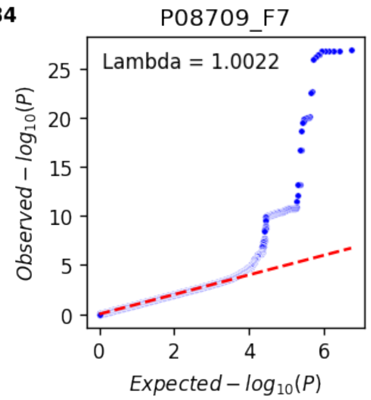

185

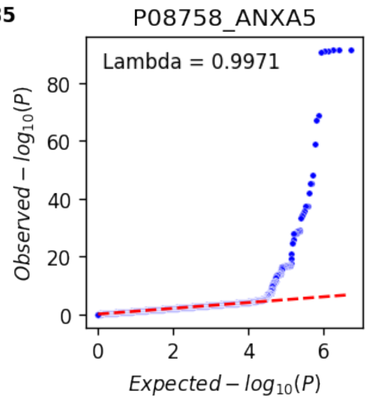

186

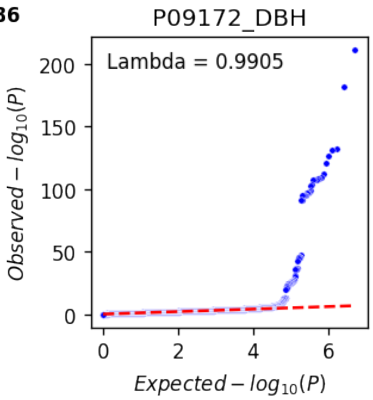

187

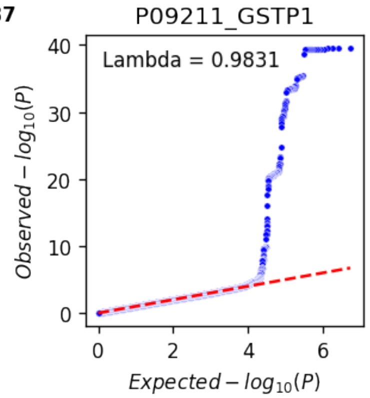

188

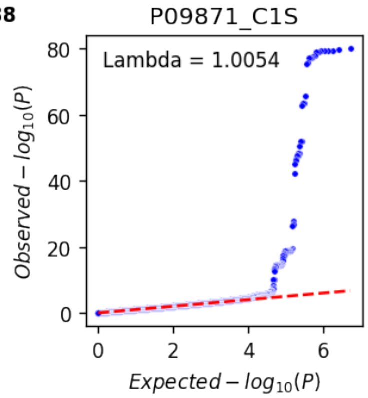

189

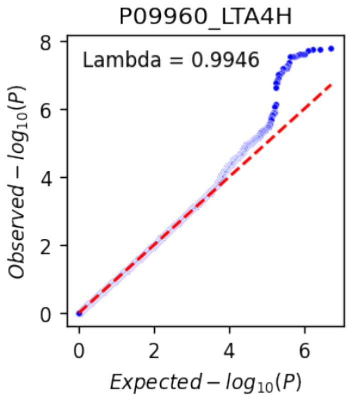

190

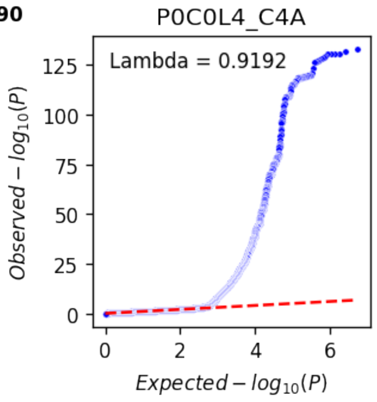

191

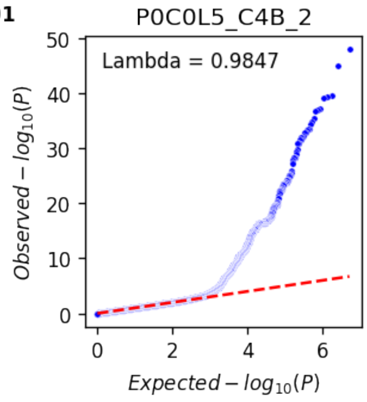

192

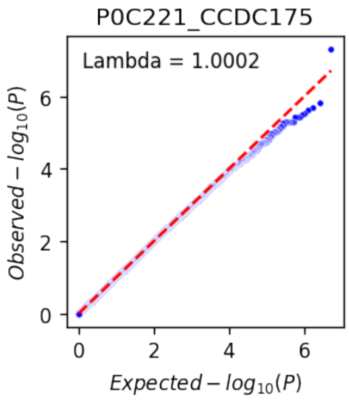

193
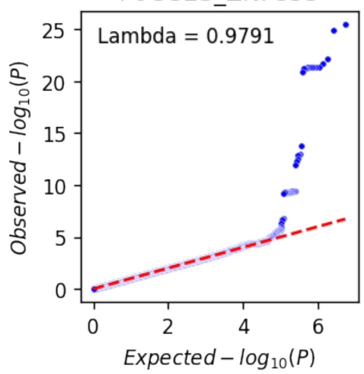

194
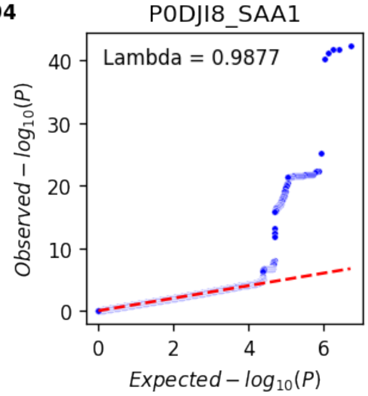

195
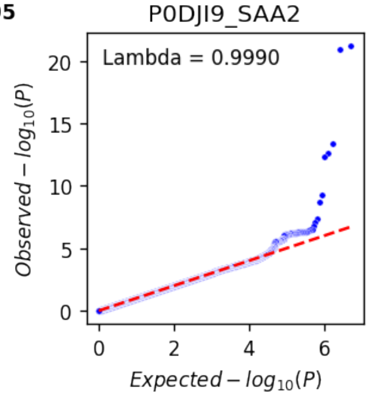

196
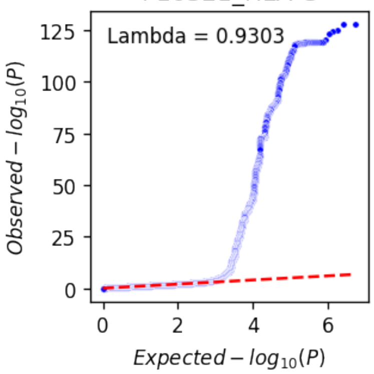

197
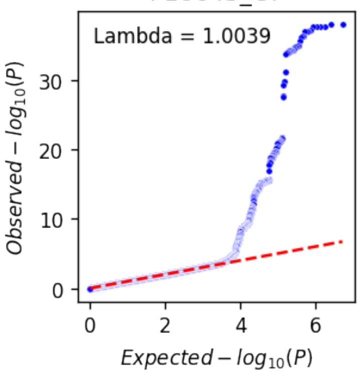

198
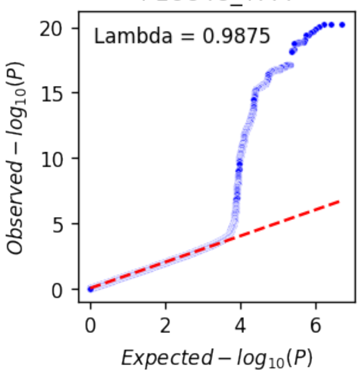

199
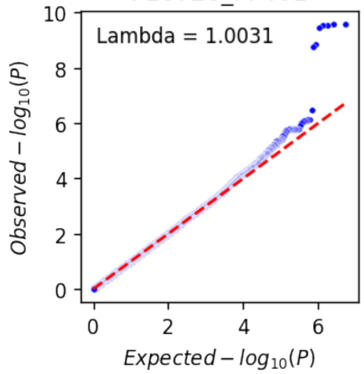

200
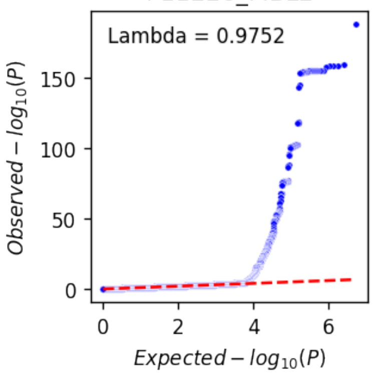

201
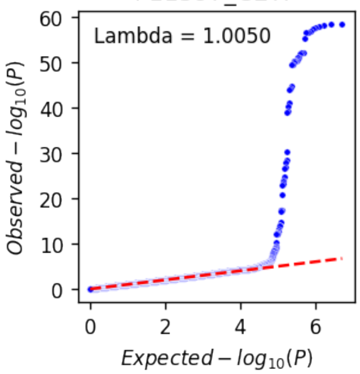

202
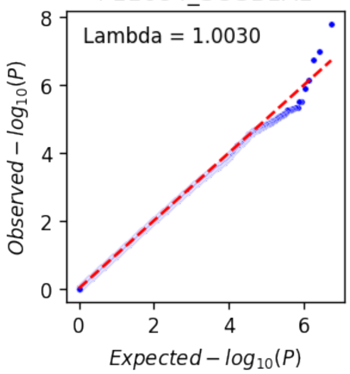

203
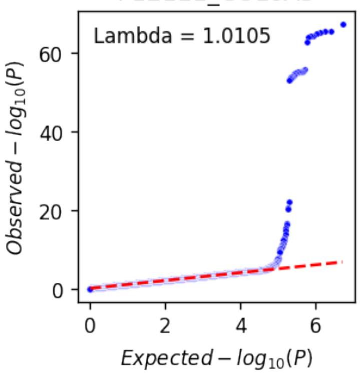

204
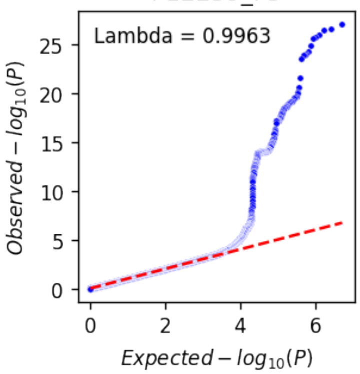

205

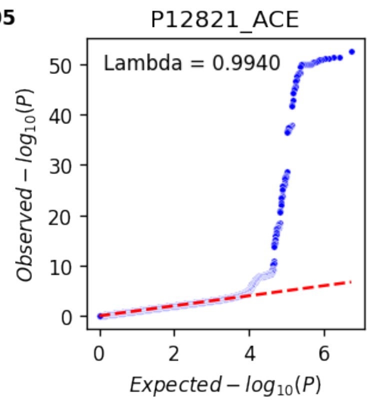

206

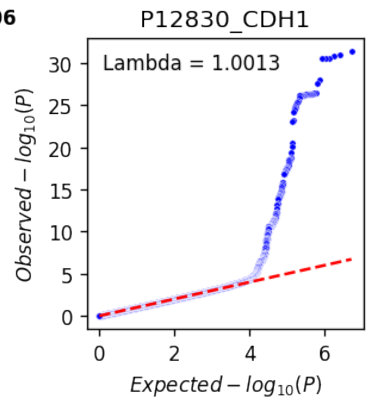

207

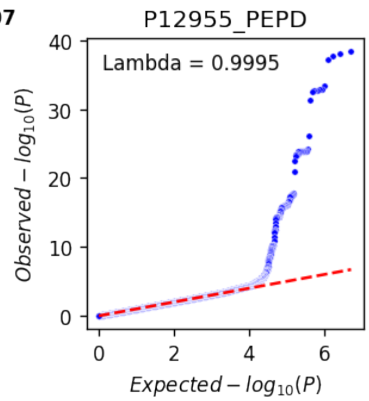

208

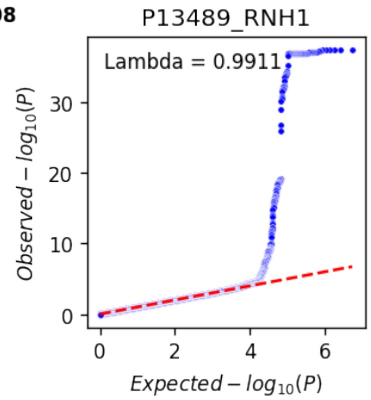

209

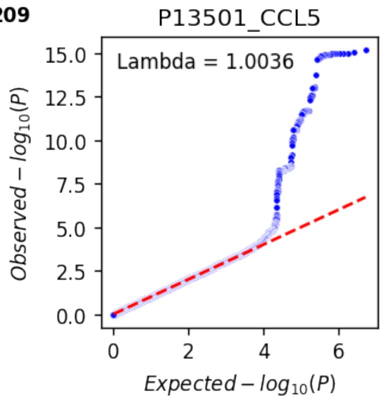

210

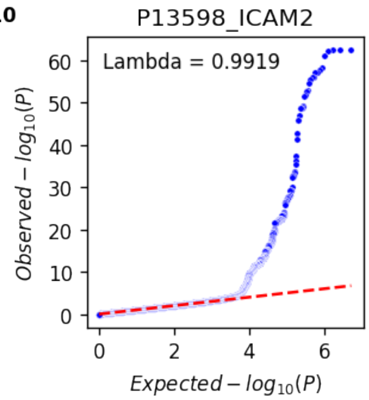

211

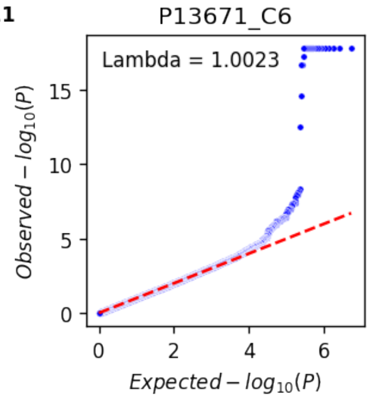

212

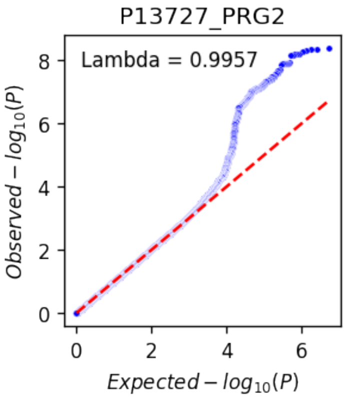

213

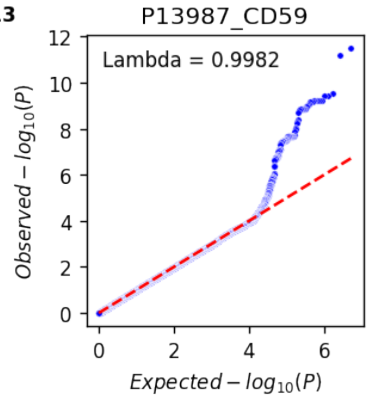

214

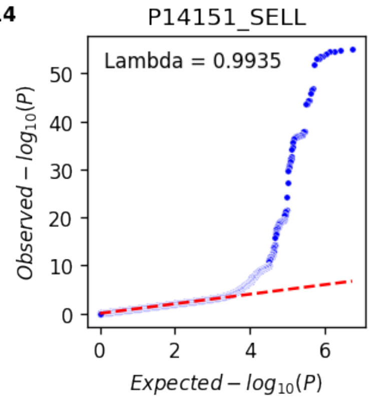

215

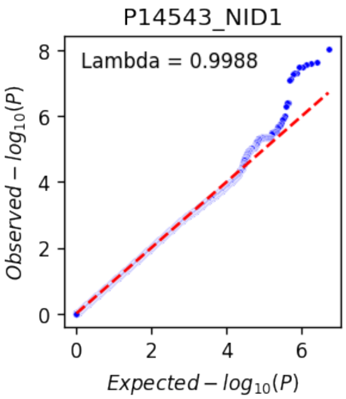

216

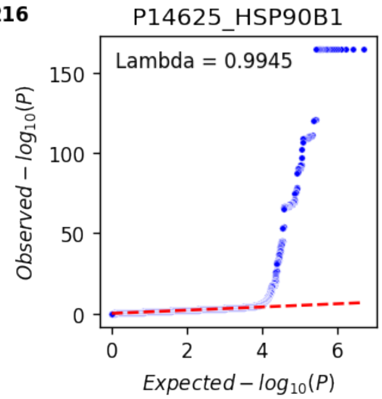

217

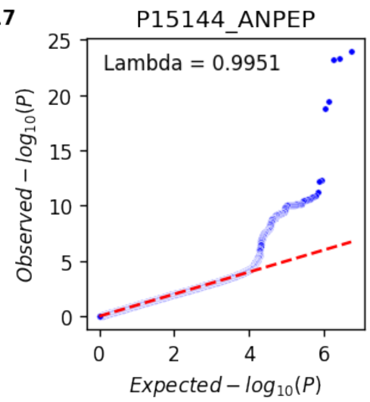

218

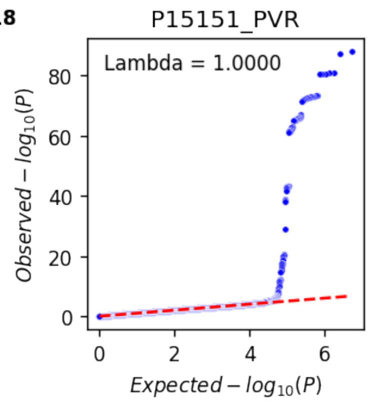

219

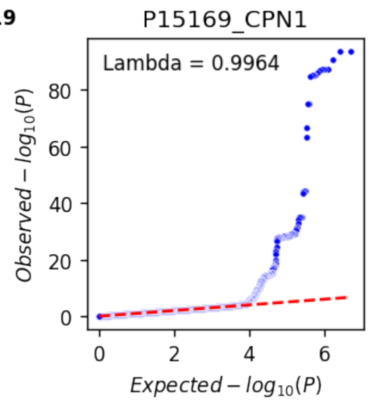

220

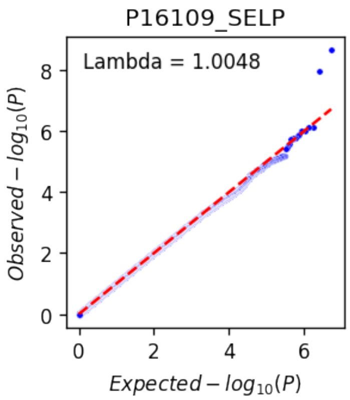

221

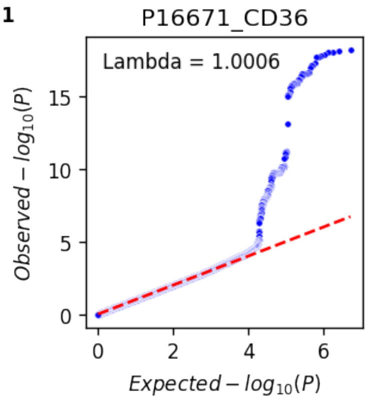

222

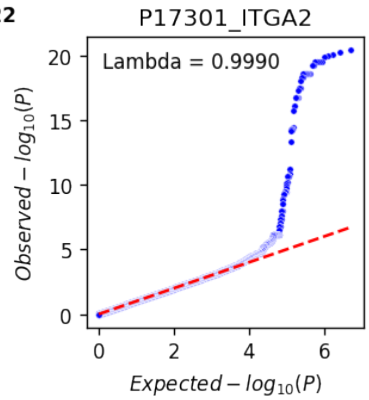

223

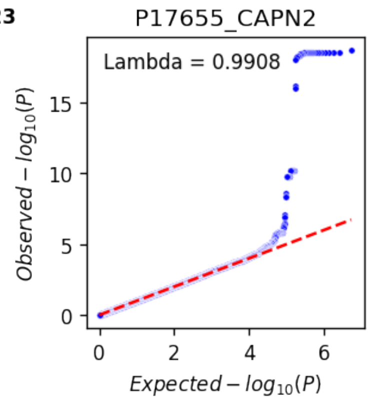

224

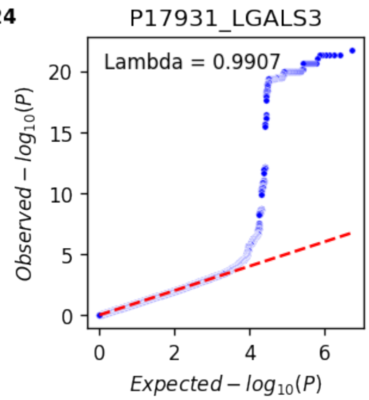

225

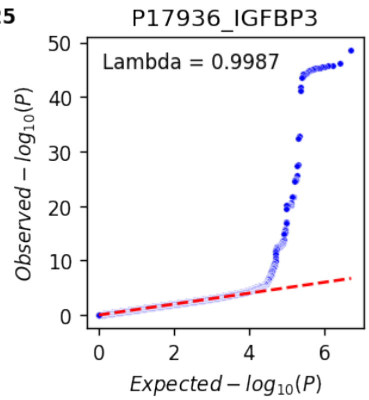

226

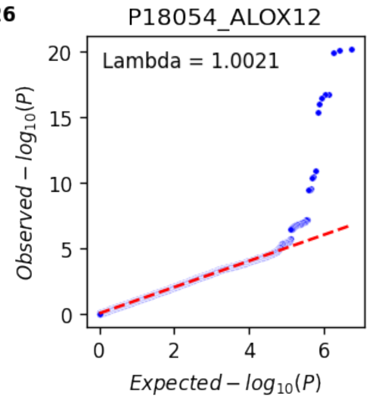

227

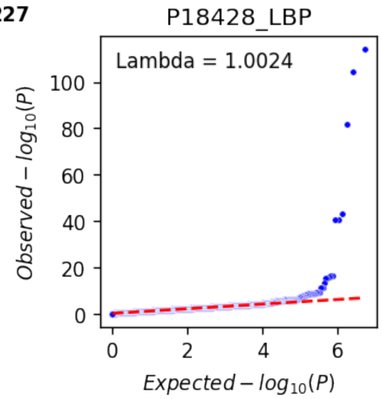

228

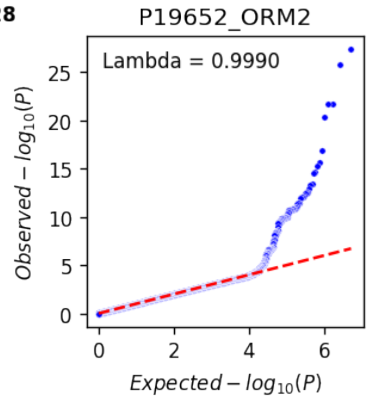

229

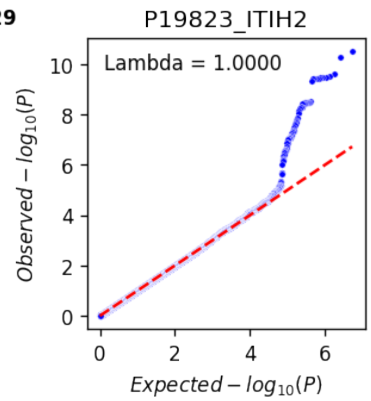

230

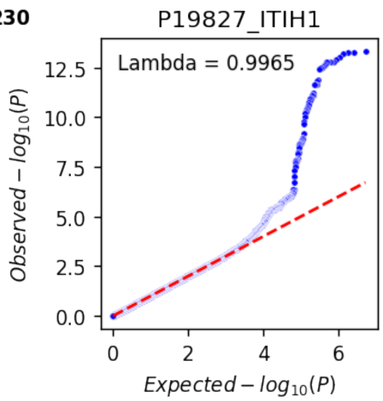

231

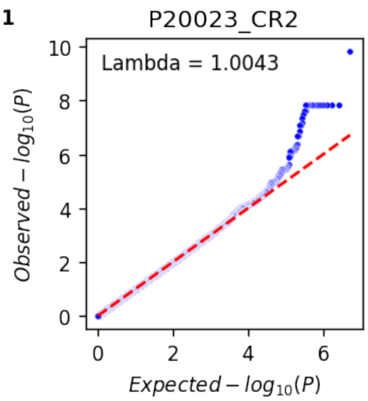

232

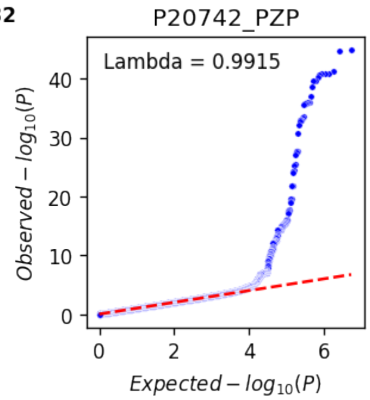

233

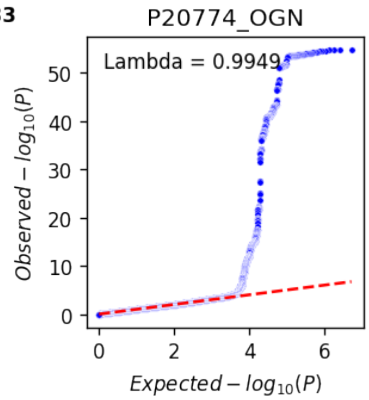

234

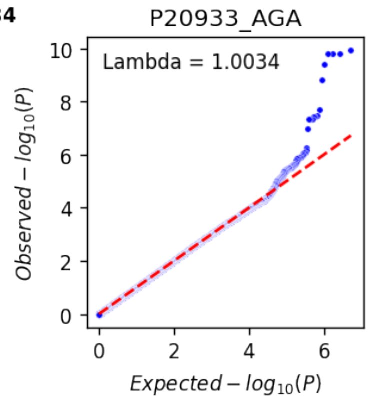

235

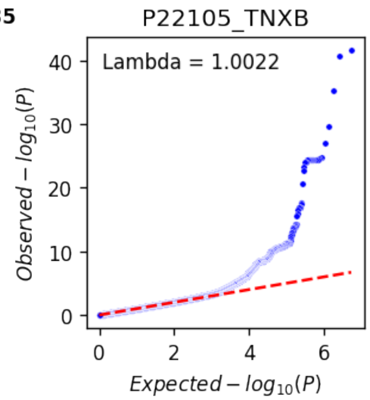

236

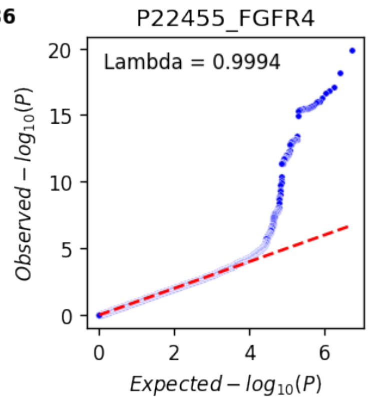

237

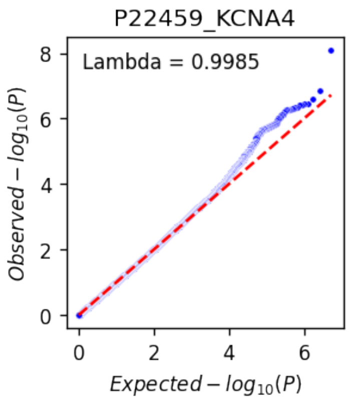

238

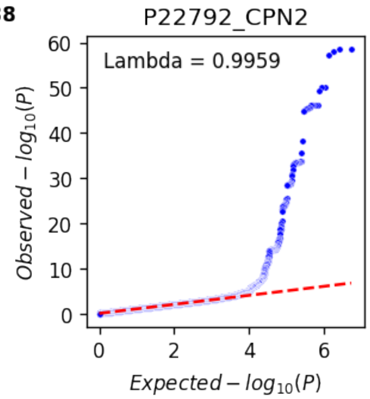

239

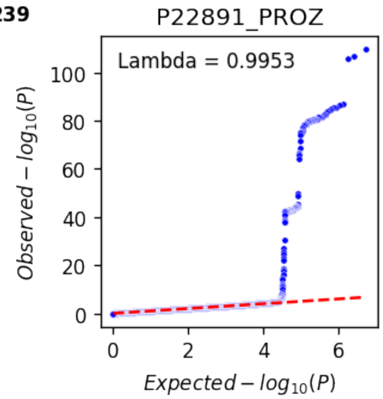

240

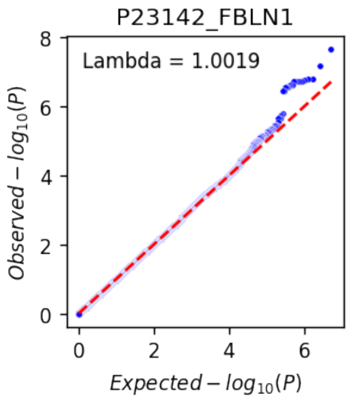

241 P23467_PTPRB

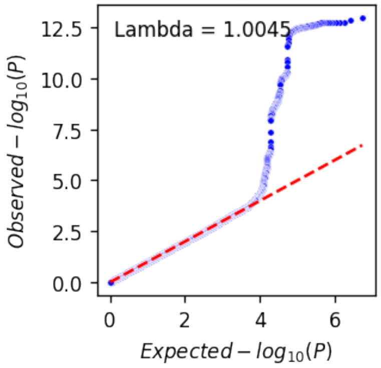

242 P24387_CRHBP

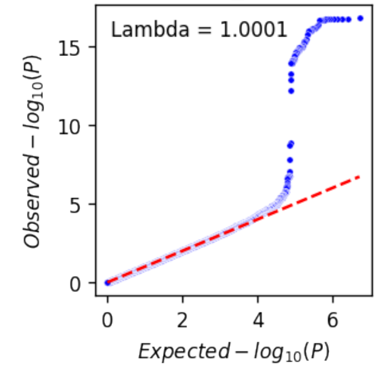

243 P24821_TNC

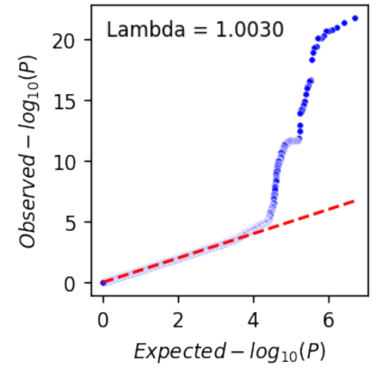

244 P25311_AZGP1

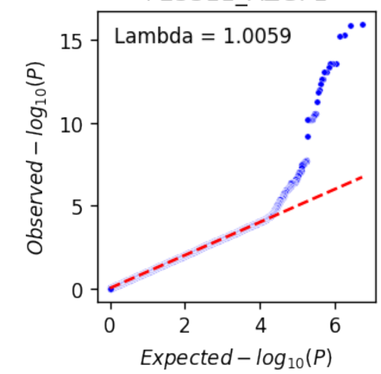

245 P26927_MST1

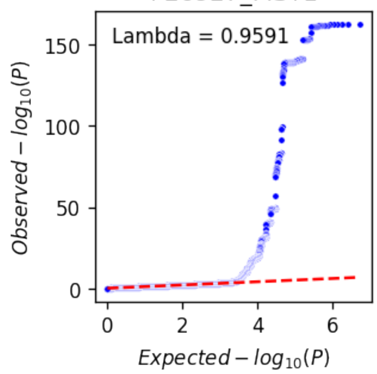

246 P27169_PON1

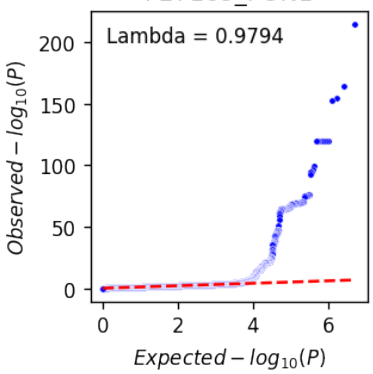

247 P27487_DPP4

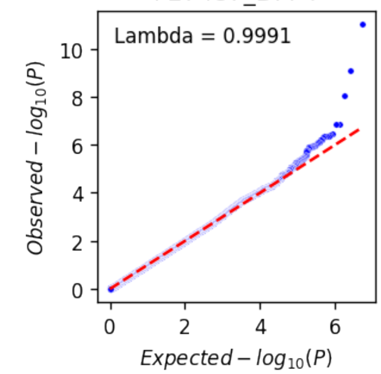

248 P27918_CFP

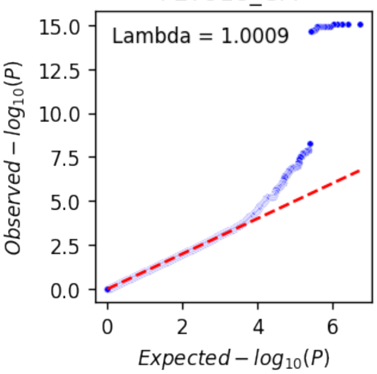

249 P28062_PSMB8

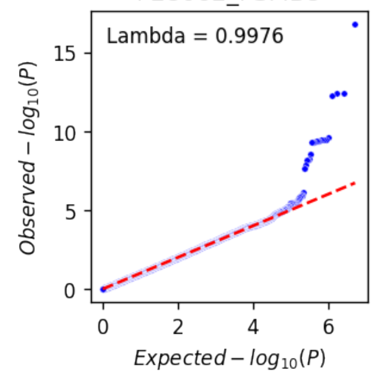

250 P28072_PSMB6

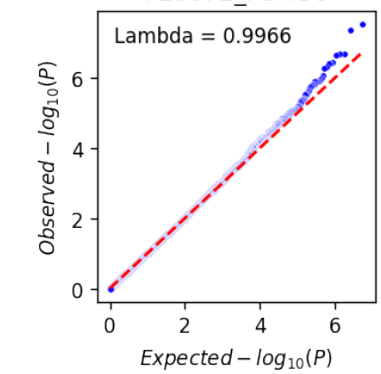

251 P28221_HTR1D

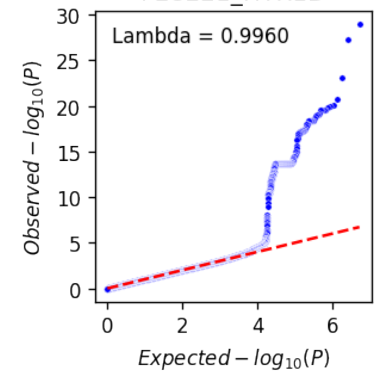

252 P28329_CHAT

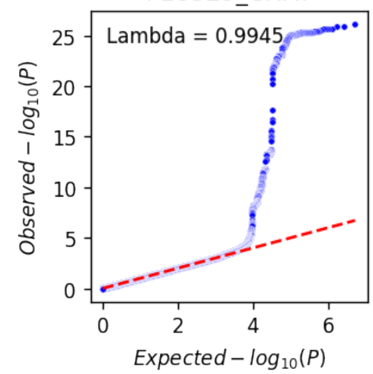

253

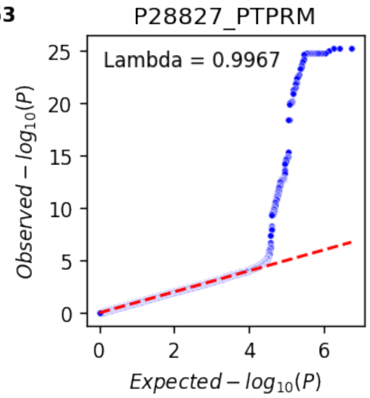

254

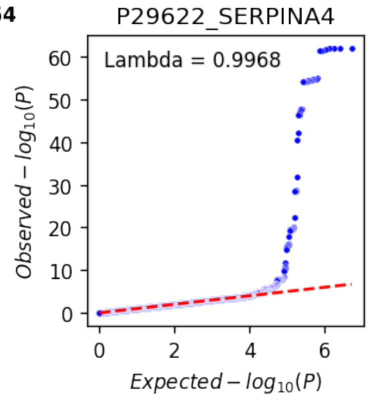

255

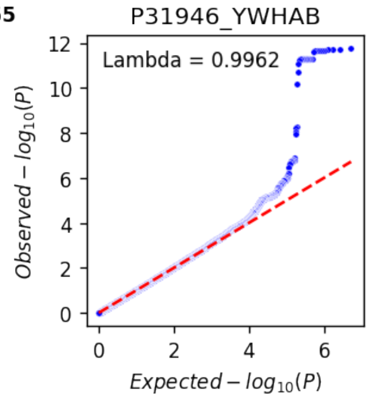

256

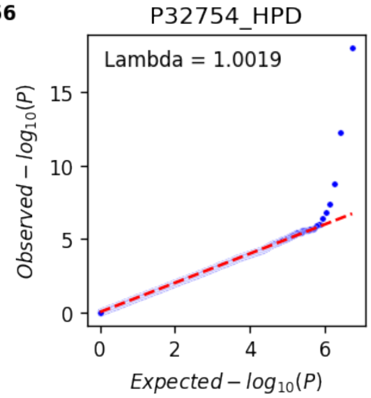

257

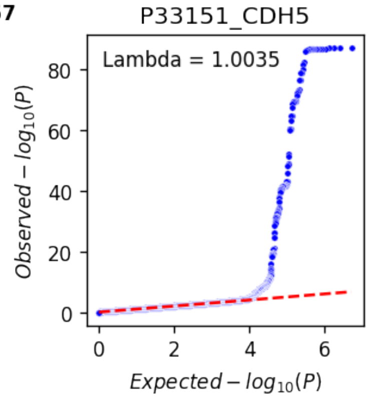

258

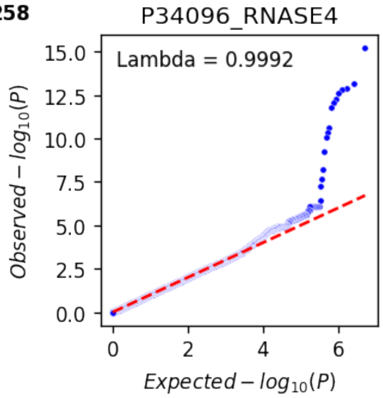

259

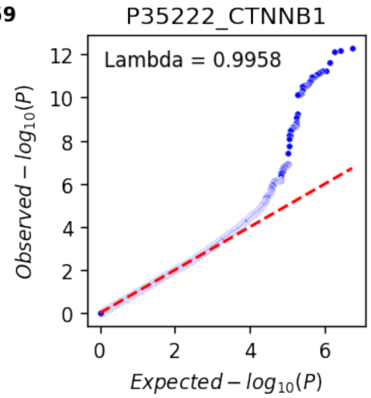

260

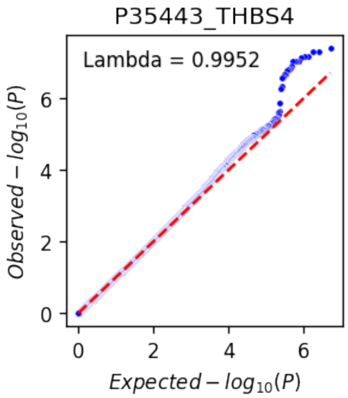

261

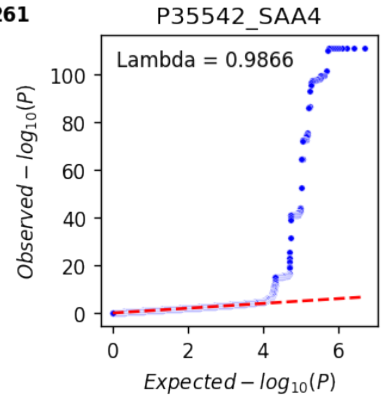

262

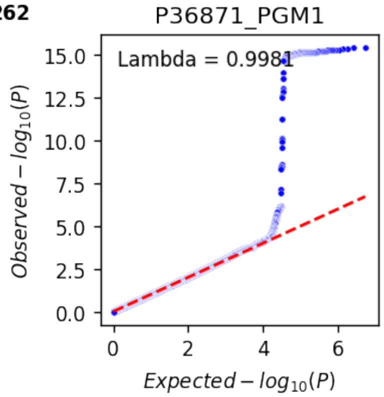

263

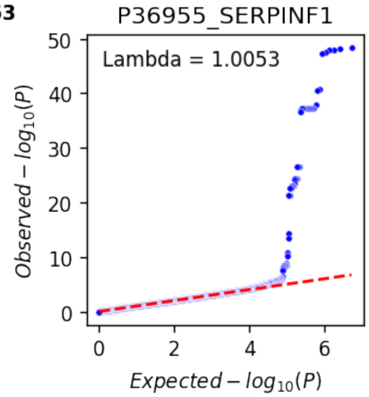

264

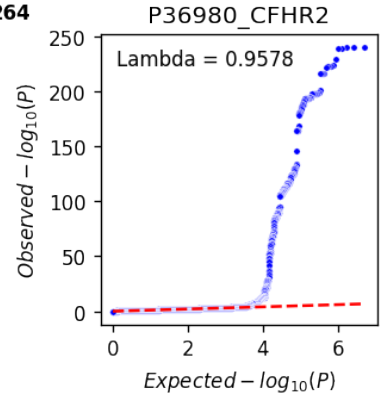

265 P37802_TAGLN2

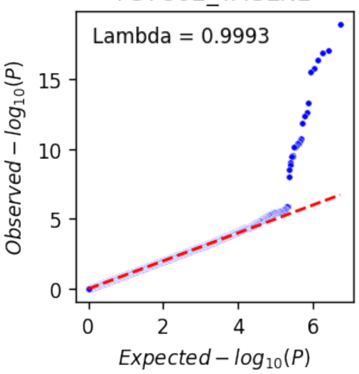

266 P39060_COL18A1

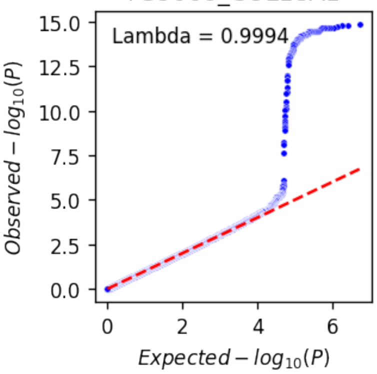

267 P41229_KDM5C

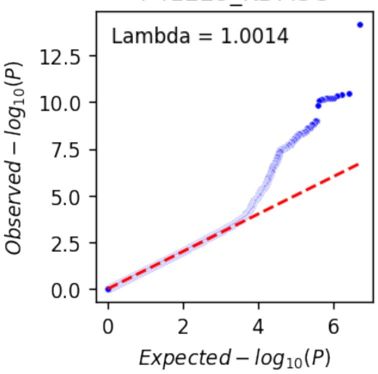

268 P43251_BTD

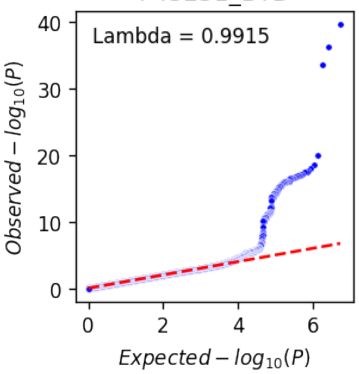

269 P43304_GPD2

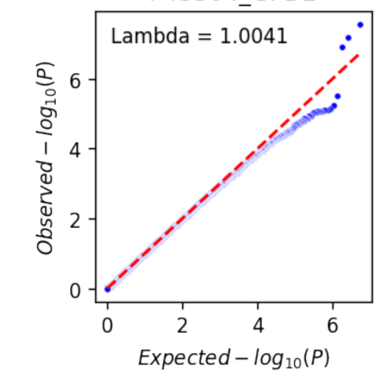

270 P48059_LIMS1

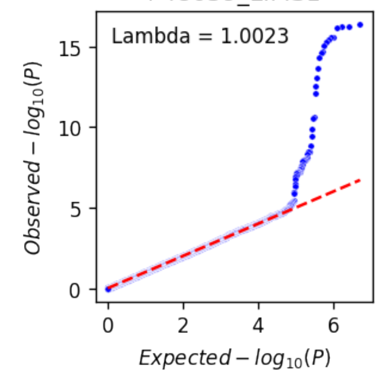

271 P48740_MASP1

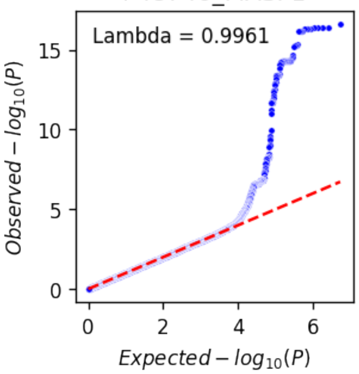

272 P49908_SELENOP

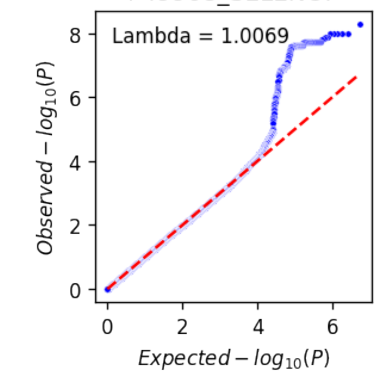

273 P49913_CAMP

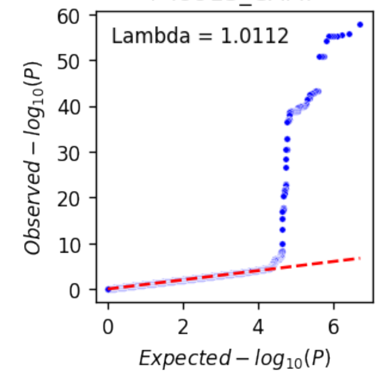

274 P51512_MMP16

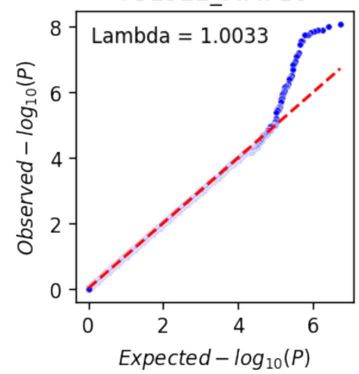

275 P53621_COPA

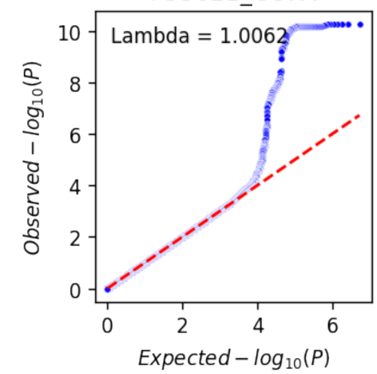

276 P54108_CRISP3

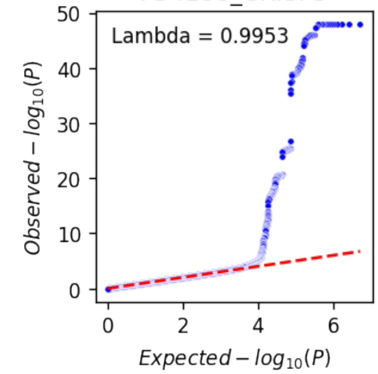

277

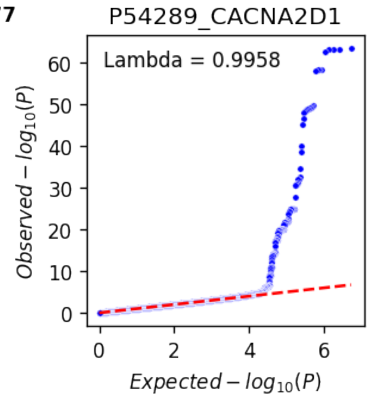

278

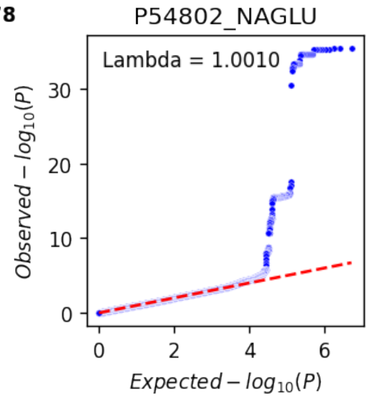

279

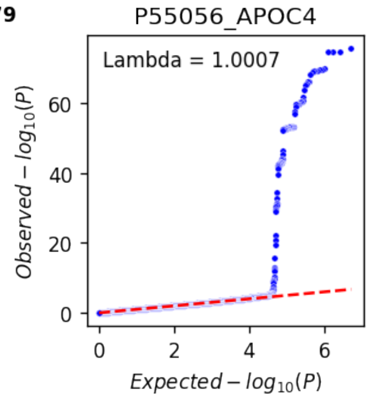

280

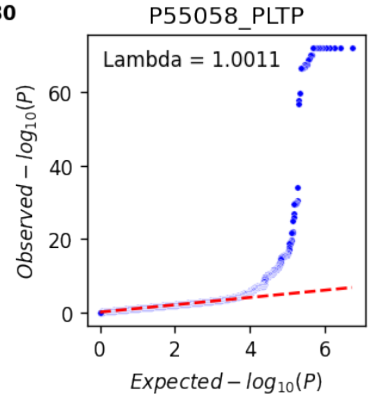

281

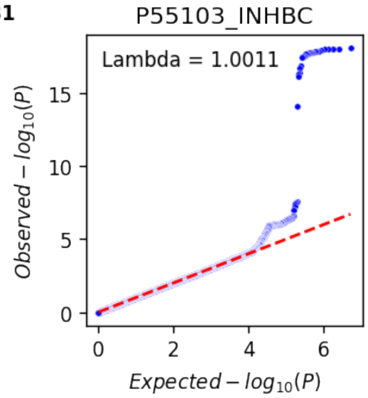

282

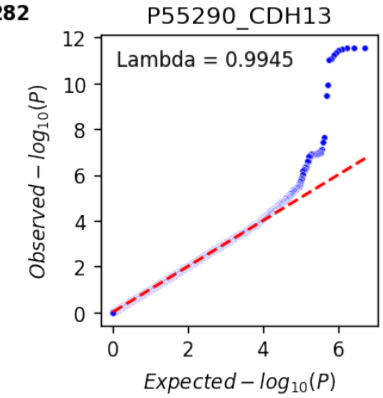

283

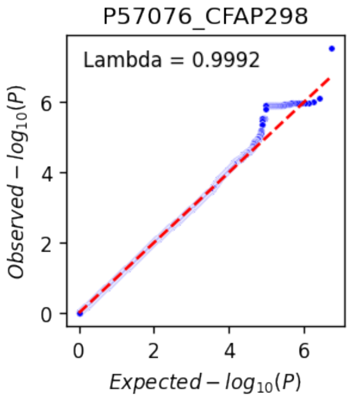

284

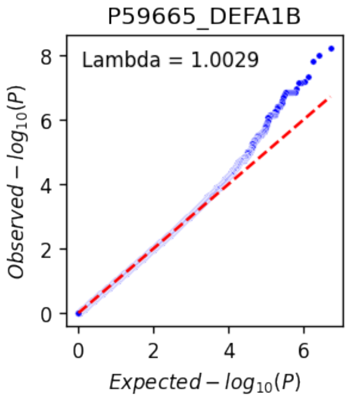

285

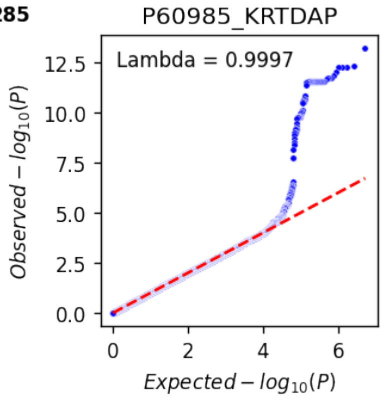

286

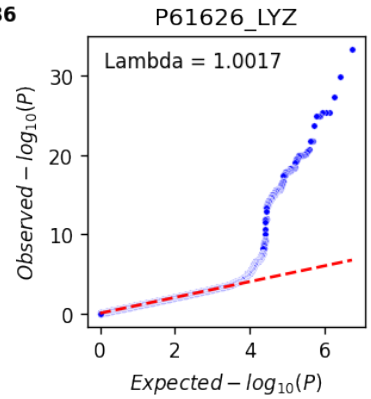

287

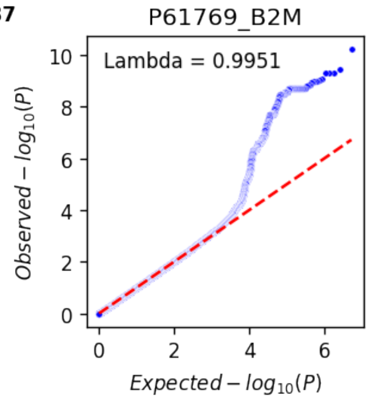

288

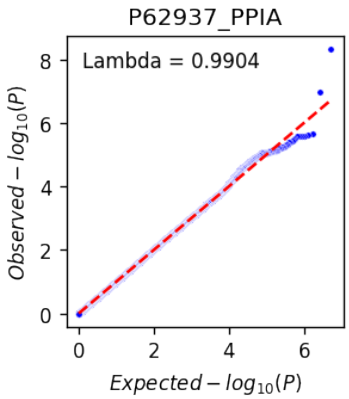

289 P62942_FKBP1A

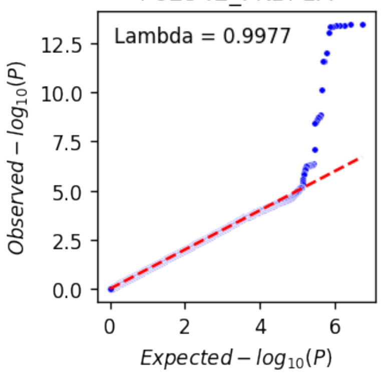

290 P68036_UBE2L3

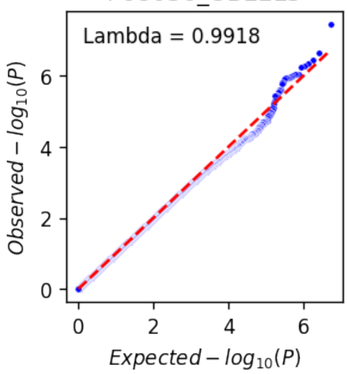

291 P68366_TUBA4A

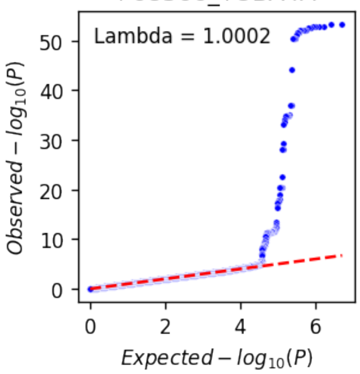

292 P78417_GSTO1

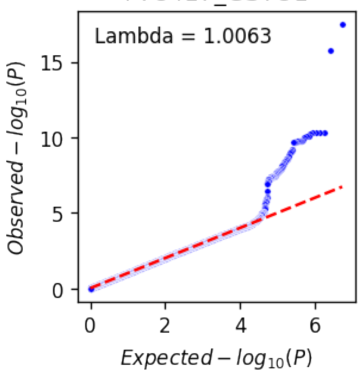

293 P78563_ADARB1

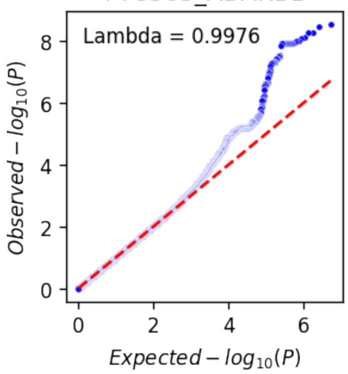

294 P82930_MRPS34

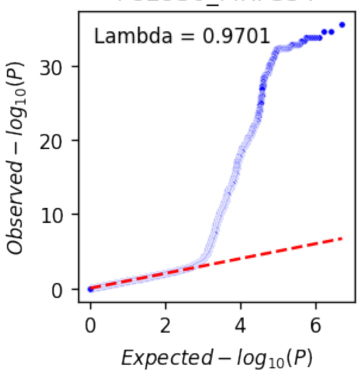

295 Q01459_CTBS

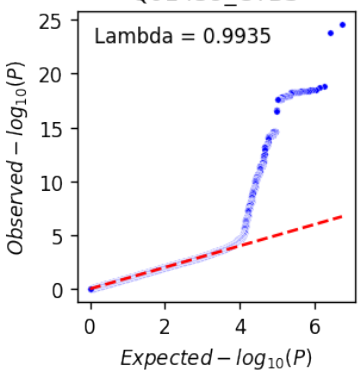

296 Q01813_PFKP

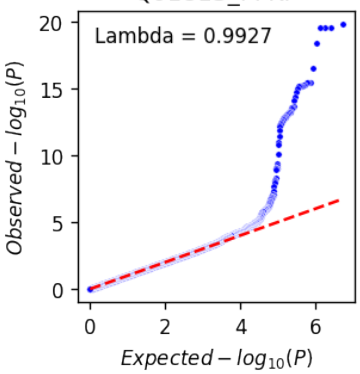

297 Q02985_CFHR3

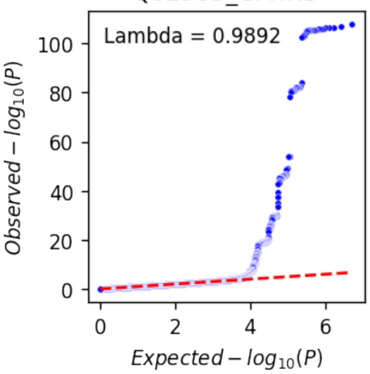

298 Q03591_CFHR1

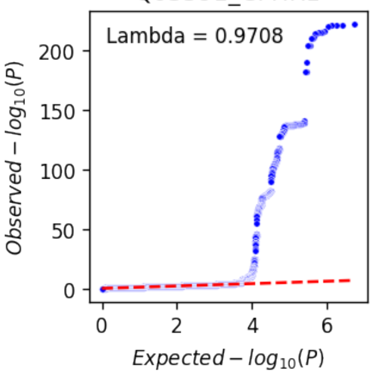

299 Q04756_HGFAC

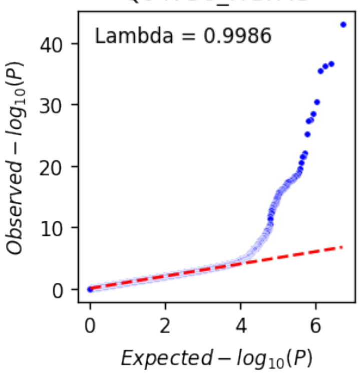

300 Q06033_ITIH3

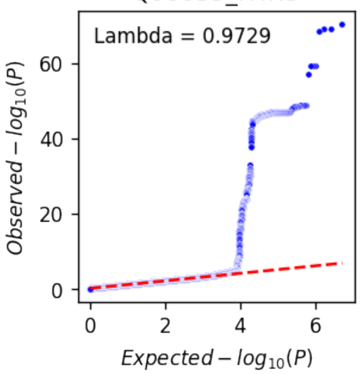

301

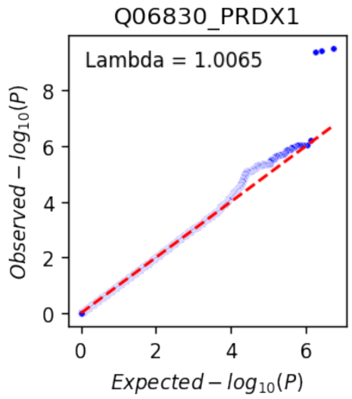

302

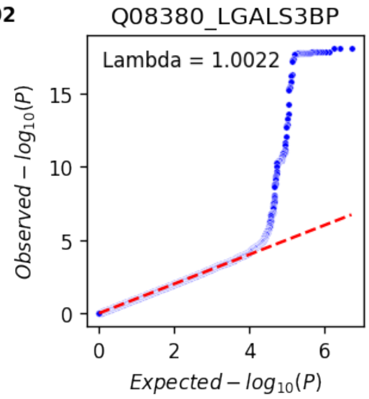

303

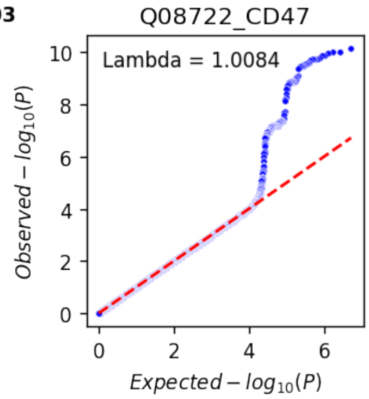

304

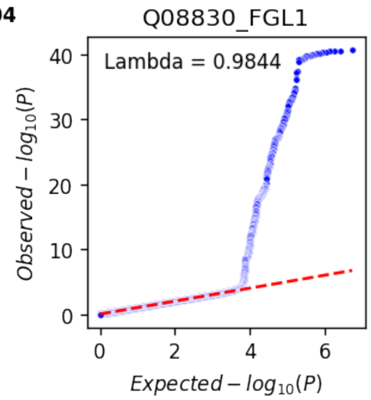

305

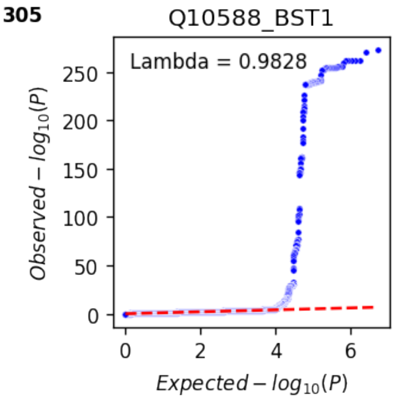

306

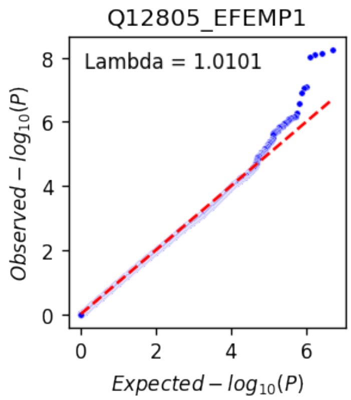

307

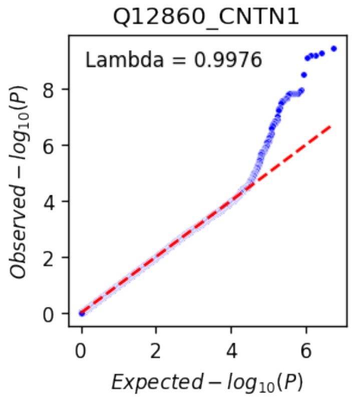

308

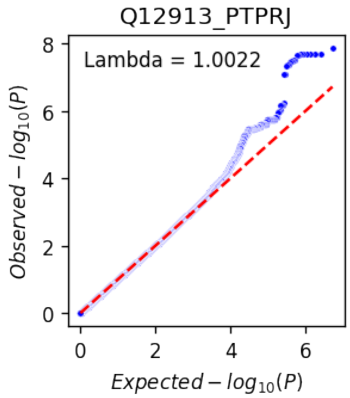

309

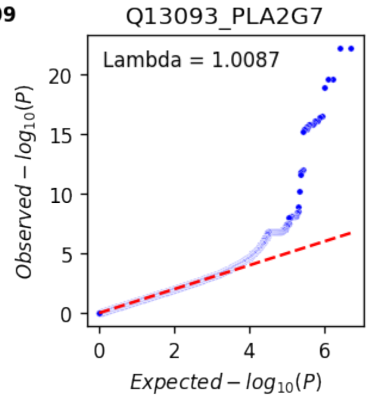

310

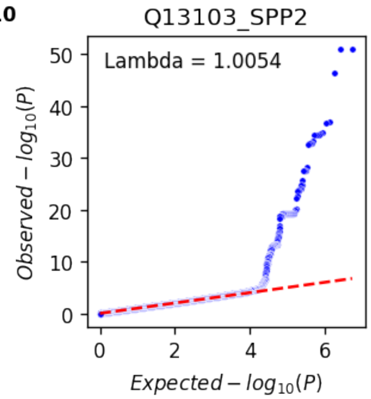

311

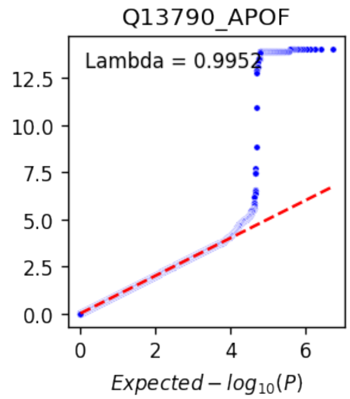

312

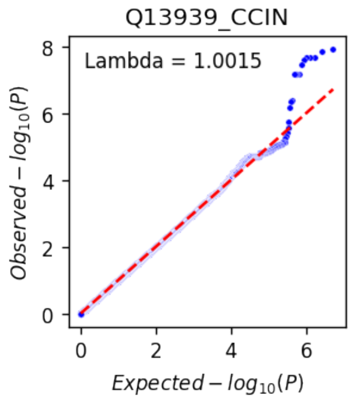

313 Q14515_SPARCL1

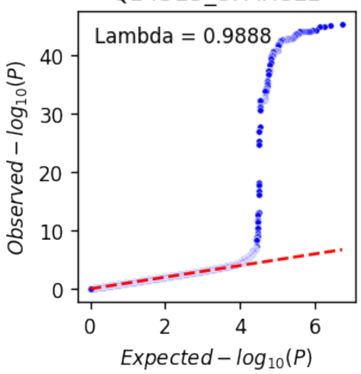

314 Q14520_HABP2

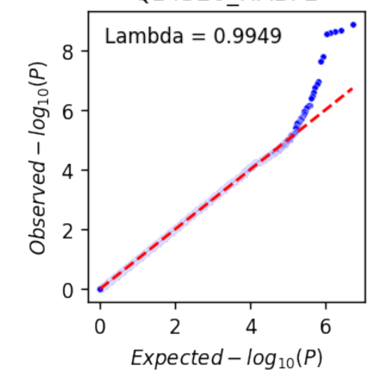

315 Q14623_IHH

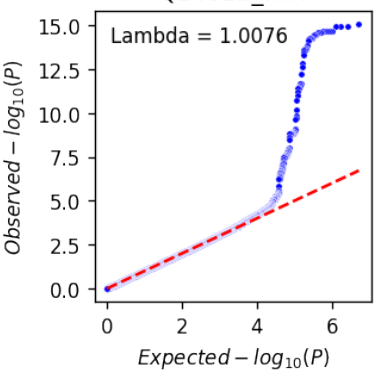

316 Q14624_ITIH4

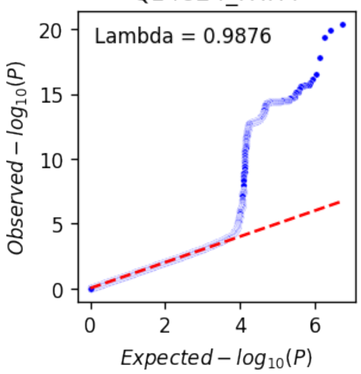

317 Q15063_POSTN

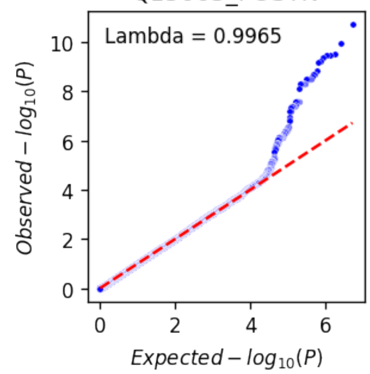

318 Q15113_PCOLCE

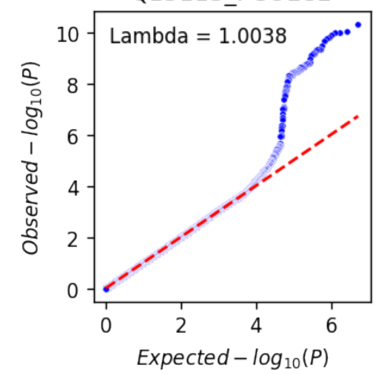

319 Q15166_PON3

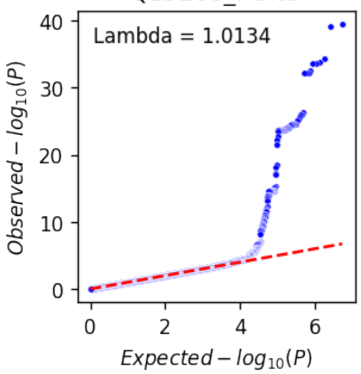

320 Q15223_NECTIN1

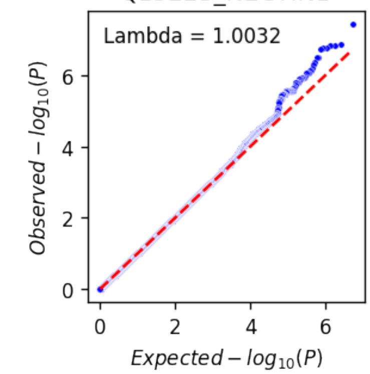

321 Q15404_RSU1

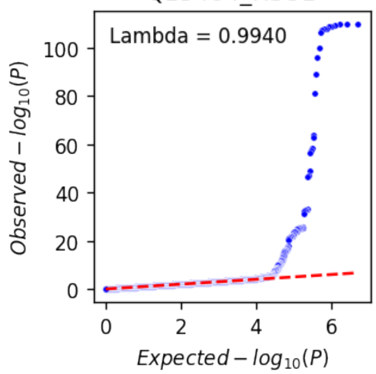

322 Q15465_SHH

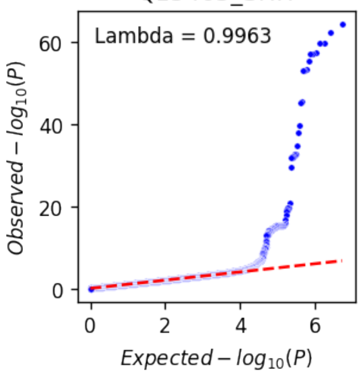

323 Q15485_FCN2

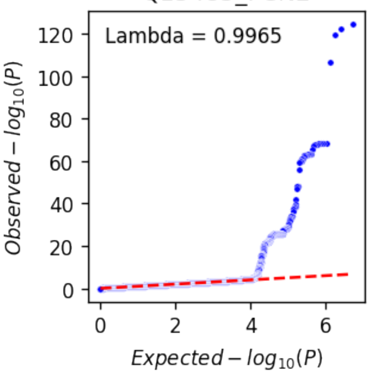

324 Q15517_CDSN

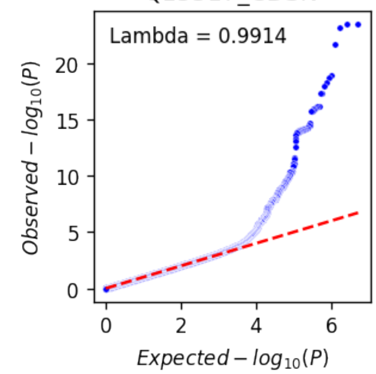

325

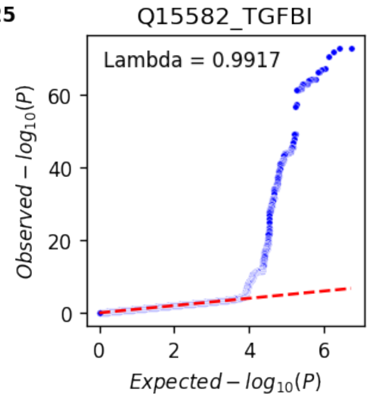

326

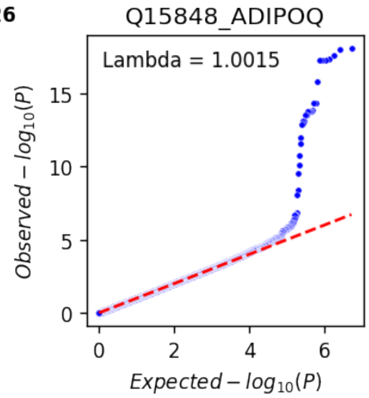

327

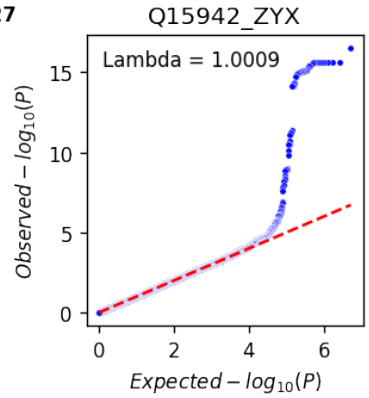

328

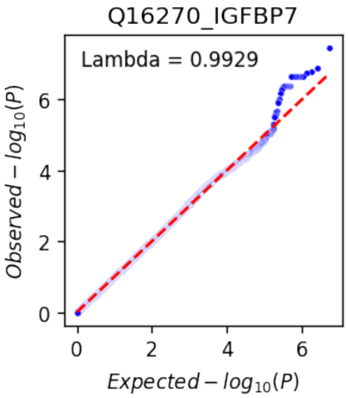

329

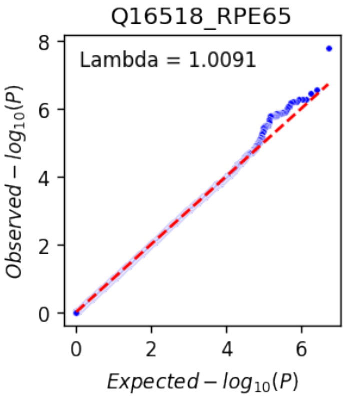

330

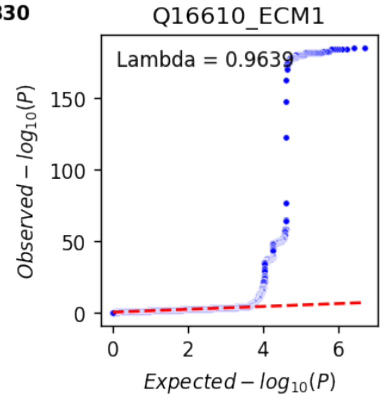

331

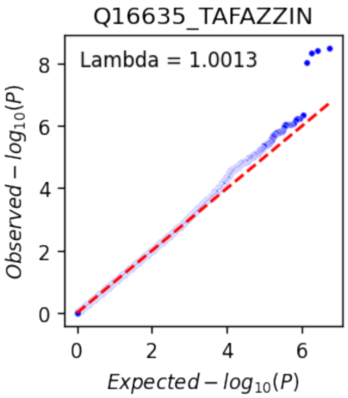

332

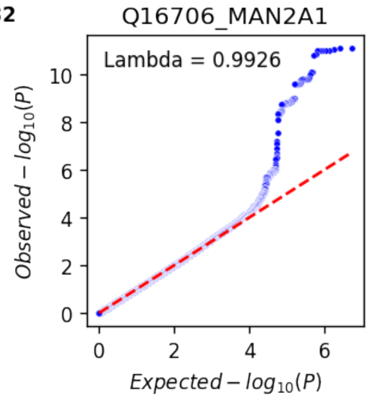

333

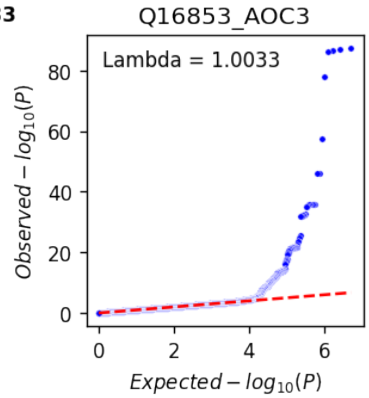

334

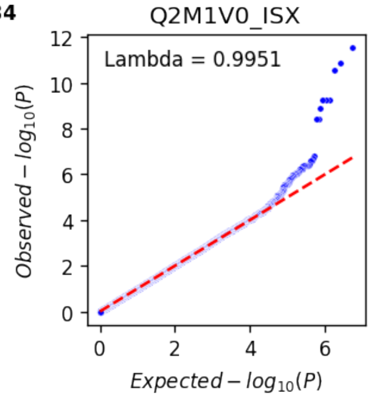

335

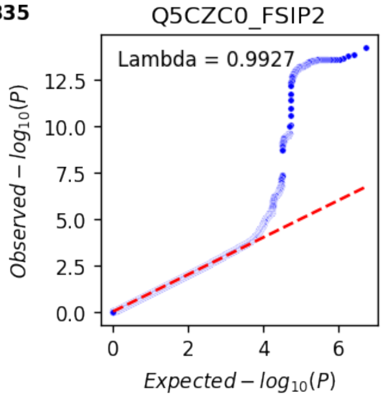

336

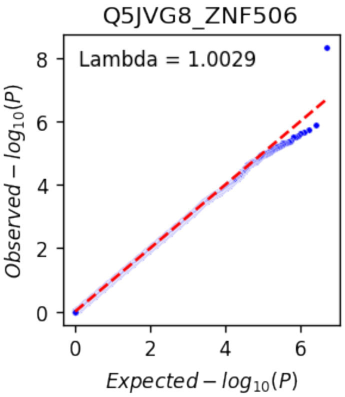

337

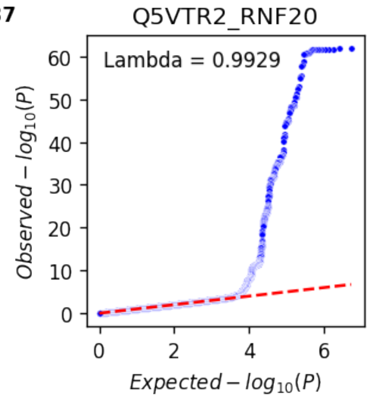

338

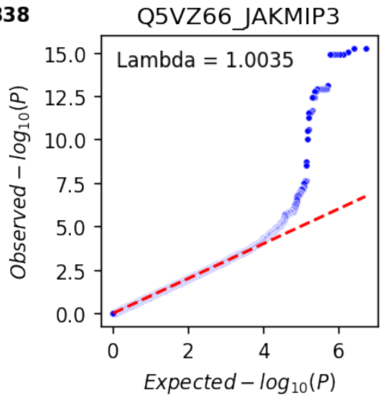

339

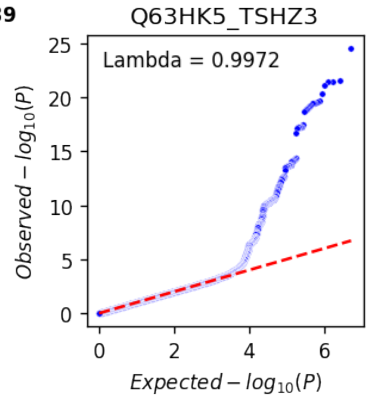

340

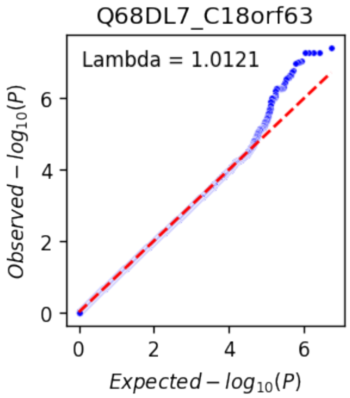

341

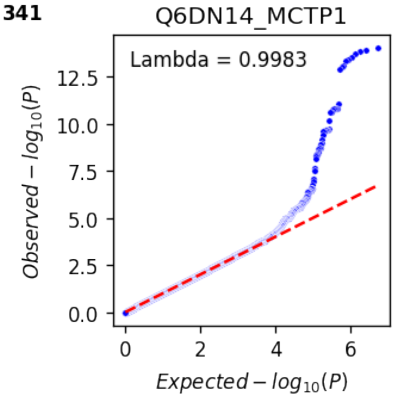

342

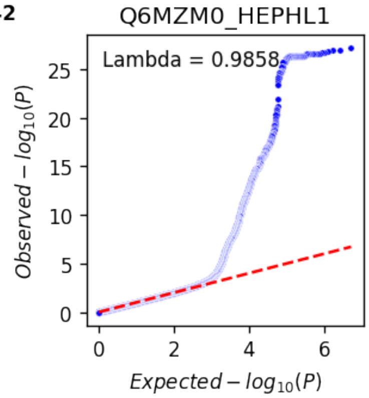

343

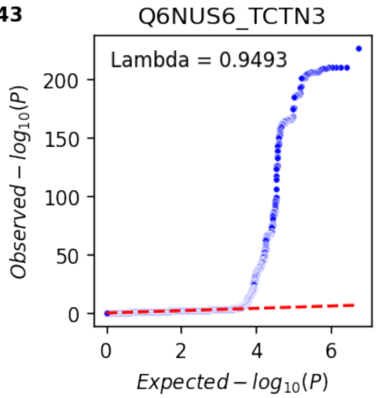

344

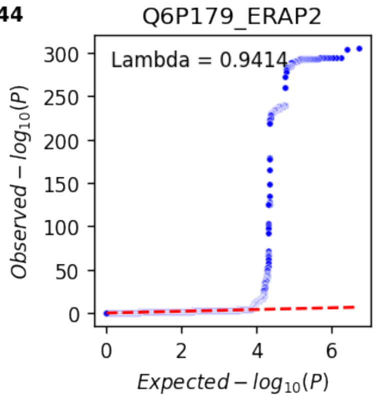

345

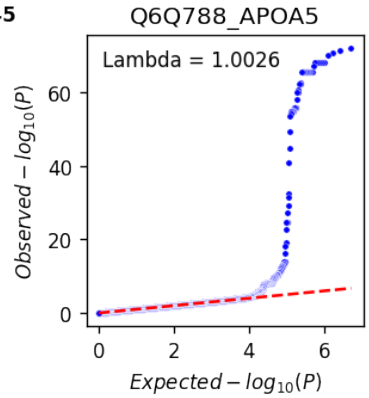

346

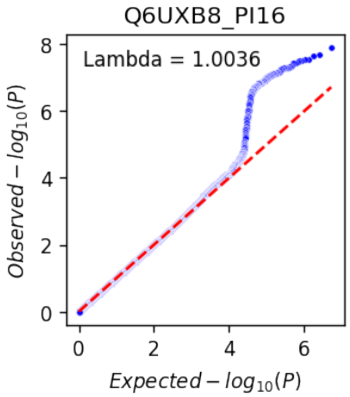

347

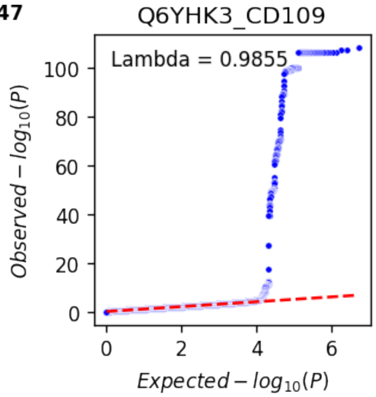

348

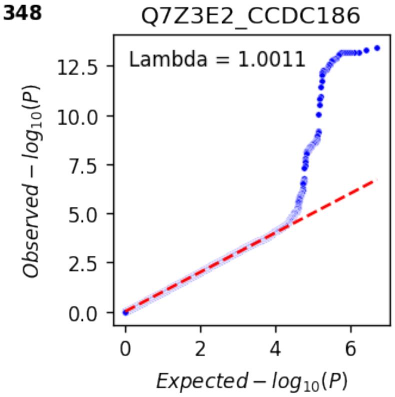

349

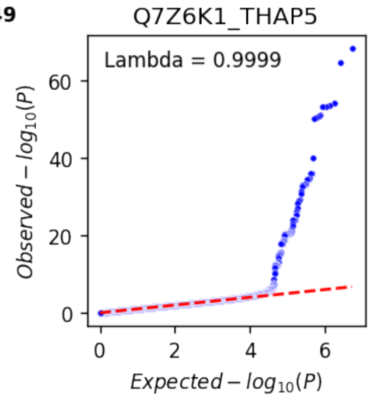

350

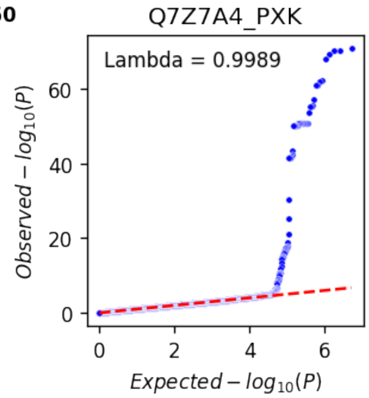

351

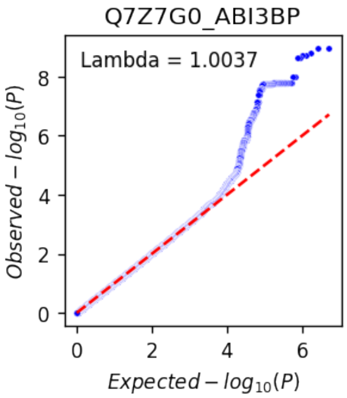

352

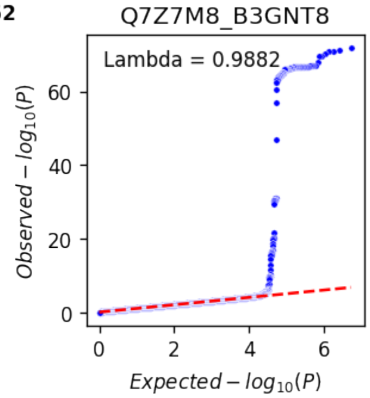

353

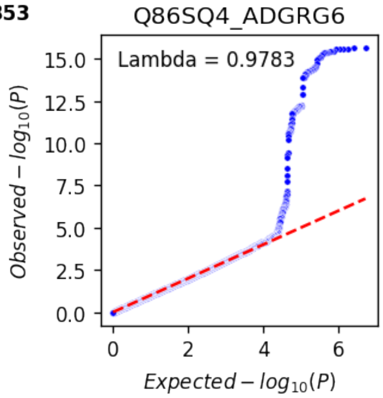

354

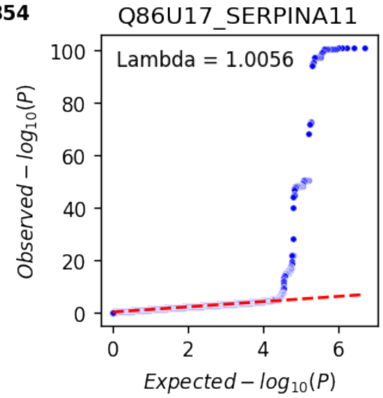

355

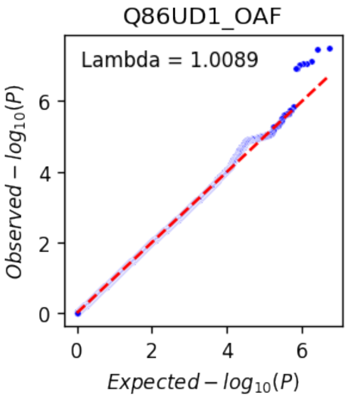

356

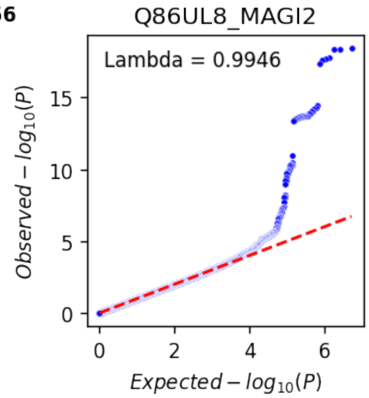

357

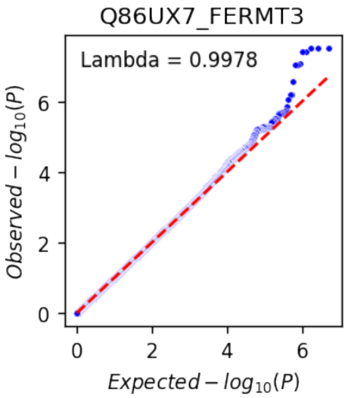

358

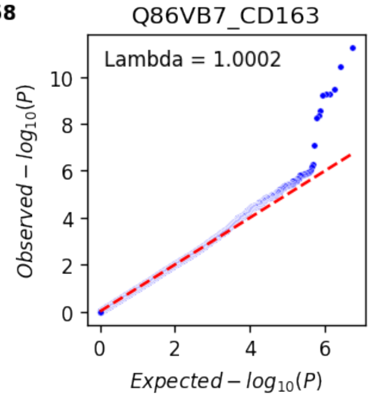

359

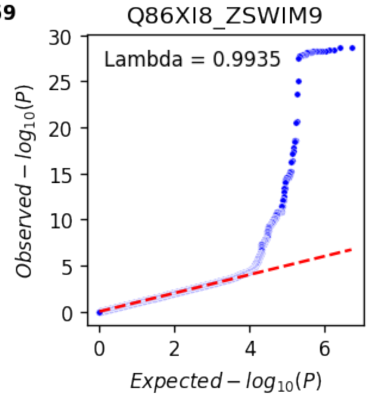

360

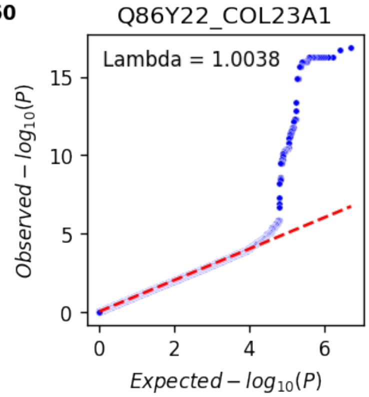

361

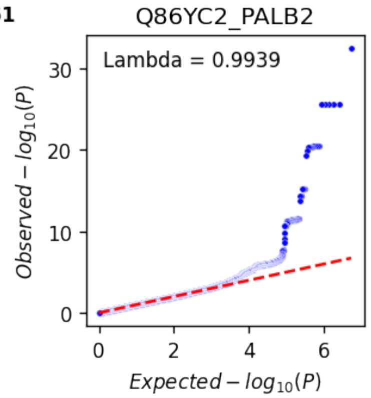

362

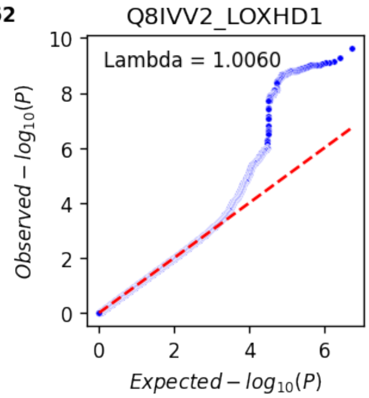

363

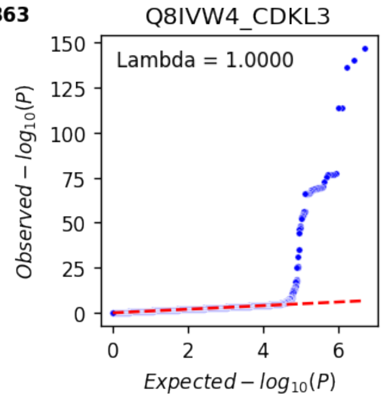

364

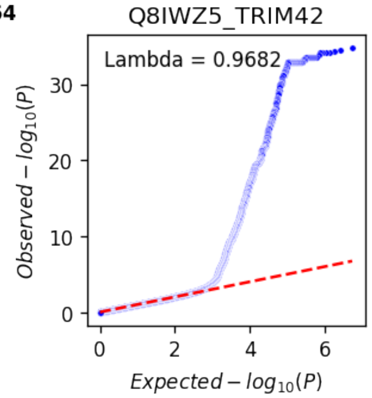

365

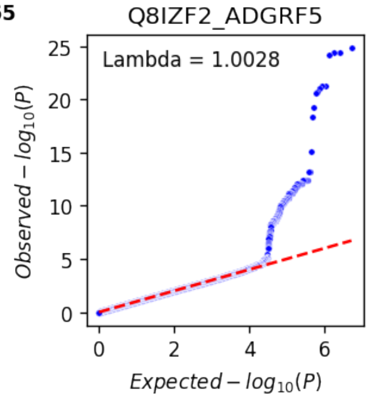

366

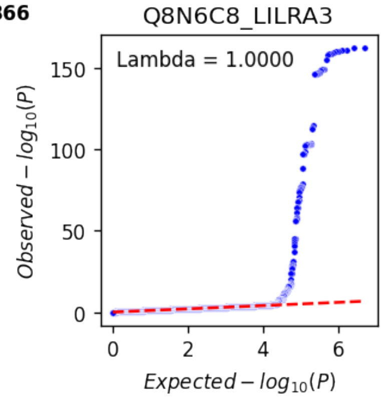

367

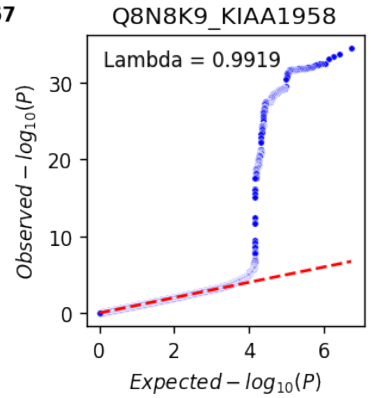

368

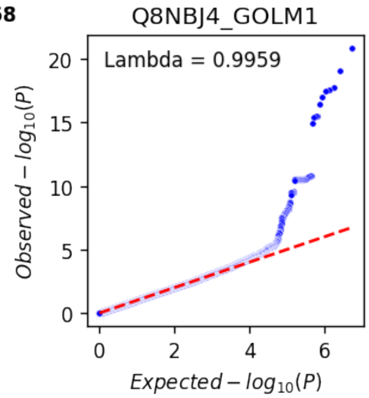

369

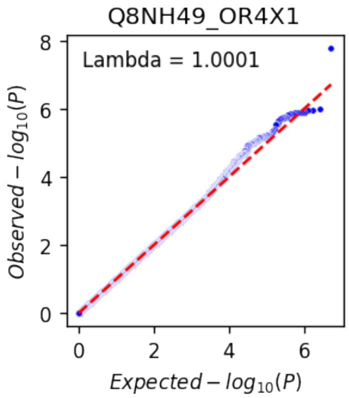

370

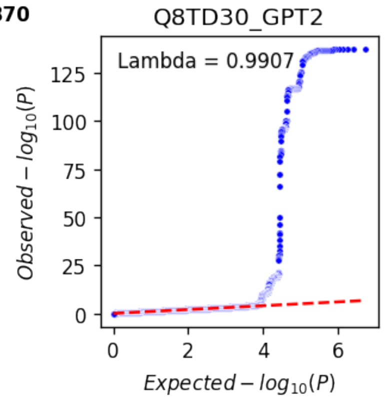

371

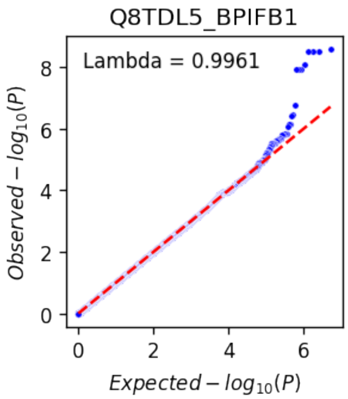

372

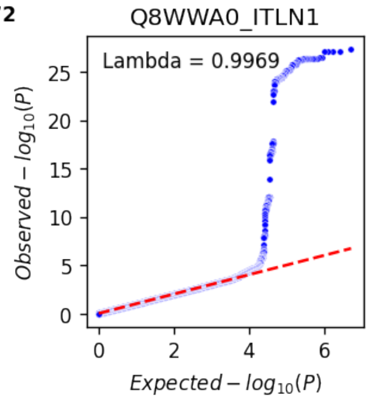

373
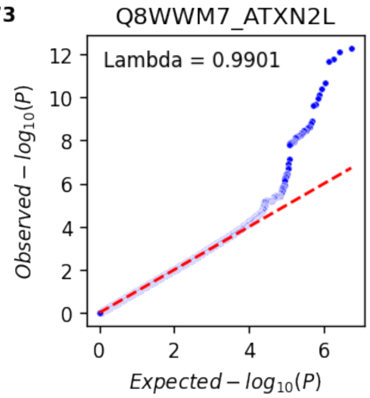

374
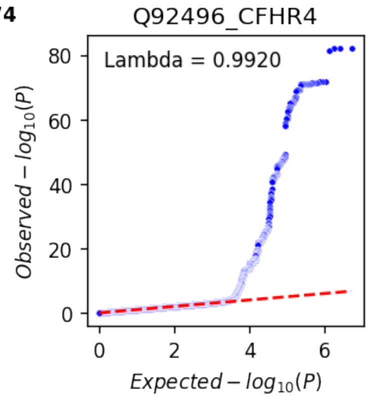

375
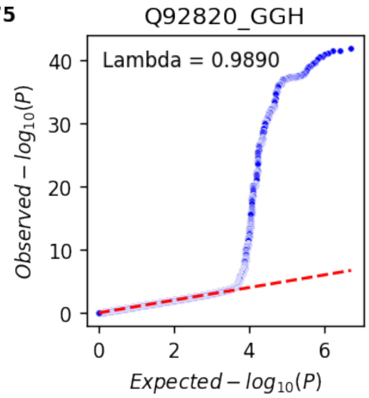

376
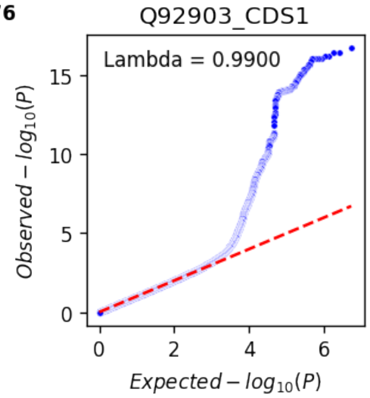

377
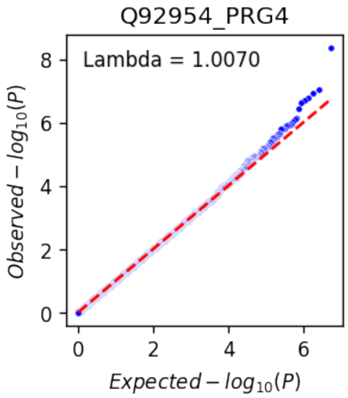

378
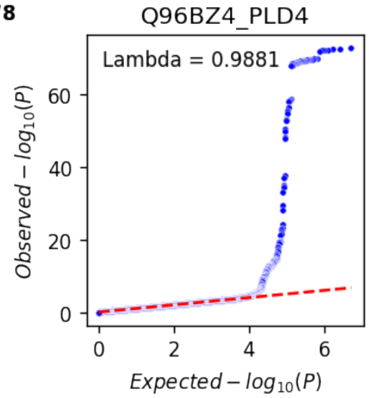

379
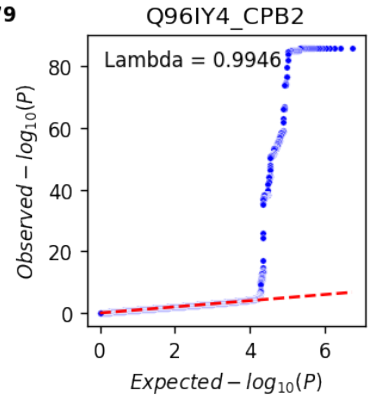

380
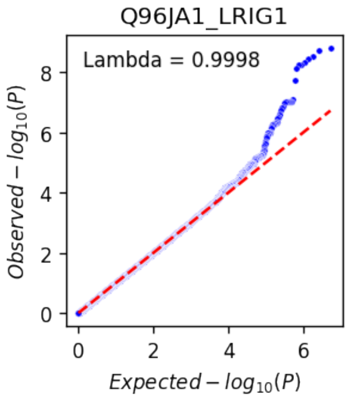

381
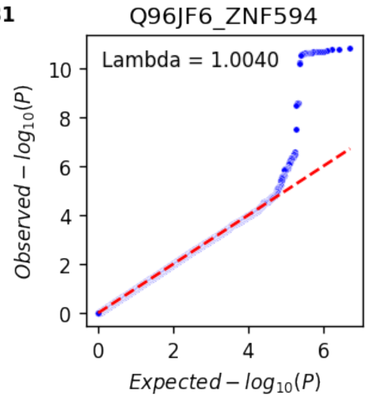

382
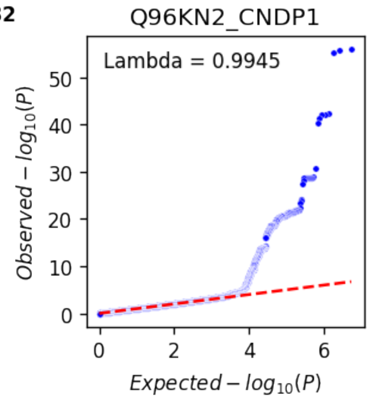

383
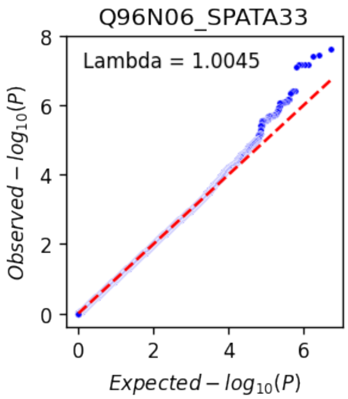

384
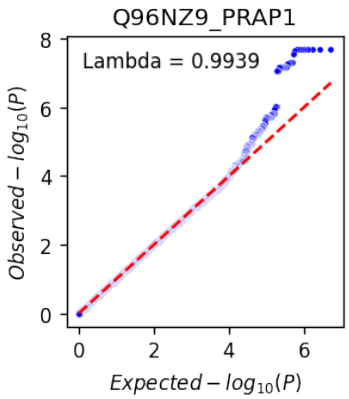

385

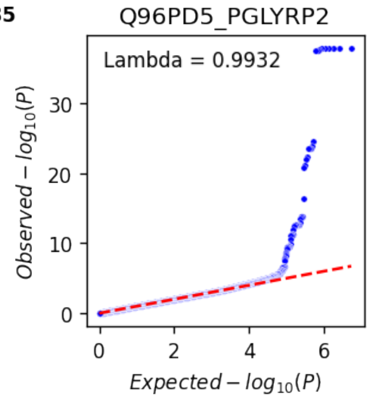

386

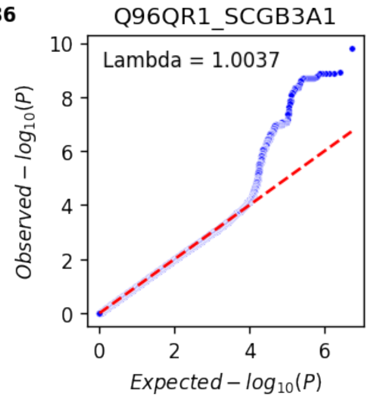

387

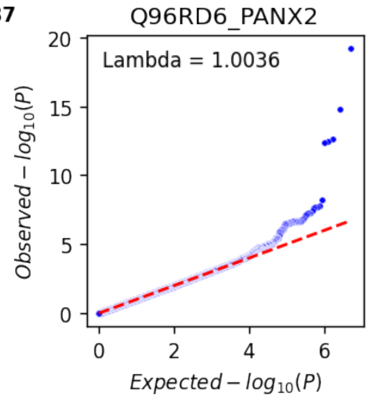

388

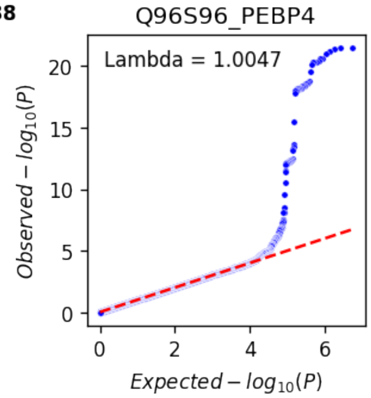

389

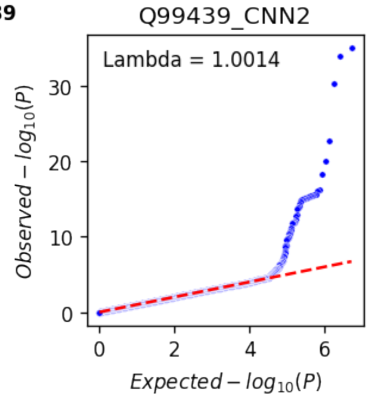

390

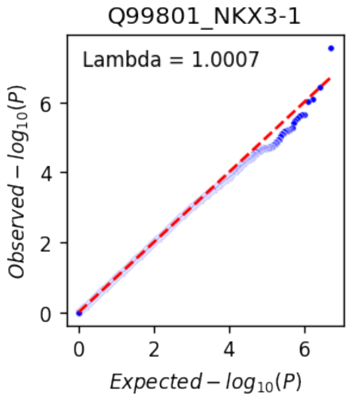

391

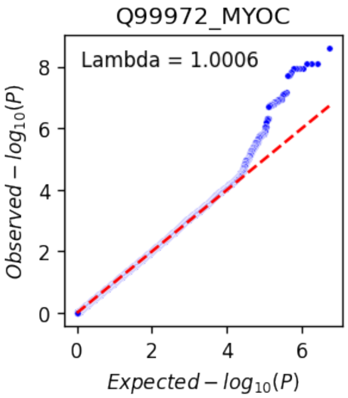

392

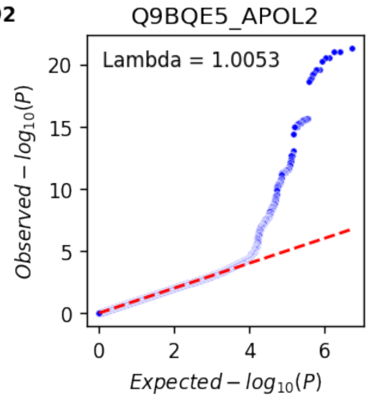

393

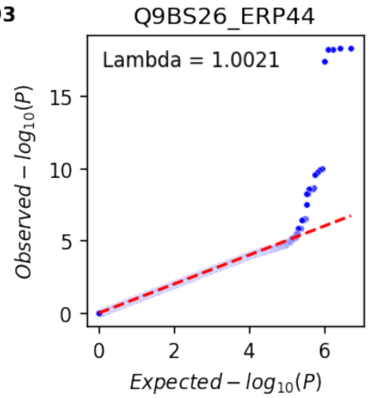

394

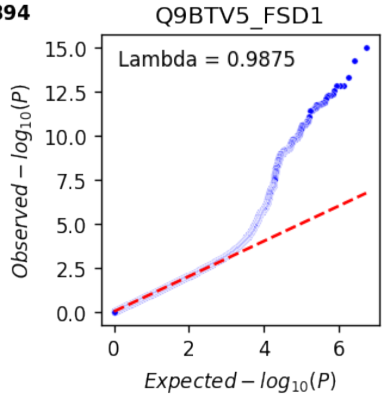

395

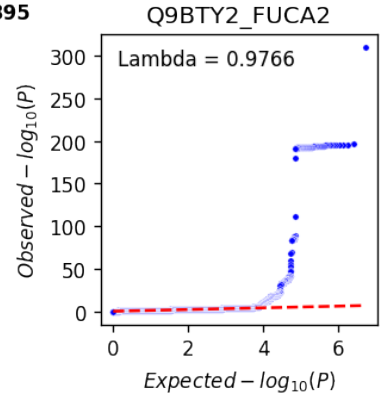

396

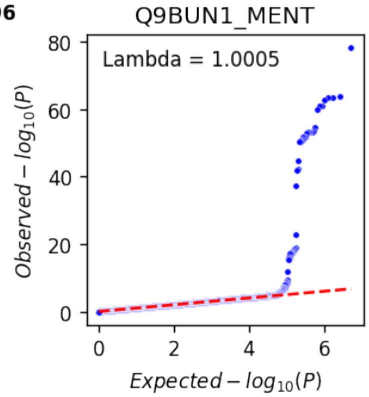

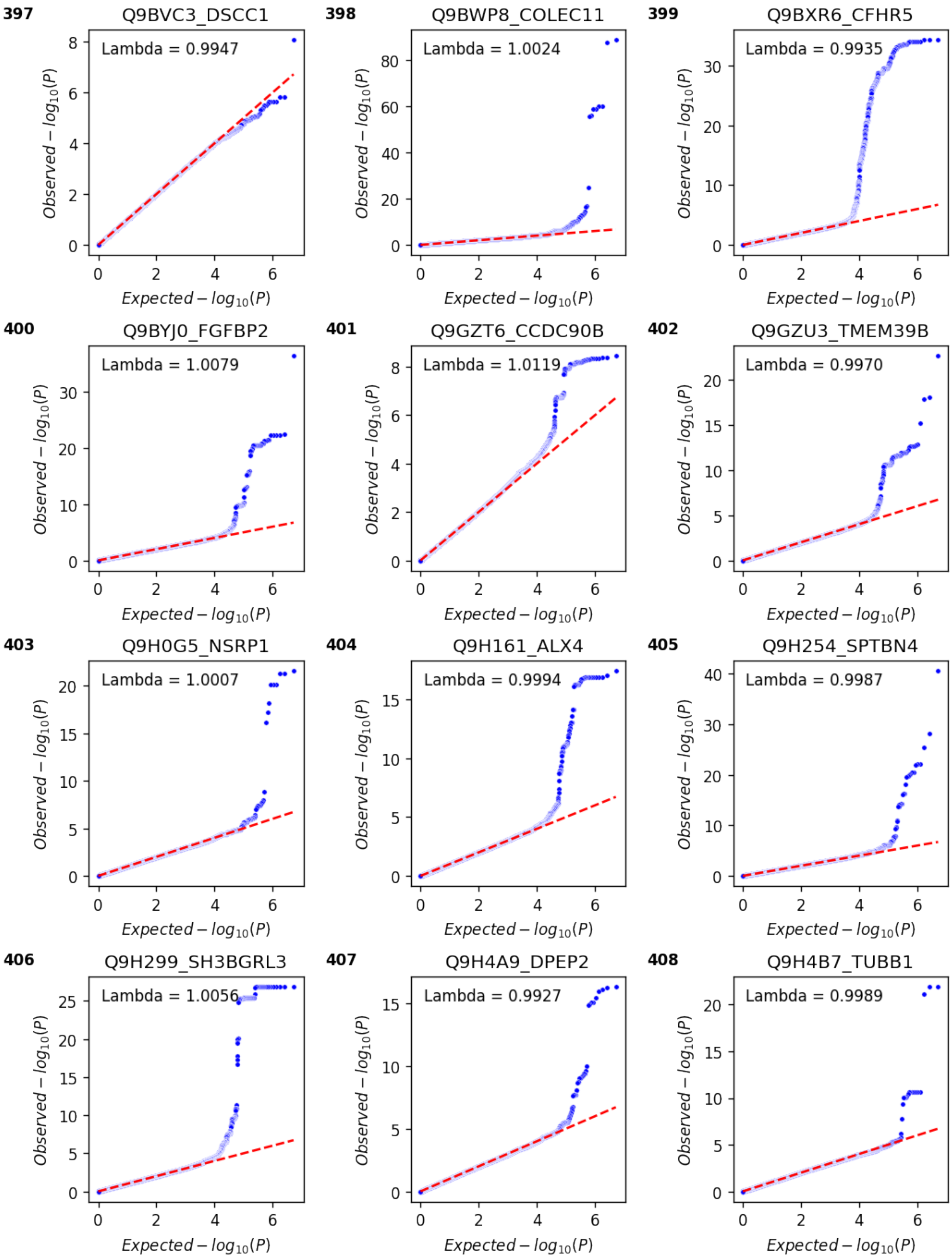
397
Q9BVC3_DSCC1
Lambda = 0.9947
398
Q9BWP8_COLEC11
Lambda = 1.0024
399
Q9BXR6_CFHR5
Lambda = 0.9935
400
Q9BYJ0_FGFBP2
Lambda = 1.0079
401
Q9GZT6_CCDC90B
Lambda = 1.0119
402
Q9GZU3_TMEM39B
Lambda = 0.9970
403
Q9H0G5_NSRP1
Lambda = 1.0007
404
Q9H161_ALX4
Lambda = 0.9994
405
Q9H254_SPTBN4
Lambda = 0.9987
406
Q9H299_SH3BGRL3
Lambda = 1.0056
407
Q9H4A9_DPEP2
Lambda = 0.9927
408
Q9H4B7_TUBB1
Lambda = 0.9989
Observed − log10(P)
Expected − log10(P)

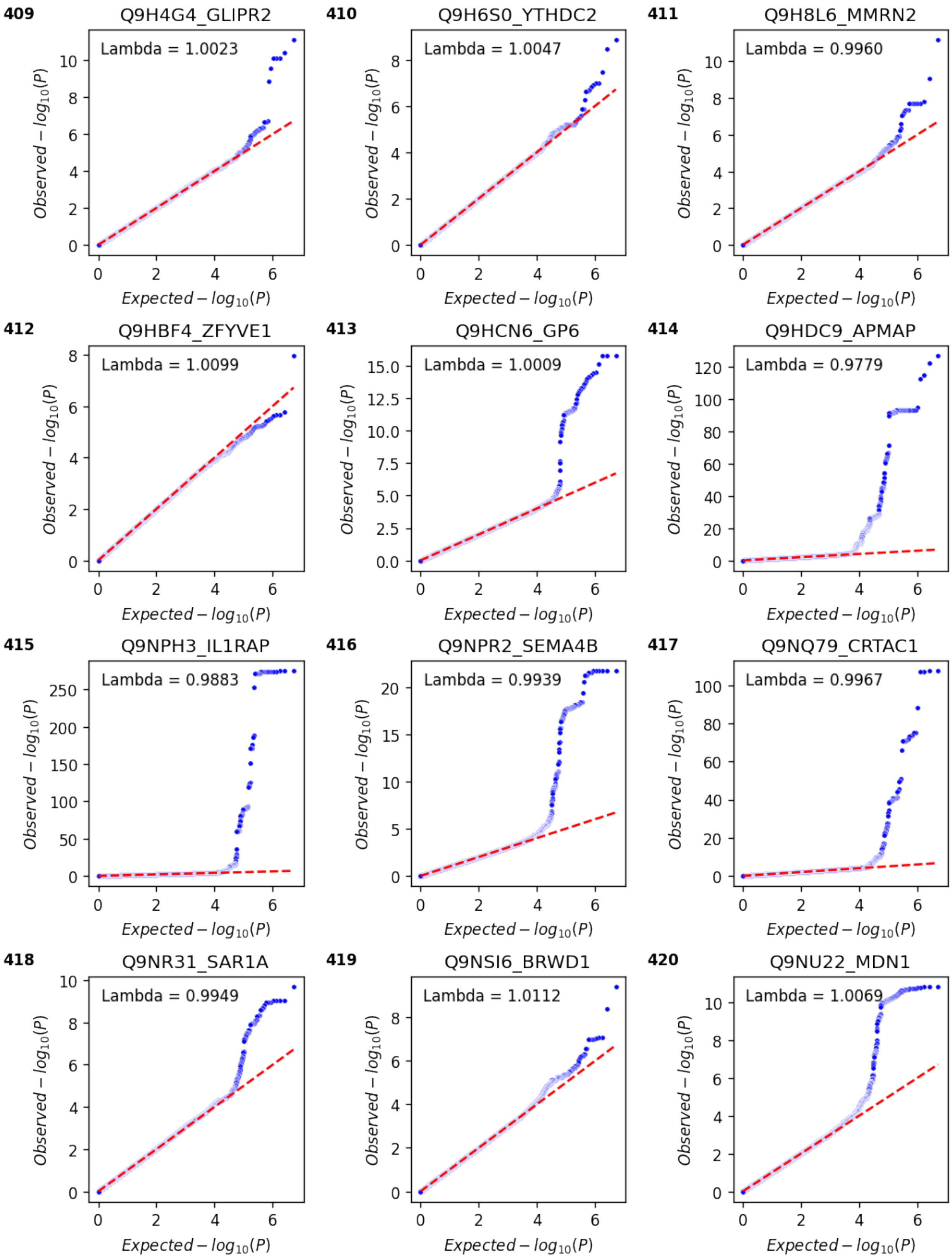
409
Q9H4G4_GLIPR2
Lambda = 1.0023
410
Q9H6S0_YTHDC2
Lambda = 1.0047
411
Q9H8L6_MMRN2
Lambda = 0.9960
412
Q9HBF4_ZFYVE1
Lambda = 1.0099
413
Q9HCN6_GP6
Lambda = 1.0009
414
Q9HDC9_APMAP
Lambda = 0.9779
415
Q9NPH3_IL1RAP
Lambda = 0.9883
416
Q9NPR2_SEMA4B
Lambda = 0.9939
417
Q9NQ79_CRTAC1
Lambda = 0.9967
418
Q9NR31_SAR1A
Lambda = 0.9949
419
Q9NSI6_BRWD1
Lambda = 1.0112
420
Q9NU22_MDN1
Lambda = 1.0069
Observed − $log_{10}(P)$
Expected − $log_{10}(P)$

421
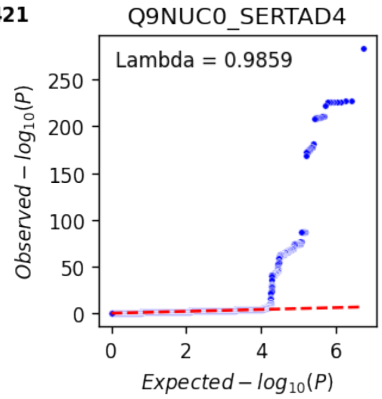

422
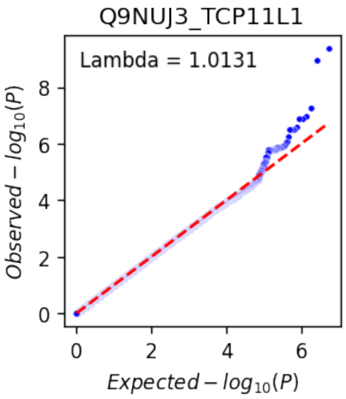

423
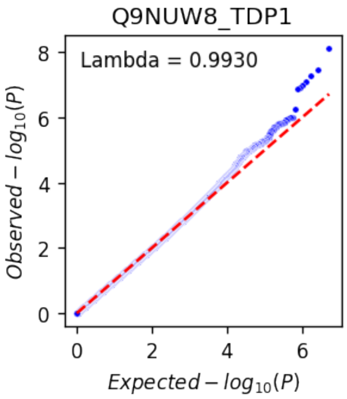

424
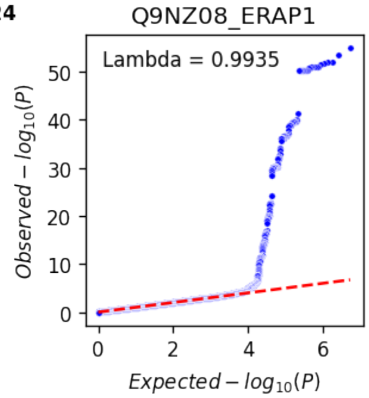

425
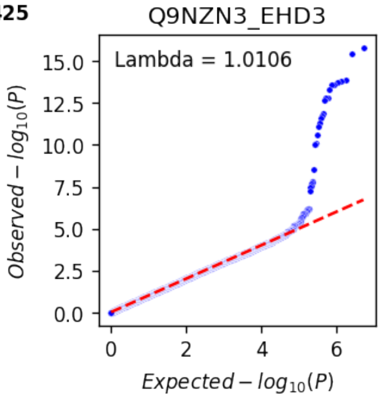

426
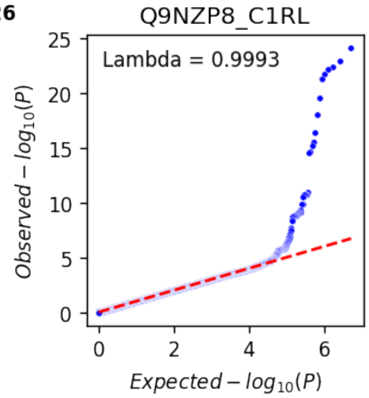

427
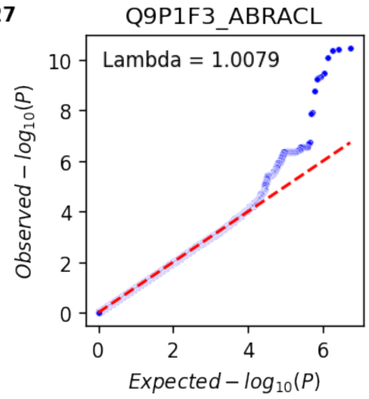

428
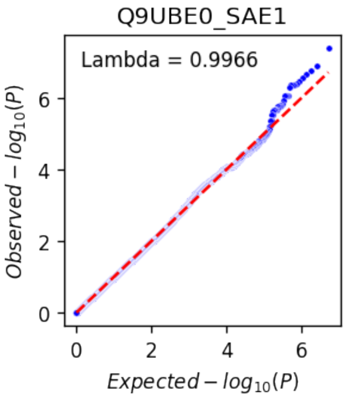

429
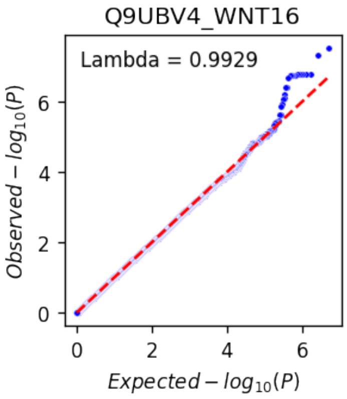

430
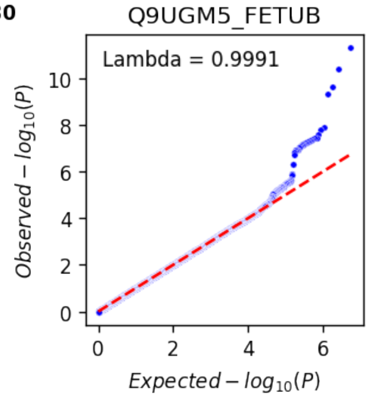

431
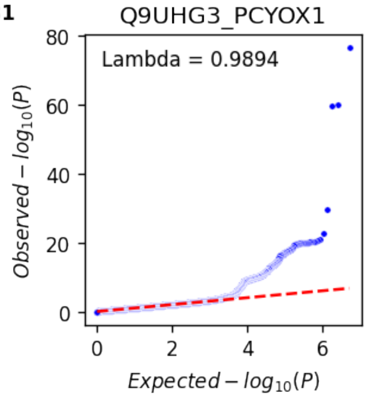

432
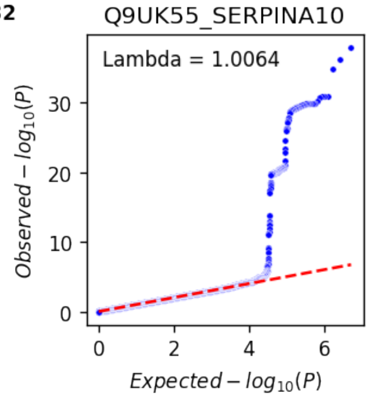

433

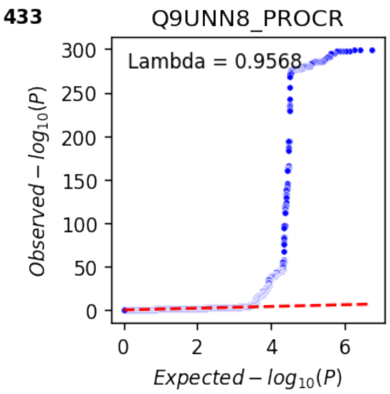

434

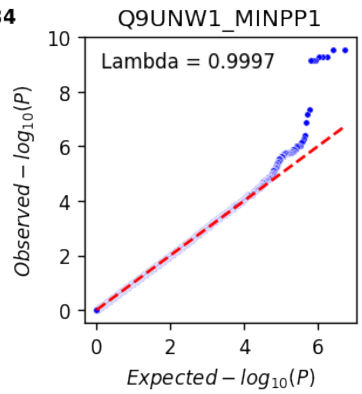

435

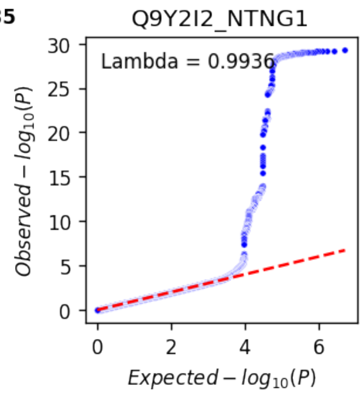

436

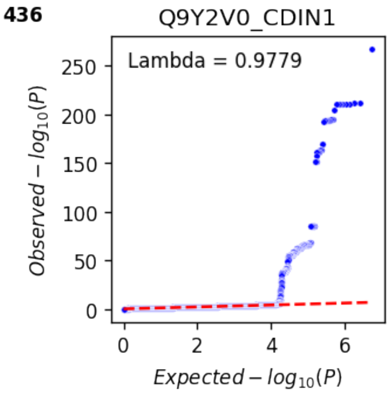

437

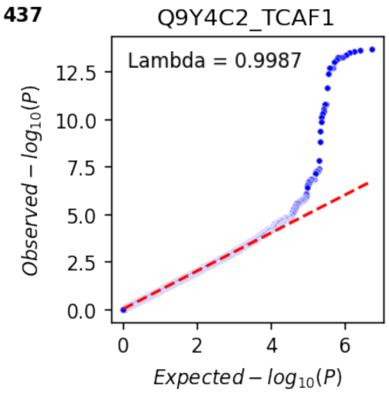

438

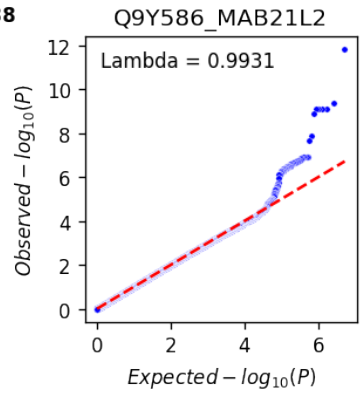

439

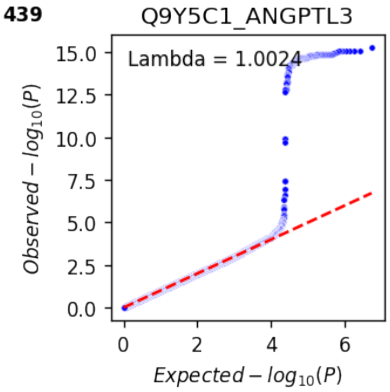

440

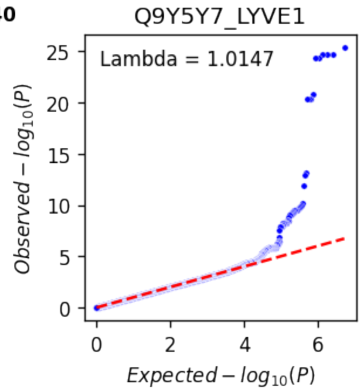

441

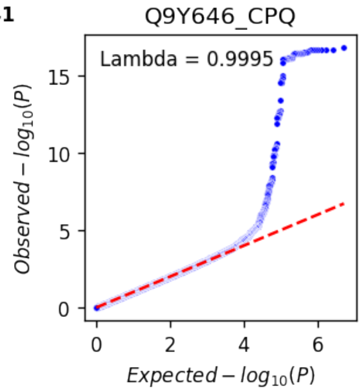

442

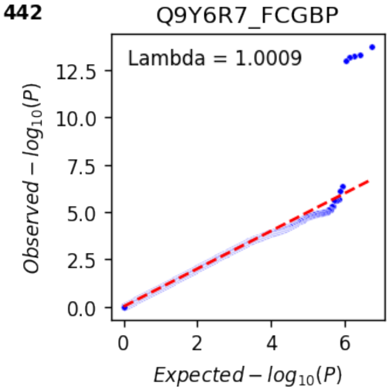

443

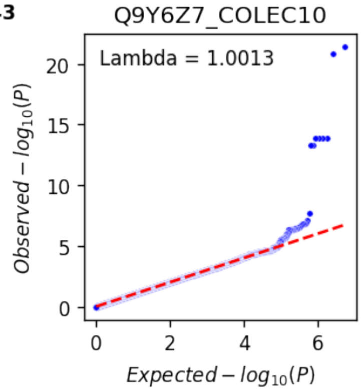

444

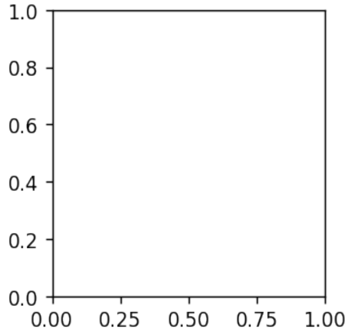

Supplementary Note 6: Comparison of measurements across instruments and time points

Comparison with our previous dataset, generated using an older generation of mass spectrometers[18], reveals that our methodology produces highly consistent protein quantification across different time points and instrumentations. This consistency supports the robustness of our findings and suggests that our approach can reliably detect biological variation even with technological advancements or delays in sample analysis. The comparisons are detailed below.

1. We successfully replicated 82% of the pQTLs for the overlapping proteins (77%) in the new dataset at genome-wide significance (5e-8), despite modifications in our search and quantification strategy.

2. Using a subset of 96 samples measured three months apart on old and new instrumentation, we observed a high protein-wise cross-platform median Pearson correlation coefficient of 0.89 among 408 overlapping proteins (**Extended Data Fig. 3c**), and even higher sample-wise proteome correlations **(Extended Data Fig. 3a**).

3. To assess the impact of longer-term storage, we analyzed another set of 96 samples measured 2 years apart. Despite this extended delay, we still found a robust median Pearson correlation of 0.79 among 465 overlapping proteins (**Extended Data Fig. 3d**), with higher sample-wise proteome correlations (**Extended Data Fig. 3b**)

Supplementary Note 7: GWAS summary statistics datasets used in this study

Summary statistics file	Trait	TA	Study (PMID)	Download link/GWAS Catalogue Accession number
33532862-GCST011365-EFO_0000612-Build37.f.tsv	Myocardial_infarction	ASCVD	33532862	GCST011365
AAA_NatGenet2023.harmonized.txt	Abdominal_Aoritic_Aneurysm	ASCVD	37845353	https://csg.sph.umich.edu/willer/public/AAAgen2023/
biomarkers-30610-both_sexes-irnt.tsv	ALP	MASH	Pan-UK Biobank	https://biobank.ctsu.ox.ac.uk/ukb/
biomarkers-30620-both_sexes-irnt.tsv	ALT	MASH	Pan-UK Biobank	https://biobank.ctsu.ox.ac.uk/ukb/
biomarkers-30650-both_sexes-irnt.tsv	AST	MASH	Pan-UK Biobank	https://biobank.ctsu.ox.ac.uk/ukb/
biomarkers-30670-both_sexes-irnt.tsv	Urea	CKD	Pan-UK Biobank	https://biobank.ctsu.ox.ac.uk/ukb/
biomarkers-30710-both_sexes-irnt.tsv	C_reactive_protein	MASH	Pan-UK Biobank	https://biobank.ctsu.ox.ac.uk/ukb/
biomarkers-30730-both_sexes-irnt.tsv	GGT	MASH	Pan-UK Biobank	https://biobank.ctsu.ox.ac.uk/ukb/
biomarkers-30740-both_sexes-irnt.tsv	Glucose	Diabetes	Pan-UK Biobank	https://biobank.ctsu.ox.ac.uk/ukb/
biomarkers-30750-both_sexes-irnt.tsv	HbA1c	Diabetes	Pan-UK Biobank	https://biobank.ctsu.ox.ac.uk/ukb/
biomarkers-30780-both_sexes-irnt.tsv	LDL	Obesity	Pan-UK Biobank	https://biobank.ctsu.ox.ac.uk/ukb/
biomarkers-30870-both_sexes-irnt.tsv	TRIG	Obesity	Pan-UK Biobank	https://biobank.ctsu.ox.ac.uk/ukb/
biomarkers-30880-both_sexes-irnt.tsv	Urate	CKD	Pan-UK Biobank	https://biobank.ctsu.ox.ac.uk/ukb/

bmi.giant-ukbb.meta-analysis.combined.23May2018.txt	BMI_ukbb_giant	Obesity	30124842	https://portals.broadinstitute.org/collaboration/giant/index.php/GIANT_consortium_data_files
CAD_Aragam_ME_2022_harmonized.txt	CAD	ASCVD	36474045	http://www.cardiogramplusc4d.org/
Cirrhosis_Ghouse_NatGenet2024.ME.harmonized.txt	Cirrhosis2024	MASH	38632349	GCST90319877 and GCST90319878
CKD_overall_EA_JW_20180223_nstud23.dbgap_harmonized.txt	CKD	CKD	31152163	http://ckdgen.imbi.uni-freiburg.de
continuous-23098-both_sexes-irnt.tsv	Weight	Obesity	Pan-UK Biobank	https://biobank.ctsu.ox.ac.uk/ukb/
continuous-4079-both_sexes-irnt.tsv	DBP	CKD	Pan-UK Biobank	https://biobank.ctsu.ox.ac.uk/ukb/
continuous-4080-both_sexes-irnt.tsv	SBP	CKD	Pan-UK Biobank	https://biobank.ctsu.ox.ac.uk/ukb/
continuous-48-both_sexes-irnt.tsv	Waist_circumference	Obesity	Pan-UK Biobank	https://biobank.ctsu.ox.ac.uk/ukb/
continuous-49-both_sexes-irnt.tsv	Hip_circumference	Obesity	Pan-UK Biobank	https://biobank.ctsu.ox.ac.uk/ukb/
ffmi_sexcomb.ukbb.EUR.LGLM.2023.txt	Fat-free mass index	Obesity	Pan-UK Biobank	https://biobank.ctsu.ox.ac.uk/ukb/
bfpct_sexcomb.ukbb.EUR.LGLM.2023.txt	Body fat percent	Obesity	Pan-UK Biobank	https://biobank.ctsu.ox.ac.uk/ukb/
all_cause_hf_all_NatGenet2024.harmonized.txt	All cause heart failure	ASCVD	medRxiv preprint	https://doi.org/10.1101/2023.10.01.23296379
eGFRcrea_GWAS_S1.5million_Summary_Statistics_harmonized.txt	eGFR	CKD	35710981	https://susztaklab.com/GWAS
GCST90018959_buildGRCh37_harmonized.tsv	Height	Obesity	34594039	GCST90018959
GCST90027158_buildGRCh37_harmonized.tsv	Alzheimer	AD	35379992	GCST90027158

GCST90029073_buildGRCh37.tsv	Liver_fat_accumulation	MASH	34957434	GCST90029073
HF-multiancestry-maf0.01_harmonized.txt	Heart_Failure	ASCVD	36376295	GCST90162626
IschaemicStroke_Mishra_2022_ALL_3618079595	Ischaemic_Stroke	ASCVD	36180795	GCST90104534–GCST90104563
Lagou_RandomGlucose_Met_EUR.hg19_harmonized.tsv	RandomGlucose	Diabetes	37679419	https://www.magicinvestigators.org/
MAGIC1000G_2hGlu_EUR	2hGlu	Diabetes	34059833	https://www.magicinvestigators.org/
MAGIC1000G_FG_EUR	FastingGlucose	Diabetes	34059833	https://www.magicinvestigators.org/
MAGIC1000G_FI_EUR	Fasting_Insulin	Diabetes	34059833	https://www.magicinvestigators.org/
MRI_lvef_filtered_harmonized	MRI_lvef	ASCVD	32382064	http://www.broadcvdi.org/
MRI_lvesvi_filtered_harmonized	MRI_lvesvi	ASCVD	32382064	http://www.broadcvdi.org/
MVP.T2D.EUR.MAF001.dbGaP.txt	T2D_MVP_EUR	Diabetes	32541925	dbGAP under accession number phs001672.v3.p1
Nauffal_2023_myocardial_fibrosis_t1_time.tsv	myocardial_fibrosis	ASCVD	37081215	http://www.broadcvdi.org/
pad_vanzuydam_2020_34601942_harmonized	Peripheral_Artery_Disease	ASCVD	34601942	10.6084/m9.figshare.7811639
Stroke_Mishra_2022_ALL_3618079595	Stroke	ASCVD	36180795	GCST90104534–GCST90104563
T1D_FinnGen_UKBB.harmonized.txt	T1D_GCST90014023	Diabetes	34012112	GCST90014023
T1D_GCST90014023_B38_harmonized.tsv	T1D_FinnGen_UKBB	Diabetes	34012112	GCST90014023

T2D.Suzuki.Nature.EUR_hg19_harmonized.tsv	T2D_Suzuki_EUR	Diabetes	38374256	http://www.diagram-consortium.org/downloads.html
T2D.Suzuki.Nature.ME_hg19_harmonized.tsv	T2D_Suzuki_ME	Diabetes	38374256	http://www.diagram-consortium.org/downloads.html
whradjbmi.giant-ukbb.meta-analysis.combined.23May2018.txt	whradjbmi	Obesity	30239722	https://github.com/lindgrengroup/fatdistnGWAS
t2d_30054458-GCST006867-EFO_0001360-build37.f.tsv	T2D4	T2D	30054458	GCST006867
NAFLD_34841290-GCST90091033-EFO_0003095-Build37.f.tsv	NAFLD	NAFLD	34841290	GCST90091033
heelbonemineral_30598549-GCST006979-EFO_0009270-build37.f.tsv	Heel_Bone_Mineral	NA	30598549	GCST006979

Supplementary Note 8: Replication of pQTLs in children and adults

Plasma proteomics sample preparation, data acquisition, and preprocessing of genotype and proteomics data, along with genome-wide association testing in the 1,000 children replication set and the independent adult cohort, were conducted similarly to the discovery cohort where applicable, unless otherwise specified. After quality control of the genomic data, the final number of individuals included in the genome-wide association tests was *n*=990 for children and adolescents and *n*=554 for adults. The study protocol for the GALAXY replication cohorts was approved by the ethics committee for the Region of Southern Denmark (nos. S-20160006G, S-20120071, S-20160021 and S-20170087) and is registered with both the Danish Data Protection Agency (nos. 13/8204, 16/3492 and 18/22692) and Odense Patient Data Exploratory Network (under study identification nos. OP_040 and OP_239 (open.rsyd.dk/OpenProjects/da/openProjectList.jsp)). The study was conducted according to the principles of the Declaration of Helsinki, and oral and written informed consent was obtained from all participants. Differences in proteomics data processing in the adult cohort include 1) liver disease status (histological scoring of fibrosis F0-F4, inflammatory activity I0-5 and steatosis score S0-3), alcohol abstinent status, and statin use status were further controlled for at the protein level in addition to age, BMI and, sex and 2) genotype imputation was performed using minimac4 (version 1.0.2) and quality was filtered at R2>0.5, which has been shown to be a good threshold for separating between poorly and well-imputed variants and equivalent to an INFO score>0.7[19,20].

Of the 1,947 primary pQTLs found in the discovery cohort, ~90% were testable in the replication studies. A pQTL was considered replicated if the same SNP or its proxy (r^2>0.2 within a region +/- 1Mb) is also significantly associated (p<0.05) with the protein with a concordant direction of effect. Applying a Bonferroni corrected *p-value* of 2.9e-5 led to a replication rate of 76% and 54% in children and adults, respectively.

References for supplementary notes

1. Schinzari, F., Vizioli, G., Campia, U., Tesauro, M. & Cardillo, C. Variable Changes of Circulating ANGPTL3 and ANGPTL4 in Different Obese Phenotypes: Relationship with Vasodilator Dysfunction. *Biomedicines* 9, (2021).

2. Arab Sadeghabadi, Z. *et al.* Angiopoietin-Like Proteins 2 and 3 in Children and Adolescents with Obesity and Their Relationship with Hypertension and Metabolic Syndrome. *Int J Hypertens* 2021, 6748515 (2021).

3. Garcés, M. F. *et al.* Serum angiopoietin-like 3 levels are elevated in obese non diabetic men but are unaffected during an oral glucose tolerance test. *Sci Rep* 10, 21118 (2020).

4. Cinkajzlová, A. *et al.* Angiopoietin-like protein 3 and 4 in obesity, type 2 diabetes mellitus, and malnutrition: the effect of weight reduction and realimentation. *Nutr Diabetes* 8, 21 (2018).

5. Purcell, S. *et al.* PLINK: a tool set for whole-genome association and population-based linkage analyses. *Am J Hum Genet* 81, 559–75 (2007).

6. Yang, J. *et al.* Conditional and joint multiple-SNP analysis of GWAS summary statistics identifies additional variants influencing complex traits. *Nat Genet* 44, 369–75, S1-3 (2012).

7. Bretherick, A. D. *et al.* Linking protein to phenotype with Mendelian Randomization detects 38 proteins with causal roles in human diseases and traits. *PLoS Genet* 16, e1008785 (2020).

8. Katz, D. H. *et al.* Whole Genome Sequence Analysis of the Plasma Proteome in Black Adults Provides Novel Insights Into Cardiovascular Disease. *Circulation* 145, 357–370 (2022).

9. Suhre, K. *et al.* Connecting genetic risk to disease end points through the human blood plasma proteome. *Nat Commun* 8, 14357 (2017).

10. Yao, C. *et al.* Genome-wide mapping of plasma protein QTLs identifies putatively causal genes and pathways for cardiovascular disease. *Nat Commun* 9, 3268 (2018).

11. Zhong, W. *et al.* Whole-genome sequence association analysis of blood proteins in a longitudinal wellness cohort. *Genome Med* 12, 53 (2020).

12. Sun, B. B. *et al.* Genomic atlas of the human plasma proteome. *Nature* 558, 73–79 (2018).

13. Sun, B. B. *et al.* Plasma proteomic associations with genetics and health in the UK Biobank. *Nature* 622, 329–338 (2023).

14. Ferkingstad, E. *et al.* Large-scale integration of the plasma proteome with genetics and disease. *Nat Genet* 53, 1712–1721 (2021).

15. Gudjonsson, A. *et al.* A genome-wide association study of serum proteins reveals shared loci with common diseases. *Nat Commun* 13, 480 (2022).

16. Pietzner, M. *et al.* Genetic architecture of host proteins involved in SARS-CoV-2 infection. *Nat Commun* 11, 6397 (2020).

17. Emilsson, V. *et al.* Coding and regulatory variants are associated with serum protein levels and disease. *Nat Commun* 13, 481 (2022).

18. Niu, L. *et al.* Plasma Proteome Variation and its Genetic Determinants in Children and Adolescents. *medRxiv* 2023.03.31.23287853 (2023) doi:10.1101/2023.03.31.23287853.

19. Li, Y., Willer, C. J., Ding, J., Scheet, P. & Abecasis, G. R. MaCH: using sequence and genotype data to estimate haplotypes and unobserved genotypes. *Genet Epidemiol* 34, 816–834 (2010).

20. Guindo-Martínez, M. *et al.* The impact of non-additive genetic associations on age-related complex diseases. *Nat Commun* 12, 2436 (2021).

Supplementary Figure 1

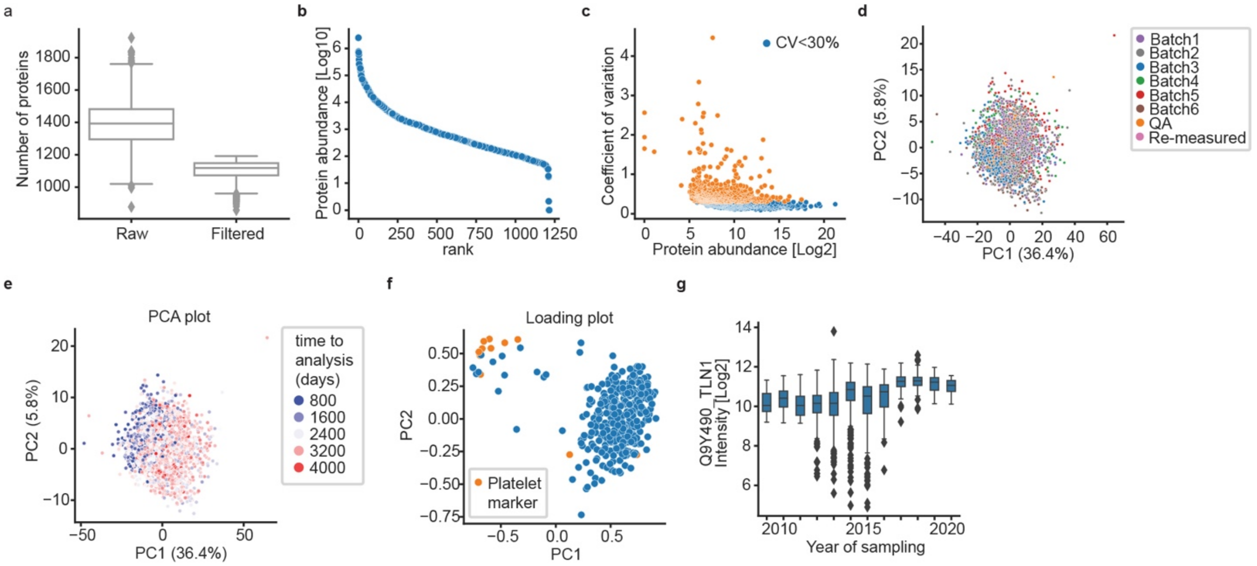

Supplementary Fig. 1. Proteomics data quality in the discovery cohort. a, The number of proteins quantified in each sample before and after filtering for data completeness at protein level. *n*=2,145 biologically independent samples. **b,** Protein intensity as a function of abundance rank. **c,** The coefficients of variation (CV) of each protein assessed by quality assessment (QA) samples are plotted against their median intensity. *n*=94 workflow replicates. **d-e,** Principal component analysis (PCA) for the plasma proteome profile of all samples, showing the first and second principal component. **f,** Loading plot for the principal component analysis (PCA) in Panel (d-e) with platelet markers highlighted. **g,** Abundance of TLN1 by year of sampling, with samples being collected in more recent years having higher levels of TLN1. The number of biological replicates for each year from 2009 to 2020 is as follows: 28, 40, 36, 399, 432, 474, 222, 165, 146, 80, 80, 26. For Panel (a) and (g), The gray line in the middle of the box is the median, the top and bottom of the box represent the upper and lower quartile values of the data and the whiskers represent the upper and lower limits for consideration of outliers ($Q3 + 1.5 \times IQR$, $Q1 - 1.5 \times IQR$). IQR represents the interquartile range ($Q3 - Q1$).

Supplementary Figure 2

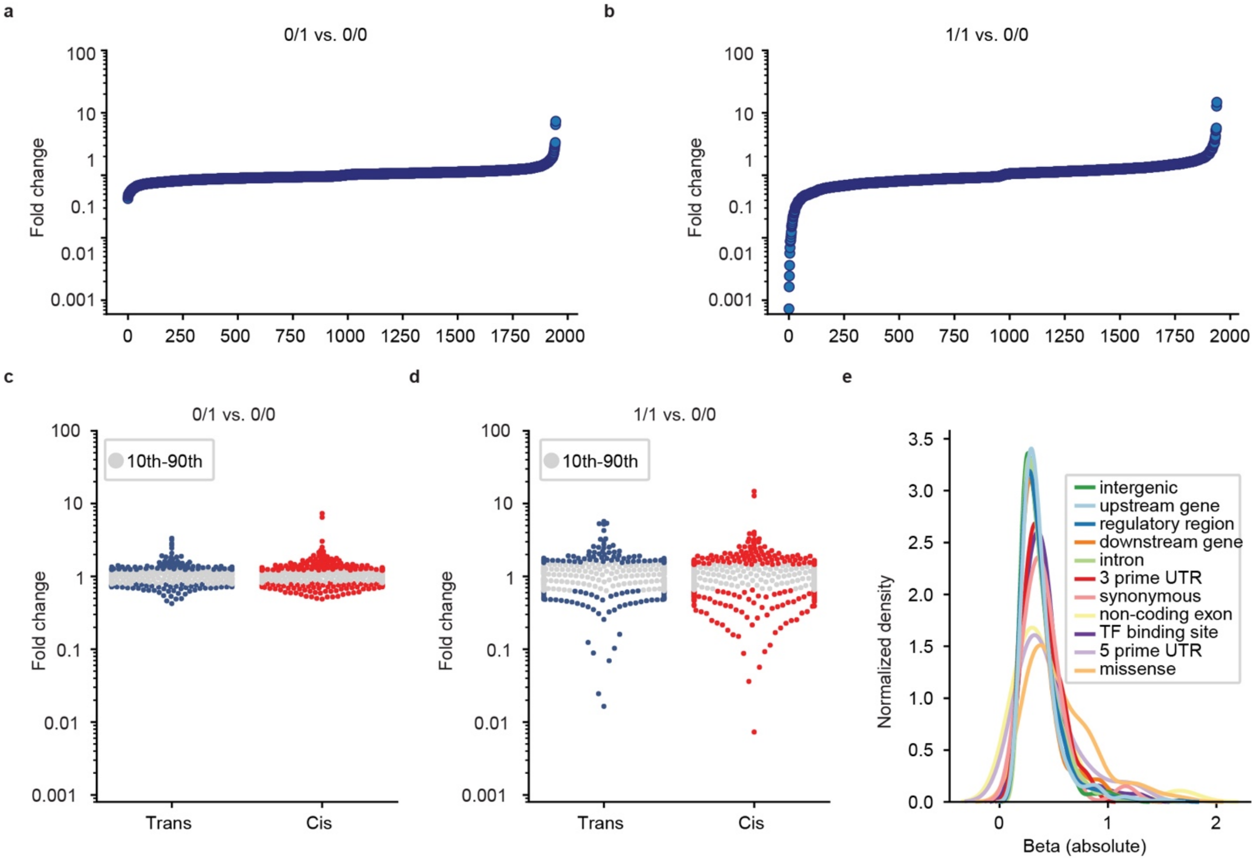

Supplementary Fig. 2. Characteriztion of pQTL effect sizes. a, Fold change of protein levels between heterozygous variant alleles and homozygous reference alleles for all primary protein quantitative trait loci (pQTLs). **b,** Fold change of protein levels between homozygous variant alleles and homozygous reference alleles for all primary pQTLs. **c-d,** Swarmplot showing fold change of protein levels stratified by cis- and trans-pQTLs. Values within 10th-90th percentiles are indicated. **e,** Kernel density estimation plot of beta statistics in absolute value stratified by variant categories. UTR: untranslated region; TF: transcription factor.

Supplementary Figure 3

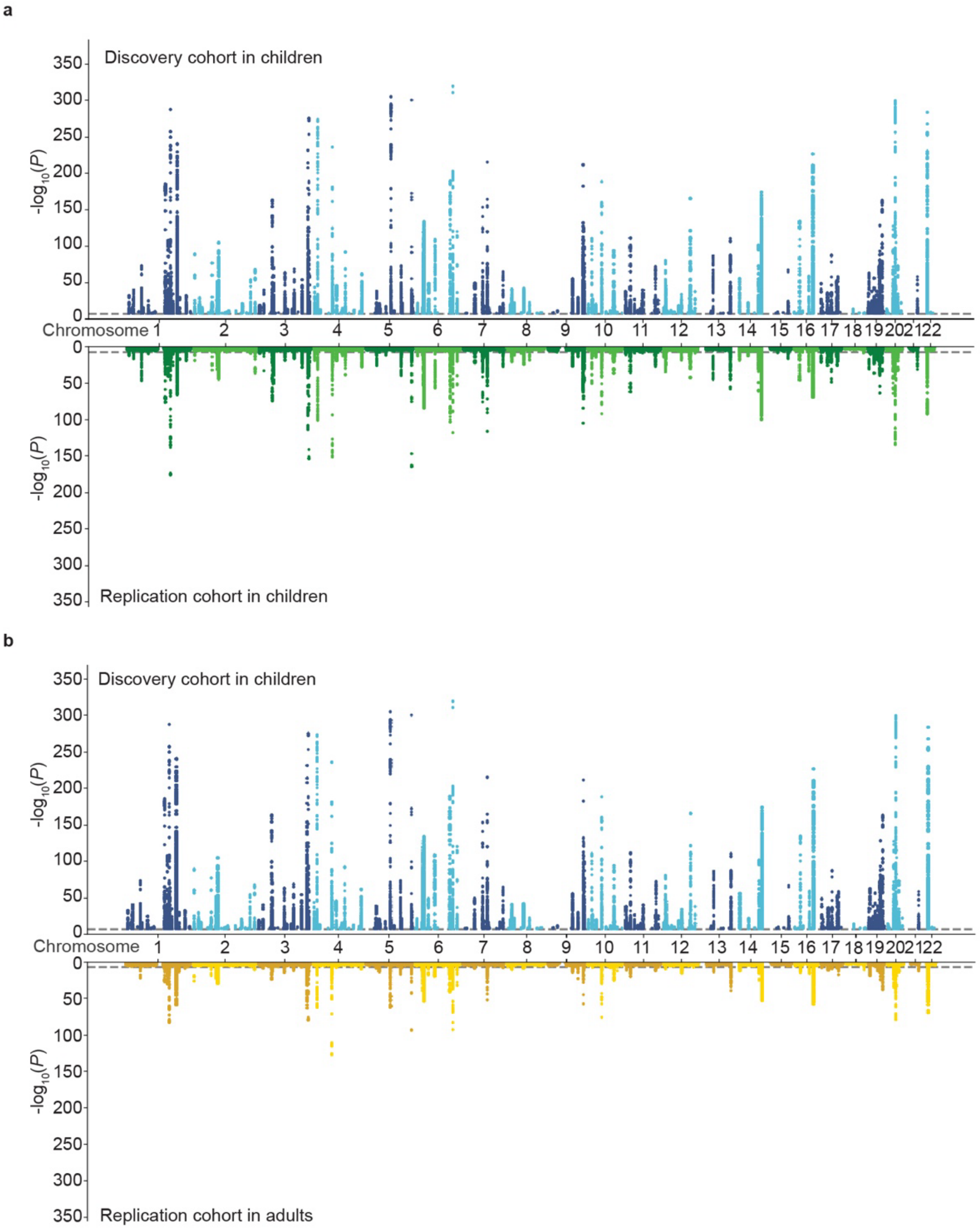

Supplementary Fig. 3. **Replication of pQTLs in children and adults. a-b,** Miami plot showing genetic signals for all protein quantitative trait loci (pQTLs) in the discovery cohort and those replicated children and adolescents (*n*=990 biological replicates) in Panel (a) and adults (*n*=554 biological replicates) in Panel (b).
