# Supplementary material for: Plasma proteome variation and its genetic determinants in children and adolescents
Source: Nat Genet. 2025 Feb 19;57(3):635–46. doi: 10.1038/s41588-025-02089-2 (PMC11906355; doi:10.1038/s41588-025-02089-2)
Supplement: Supplementary file 2 — Reporting Summary [file 41588_2025_2089_MOESM2_ESM.pdf]

nature portfolio

Corresponding author(s): Simon Rasmussen, Torben Hansen, Matthias Mann

Last updated by author(s): Oct 16, 2024

Reporting Summary

Nature Portfolio wishes to improve the reproducibility of the work that we publish. This form provides structure for consistency and transparency in reporting. For further information on Nature Portfolio policies, see our Editorial Policies and the Editorial Policy Checklist.

Statistics

For all statistical analyses, confirm that the following items are present in the figure legend, table legend, main text, or Methods section.

n/a	Confirmed	
☐	☒	The exact sample size (*n*) for each experimental group/condition, given as a discrete number and unit of measurement
☐	☒	A statement on whether measurements were taken from distinct samples or whether the same sample was measured repeatedly
☐	☒	The statistical test(s) used AND whether they are one- or two-sided<br>*Only common tests should be described solely by name; describe more complex techniques in the Methods section.*
☐	☒	A description of all covariates tested
☐	☒	A description of any assumptions or corrections, such as tests of normality and adjustment for multiple comparisons
☐	☒	A full description of the statistical parameters including central tendency (e.g. means) or other basic estimates (e.g. regression coefficient) AND variation (e.g. standard deviation) or associated estimates of uncertainty (e.g. confidence intervals)
☐	☒	For null hypothesis testing, the test statistic (e.g. *F*, *t*, *r*) with confidence intervals, effect sizes, degrees of freedom and *P* value noted<br>*Give P values as exact values whenever suitable.*
☐	☒	For Bayesian analysis, information on the choice of priors and Markov chain Monte Carlo settings
☒	☐	For hierarchical and complex designs, identification of the appropriate level for tests and full reporting of outcomes
☐	☒	Estimates of effect sizes (e.g. Cohen's *d*, Pearson's *r*), indicating how they were calculated

Our web collection on statistics for biologists contains articles on many of the points above.

Software and code

Policy information about availability of computer code

Data collection	The commercial software Spectronaut (v18.4) was used to perform protein identification and quantification.
Data analysis	The software used in this study can be accessed here: EAGLE2 (v2.0.5): https://alkesgroup.broadinstitute.org/Eagle/; Spectronaut (v15.4): https://biognosys.com/software/spectronaut/; Python (v3.9.0 and 3.8.11): https://www.python.org/; Combat (v0.3.2): https://pypi.org/project/combat/; Gseapy (v1.1.1): https://gseapy.readthedocs.io; Seaborn (v0.12.2): https://seaborn.pydata.org/; QLattice implemented in the Feyn Python module (v3.0.1): https://www.abzu.ai/qlattice/; Compute-lambda.py (v2.0): https://github.com/pgxcentre/lambda; Bcftools (v1.14): https://samtools.github.io/bcftools/; Gemma (v0.98.5): https://github.com/genetics-statistics/GEMMA; Plink (v1.90b6.24): https://www.cog-genomics.org/plink/; GCTA-COJO (v1.93.3): https://yanglab.westlake.edu.cn/software/gcta; Variant effect predictor was performed online on March 02, 2024 with the RefSeq transcript database (http://grch37.ensembl.org/Homo_sapiens/Tools/VEP); Scikit-learn (v1.0): https://scikit-learn.org/stable/whats_new/v1.0.html; INT-transformation (v2019.08.21): https://github.com/edm1/rank-based-INT; Scipy (v1.7.1): https://scipy.org/; Pingouin (v0.4.0 and v0.5.4): https://pingouin-stats.org/build/html/index.html; Statsmodels (v0.13.0): https://www.statsmodels.org/stable/index.html; Pyliftover (v0.4): https://pypi.org/project/pyliftover/; GeneLocator (v1.1.2): https://pypi.org/project/GeneLocator/; Hyprcoloc (v1.0.0): https://github.com/cnfoley/hyprcoloc; TwoSampleMR (v0.5.6): https://mrcieu.github.io/TwoSampleMR; The customed scripts can be accessed at github.com/llniu/pQTL_HolbaekStudy.

For manuscripts utilizing custom algorithms or software that are central to the research but not yet described in published literature, software must be made available to editors and reviewers. We strongly encourage code deposition in a community repository (e.g. GitHub). See the Nature Portfolio guidelines for submitting code & software for further information.

Data

Policy information about availability of data

All manuscripts must include a data availability statement. This statement should provide the following information, where applicable:

- Accession codes, unique identifiers, or web links for publicly available datasets
- A description of any restrictions on data availability
- For clinical datasets or third party data, please ensure that the statement adheres to our policy

The GWAS summary statistics generated in this study have been uploaded to the GWAS Catalog (https://www.ebi.ac.uk/gwas/), under accession IDs GCST90452968 to GCST90454170, and will be available upon publication. Accession IDs or download links for publicly available GWAS summary statistics datasets used in this study are listed in Supplementary Note 7. The canonical human reference proteome database (2023.05 release) was downloaded from the European Bioinformatics Institute database (https://ftp.ebi.ac.uk/pub/databases/reference_proteomes/); Tissue specificity annotation of proteins was downloaded from the Human Protein Atlas database (https://www.proteinatlas.org/about/download). The GWAS Catalog (v1.0.2) was downloaded at https://www.ebi.ac.uk/gwas/docs/file-downloads; Transcription start site of proteins was extracted from BioMart (accessed on November 23, 2023) (https://grch37.ensembl.org/info/data/biomart/index.html); The gene sets used for mapping biological processes and functions can be accessed via Gseapy (v1.1.1) using the identifiers 'MSigDB_Hallmark_2020' and 'GO_Biological_Process_2023'. All analysis results are available as supplementary tables. Searchable results are publicly accessible at proteomevariation.org. The study protocol is also available upon request to Jens-Christian Holm, jhom@regionsjaelland.dk. Due to GDPR regulations, individual-level clinical metadata, genomics and proteomics data generated in this study cannot be made publicly available but are available upon request to the corresponding authors. The time frame for response to requests from the authors is within a 1-month period. When processing data, certain restrictions apply: (1) a data processing agreement must be signed between the data controller and processor; (2) data must not be processed for purposes other than statistical and scientific studies; and (3) personal data must be deleted, anonymized and destroyed at the end of investigation and must not be passed on to a third party or individuals who are not authorized to access the data.

Human research participants

Policy information about studies involving human research participants and Sex and Gender in Research.

Reporting on sex and gender

Information on sex was self-reported and checked using X-chromosome data using the Plink software. Boys and girls were well-balanced in the discovery cohort (55% girls and 45% boys). Sex-based analysis was performed in testing the effects of sex on the plasma proteome.

Population characteristics

Discovery cohort: n=2,147; 45% males; ages 5-20, median age of 12; median BMI of 21.7. Distribution of covariates: 65% in pubertal/post-pubertal stage (tanner stage 2-5); sample storage time 1.4-12.4 years (median 7.4).
Replication cohort in children: n=1,000 matched by age, sex and overweight status for replication (58% girls and 42% boys).
Replication cohort: n=558; 73% males; ages 19-82, median age of 56; median BMI of 27. Distribution of covariates: fibrosis stage F0/1/2/3/4: 249/118/102/26/63, inflammatory activity I0/1/2/3/4/5: 290/90/78/50/28/22, steatosis S0/1/2/3: 373/79/70/36, 35% abstinent upon inclusion, 17% had treatment of statin prior to inclusion. Note that individuals who were not biopsied due to low liver stiffness as measured by FibroScan (<6.0 kPa) were considered as healthy.

Recruitment

The participants from the discovery cohort were recruited from 1) the Children's Obesity Clinic, Centre of Obesity Management offering the multidisciplinary childhood obesity management program at Copenhagen University Hospital Holbæk and 2) a population-based cohort recruited from schools in 11 municipalities across Zealand, Denmark in a cross-sectional study design. Both groups were enrolled between January 2009 and April 2019. Eligibility criteria for the children in the obesity clinic group were an age of 5–20 years and a BMI above the 90th percentile (BMI SDS >=1.28) according to Danish reference values. Exclusion criteria for this study for both groups are 1) age at recruitment younger than 5 years or older than 20 years; 2) diagnosed type 1 diabetes; 3) diagnosed type 2 diabetes; 4) treatment with medications including insulin, liraglutide, and/or metformin; 5) meeting type 2 diabetes criteria based on the blood sample taken for this study (fasting plasma glucose > 7.0 mmol/L and/or hemoglobin A1c (HbA1c) > 48 mmol/mol.

The study cohort consists solely of individuals of European ancestry. This limitation may affect the generalizability of findings to individuals from other ancestry groups, and further studies in more diverse cohorts would be beneficial for broader applicability. The participants' ages follow a normal distribution with a mean of 12, but there is limited representation (n<30) at the younger (age 5) and older (age 20) extremes. This may affect the accuracy of the age-dependent protein abundance trajectories at these age extremes.

Ethics oversight

The study protocol for the discovery cohort was approved by the ethics committee for the Region Zealand (protocol no. SJ-104) and is registered at the Danish Data Protection Agency (REG-043-2013). The HOLBAEK Study including the obesity clinic cohort and the population-based cohort are also registered at ClinicalTrials.gov (NCT00928473).

The study protocol for the GALAXY replication cohorts was approved by the ethics committee for the Region of Southern Denmark (nos. S-20160006G, S-20120071, S-20160021 and S-20170087) and is registered with both the Danish Data Protection Agency (nos. 13/8204, 16/3492 and 18/22692) and Odense Patient Data Exploratory Network (under study identification nos. OP_040 and OP_239 (open.rsyd.dk/OpenProjects/da/openProjectList.jsp)).

Note that full information on the approval of the study protocol must also be provided in the manuscript.

Field-specific reporting

Please select the one below that is the best fit for your research. If you are not sure, read the appropriate sections before making your selection.

☒ Life sciences ☐ Behavioural & social sciences ☐ Ecological, evolutionary & environmental sciences

For a reference copy of the document with all sections, see nature.com/documents/nr-reporting-summary-flat.pdf

Life sciences study design

All studies must disclose on these points even when the disclosure is negative.

Sample size	n=2,147 for the discovery cohort and n=1,000 for replication in children, n=558 for replication in adults. No sample size calculations were done prior to inclusion of the study participants. The sample size was determined partly based on the throughput of the proteomics workflow and the instrument capacity available in the laboratory at the time. The chosen sample size for discovery was within the range of existing pQTL studies, i.e. between 100s and 10000s. Sample size in the replication cohort was limited by data availability (genotype data, proteomics data and covariates).
Data exclusions	All samples were kept in the analysis unless relevant variables in the corresponding analysis had missing values.
Replication	For plasma proteome profiling in the discovery cohort, 94 pooled plasma samples were analyzed to assess the total technical variability of the proteomics workflow. The study included an additional cohort of 1,000 children and adolescents from the same HOLBAEK Study, as well as an independent adult cohort to replicate the identified pQTLs. Notably, for all biological samples, plasma proteome measurements were performed only once, without additional technical replicates, to capture a single-point measurement per sample.
Randomization	Plasma samples were randomized prior to proteomics sample preparation and data acquisition. Sample storage time was used as a covariate to control for potential systematic bias related to sample storage. Batch correction was further applied to correct for batch effects related to proteomics sample preparation.
Blinding	The investigators who performed proteomics sample preparation and data acquisition were blinded to the participants' genotype information but not age, BMI and sex as randomization in proteomics data generation was anyway performed to avoid systematic bias during the measurement and thus blinding is not relevant.

Reporting for specific materials, systems and methods

We require information from authors about some types of materials, experimental systems and methods used in many studies. Here, indicate whether each material, system or method listed is relevant to your study. If you are not sure if a list item applies to your research, read the appropriate section before selecting a response.

Materials & experimental systems

n/a	Involved in the study	
☒	☐	Antibodies
☒	☐	Eukaryotic cell lines
☒	☐	Palaeontology and archaeology
☒	☐	Animals and other organisms
☐	☒	Clinical data
☒	☐	Dual use research of concern

Methods

n/a	Involved in the study	
☒	☐	ChIP-seq
☒	☐	Flow cytometry
☒	☐	MRI-based neuroimaging

Clinical data

Policy information about clinical studies

All manuscripts should comply with the ICMJE guidelines for publication of clinical research and a completed CONSORT checklist must be included with all submissions.

Clinical trial registration	NCT00928473
Study protocol	Study protocol for the discovery cohort and replication cohort in children is available upon request to Jens-Christian Holm, jhom@regionsjaelland.dk.<br>Study protocol for the replication cohort is available upon request to Odense Patient Data Exploratory Network (open@rsyd.dk) with reference to project ID OP_040.
Data collection	Subjects in the discovery cohort were recruited between January 2009 and April 2019 from 1) an obesity clinic cohort recruited from a multidisciplinary childhood obesity management program at Copenhagen University Hospital Holbæk and 2) a population-based cohort recruited from schools in 11 municipalities across Zealand, Denmark in a cross-sectional study design. Demographics, clinical data and blood plasma samples were collected upon recruitment.

Outcomes	Not applicable
